# Supplementary material for: Iridium-Catalyzed Highly Selective 1,4-Reduction of α,β-Unsaturated Carbonyl Compounds
Source: Molecules. 2024 Dec 14;29(24):5912. doi: 10.3390/molecules29245912 (PMC11678408; doi:10.3390/molecules29245912)

Supplementary Materials

for

Iridium-Catalyzed Selective 1,4-Reduction of α,β-Unsaturated Carbonyl Compounds

Youwei Chen,[a] Jide Li,[a] Jiaxi Xu[a] and Zhanhui Yang*[a]

[a]*Department of Organic Chemistry, College of Chemistry, Beijing University of Chemical Technology, Beijing 100029, P. R. China.*

Correspondence: zhyang@mail.buct.edu.cn

Contents

1. The iridium-catalyzed transfer hydrogenation of **1r**, **1s**, **1u** and **1t** S3

2. ^{1}H NMR spectra of deuterium labelling experiments S7

3. GC-MS spectra for gram-scale reaction S11

4. Copies of ^{1}H and ^{13}C NMR spectra of starting materials S12

5. Copies of ^{1}H and ^{13}C NMR spectra of catalyst C1 and isolated products.. S18

6. Copies of ^{1}H NMR spectra of crude reaction mixtures in Table 1 .... S29

1. The iridium-catalyzed transfer hydrogenation of 1r, 1s, 1u and 1t

(*E*)-Chalcone (**1r**) was reduced to 1,3-diphenylpropan-1-one (**2r**), 1,3-diphenylpropan-1-ol (**2ra**),and (*E*)-1,3-diphenylprop-2-en-1-ol (**2rb)** under Reaction Conditions C in 60%, 17%, and 23% yields, respectively.

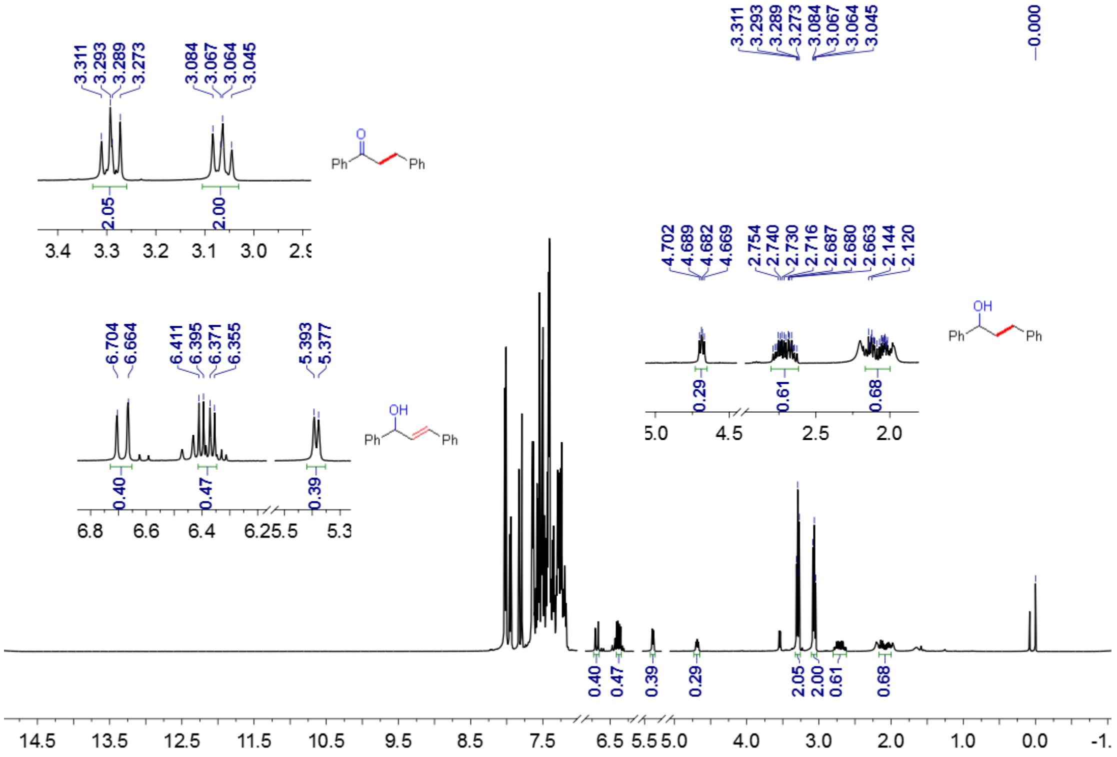

1-Phenylprop-2-en-1-one [5] (**1s**) was reduced to propiophenone (**2s)** and 1-phenylpropan-1-ol (**2sa)** under ***Conditions C*** in respective 18% and 75% yields.

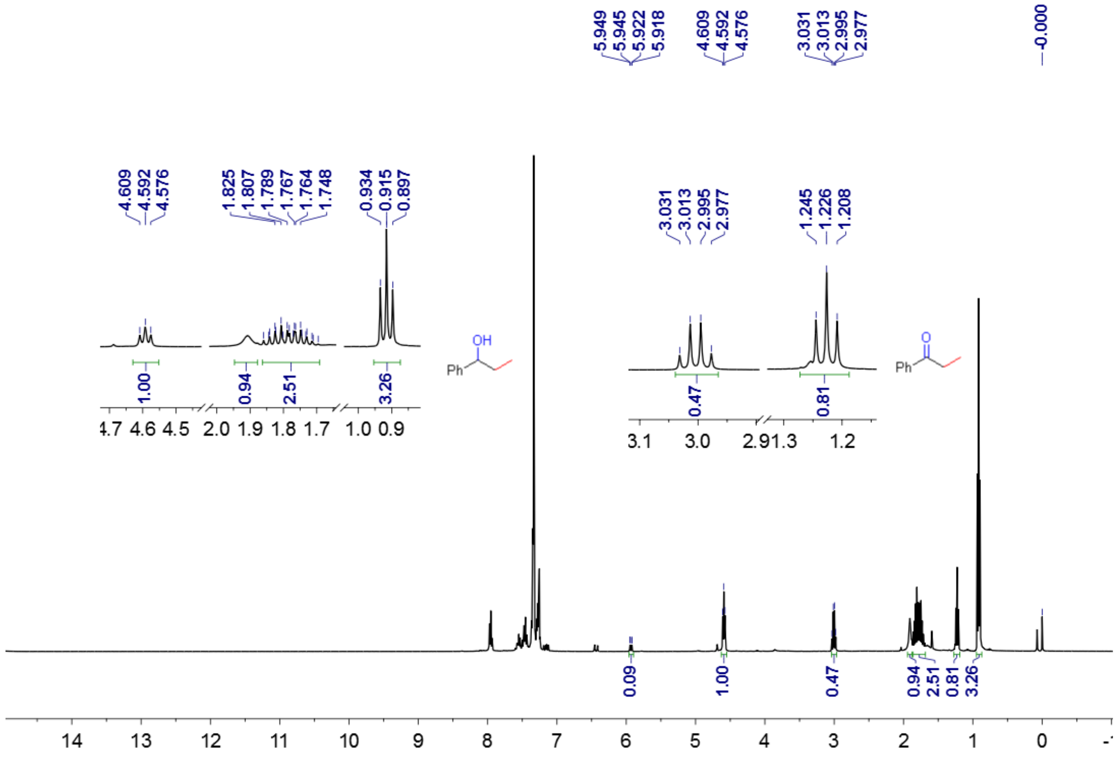

Cinnamic aldehyde (**1u**) was reduced to 3-phenylpropan-1-ol (**2ua)** and 3-phenylpropyl formate (**2ua')** under ***Condition D*** in respective 45% and 53% yields.

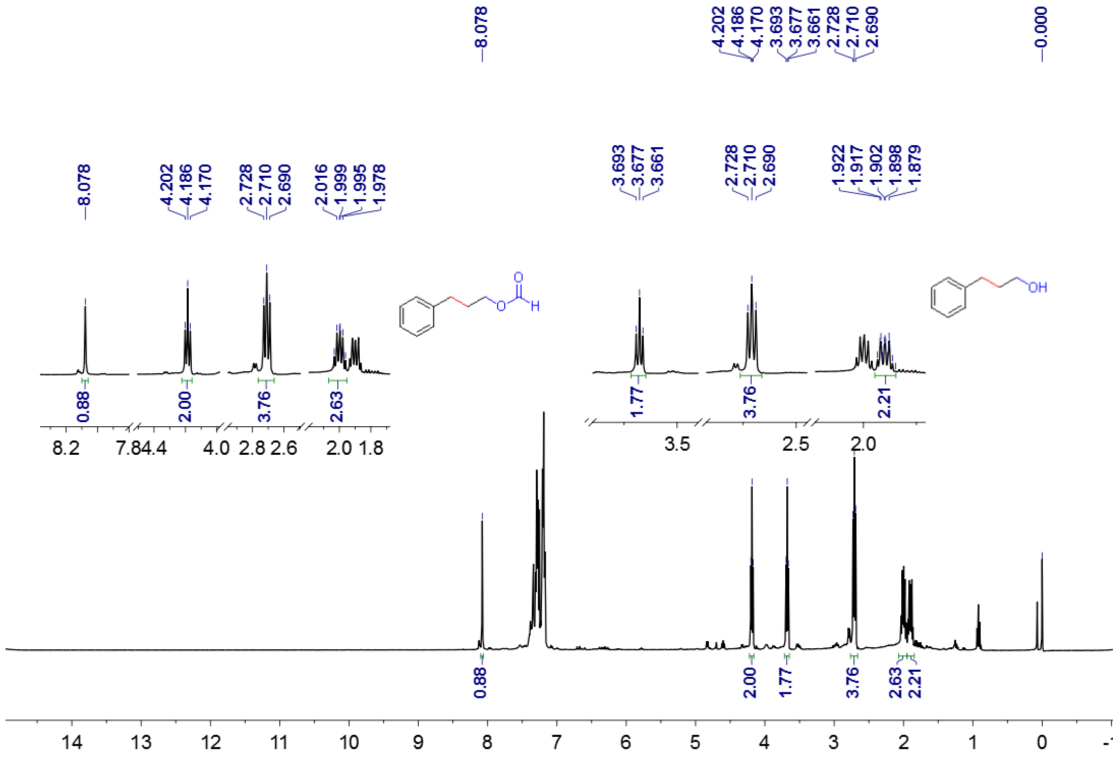

Methacrylaldehyde (**1t**) (1 mmol) was completely reduced to isobutanol (**2ta**) under ***Condition C***.

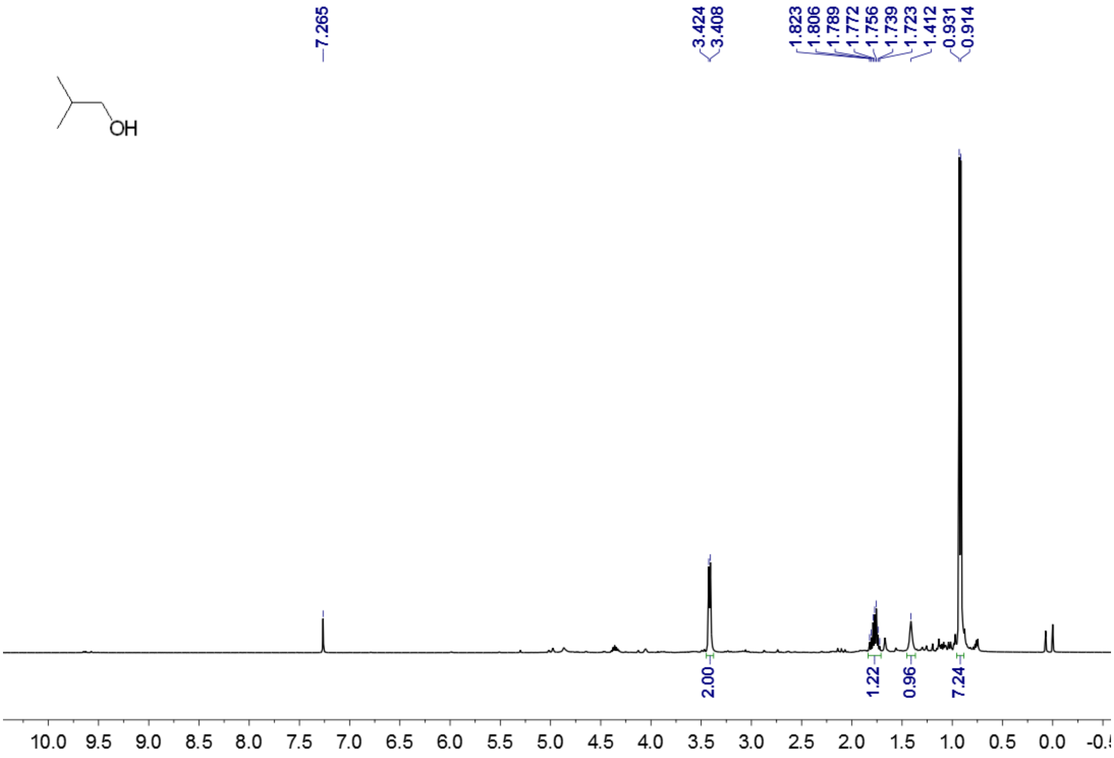

2. ¹H NMR spectra of deuterium labelling experiments

Standard spectrum of product **2a**.

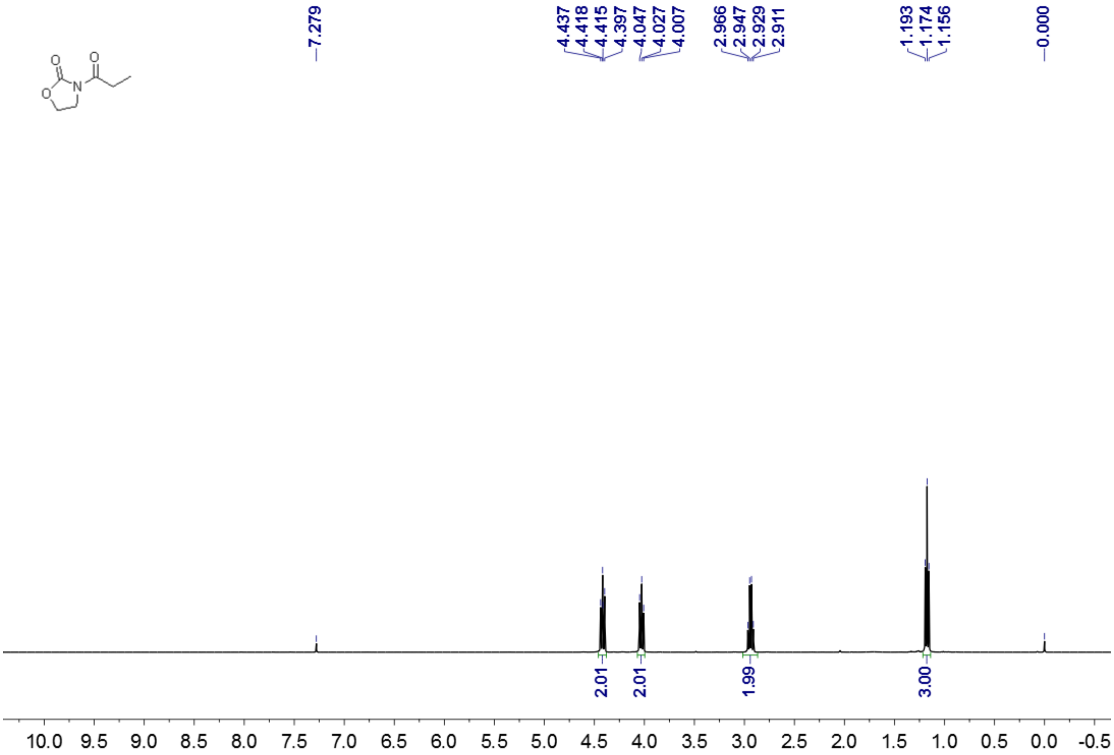

The deuterium incorporations at α- and β-positions, namely α-[D] and β-[D], were calculated based on the above standard spectrum.

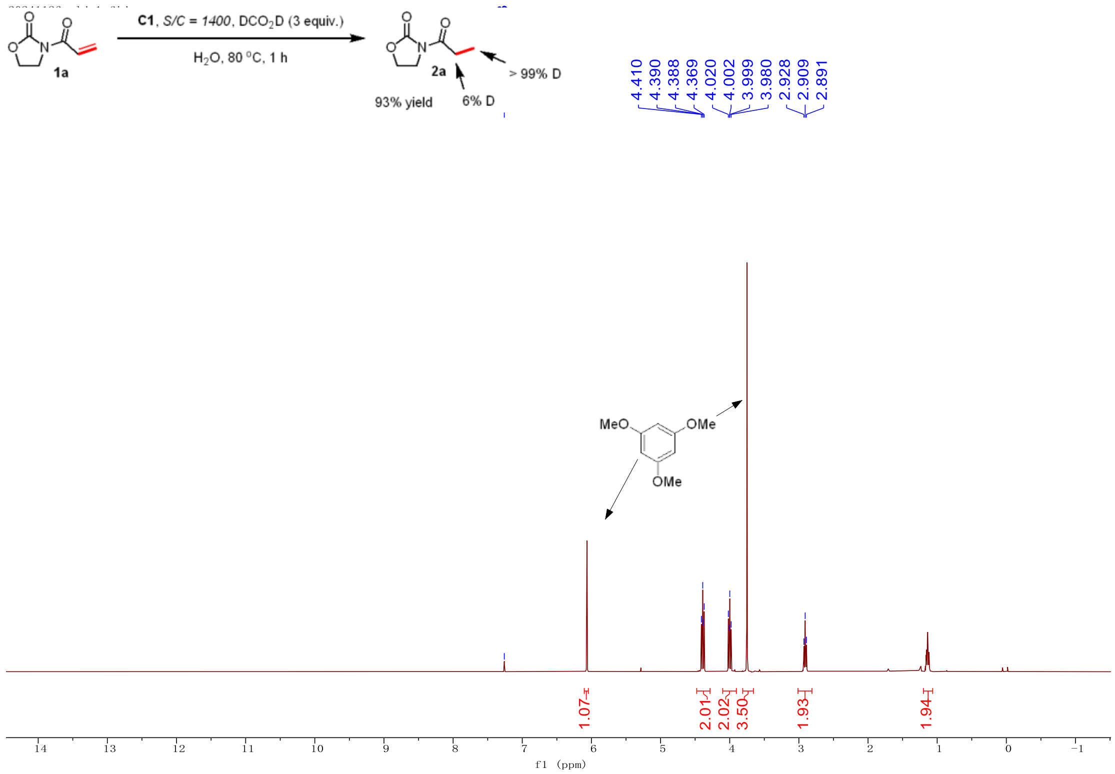

α-[D] = (1.99-1.93)/1 = 6%;

β-[D] = (3.00-1.94)/1 = >99%

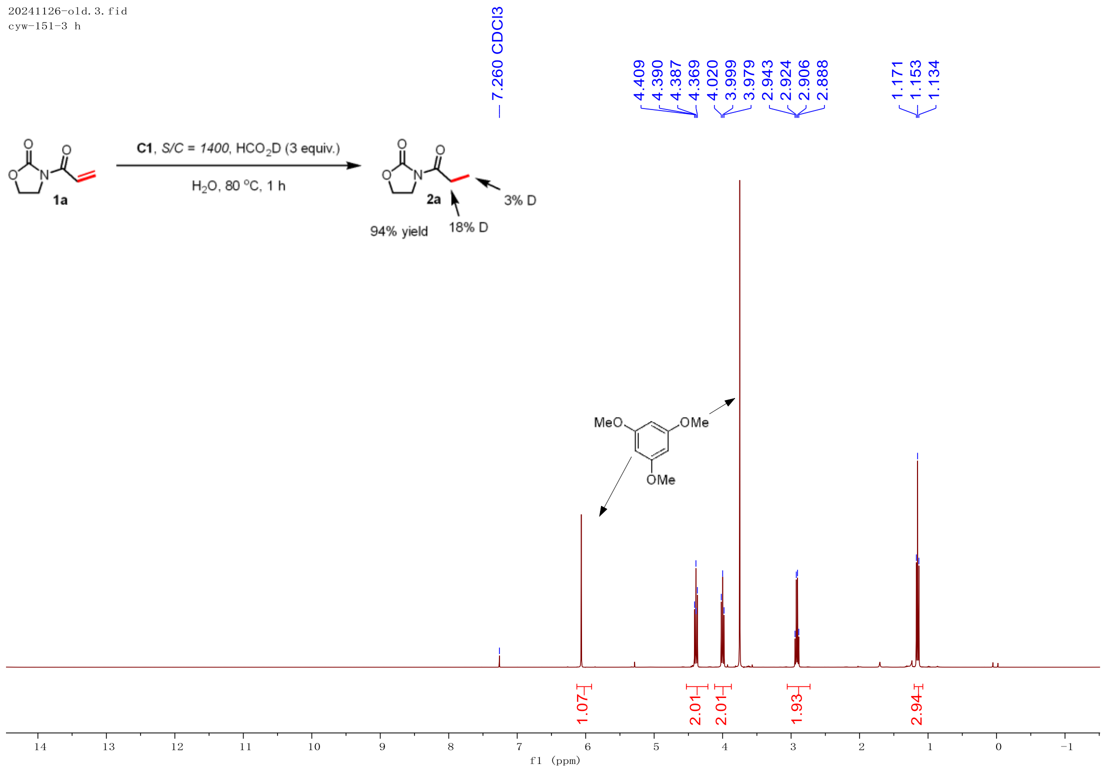

α-[D] = (1.99-1.93)/1 = 6%

β-[D] = (3.00-2.94)/1 = 6%

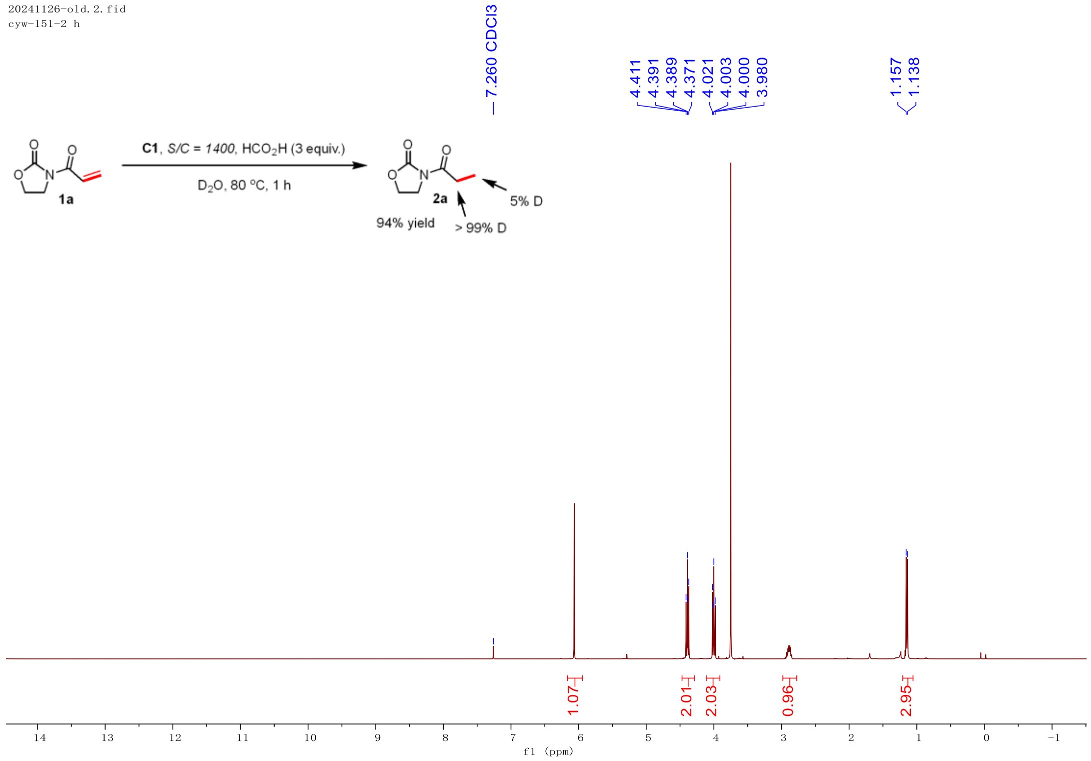

α-[D] = (1.99-0.96)/1 >99%;

β-[D] = (3.00-2.95)/1 = 5%

3. GC-MS spectra for gram-scale reaction

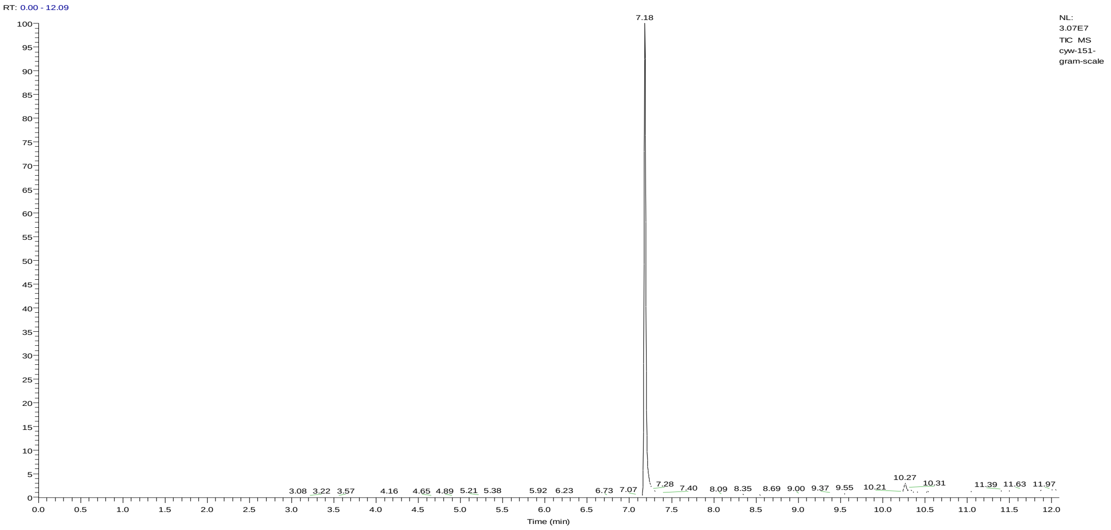

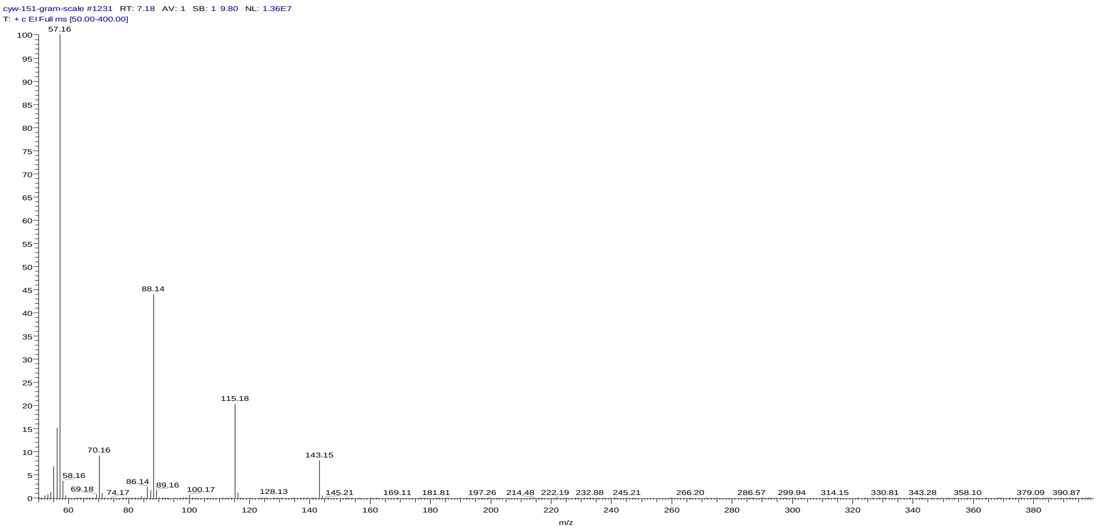

4. Copies of ^{1}H and ^{13}C NMR spectra of starting materials

N, N-diethylacrylamide (1c) in $CDCl_3$

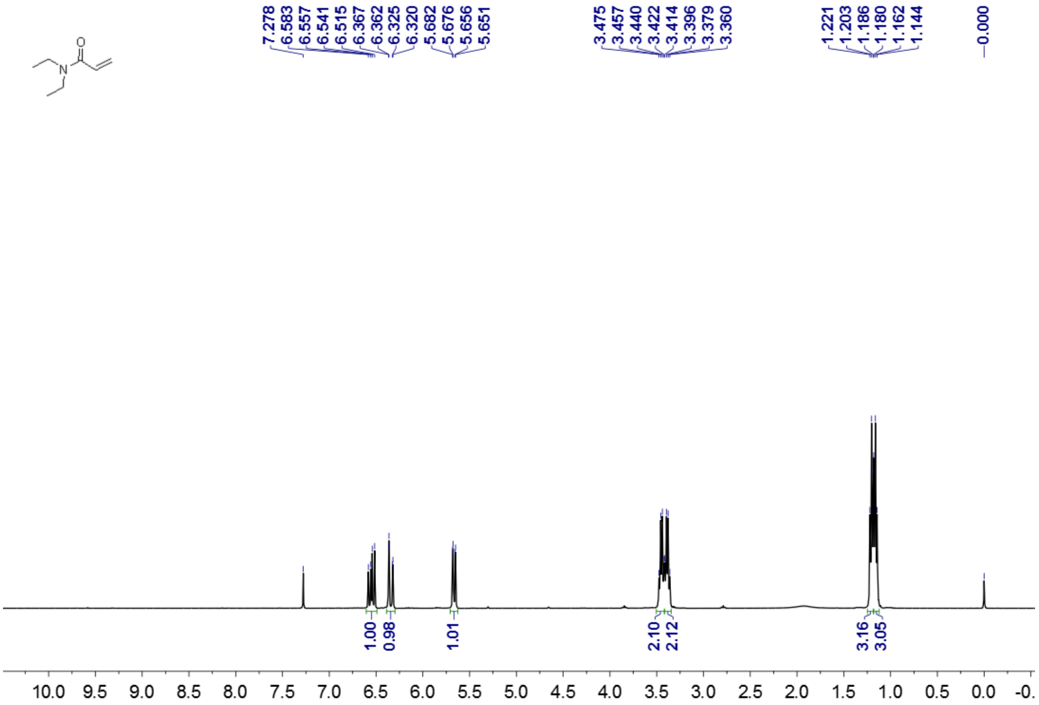

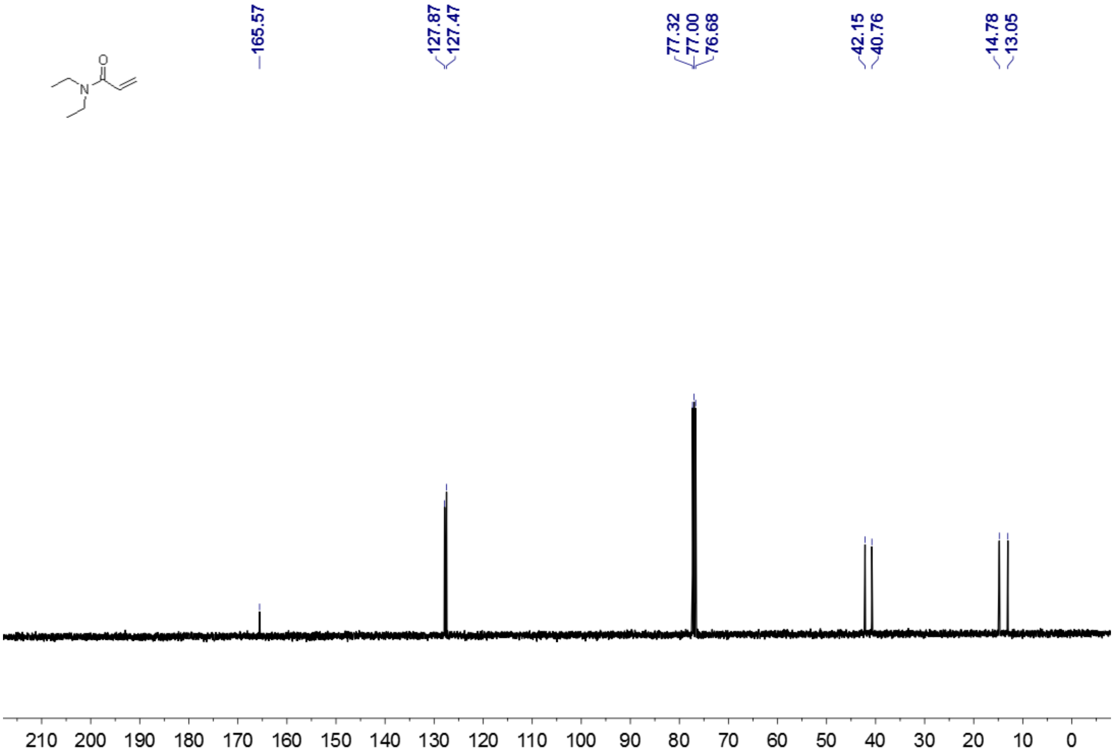

N-Acryloylpyrrolidine (1d) in $CDCl_3$

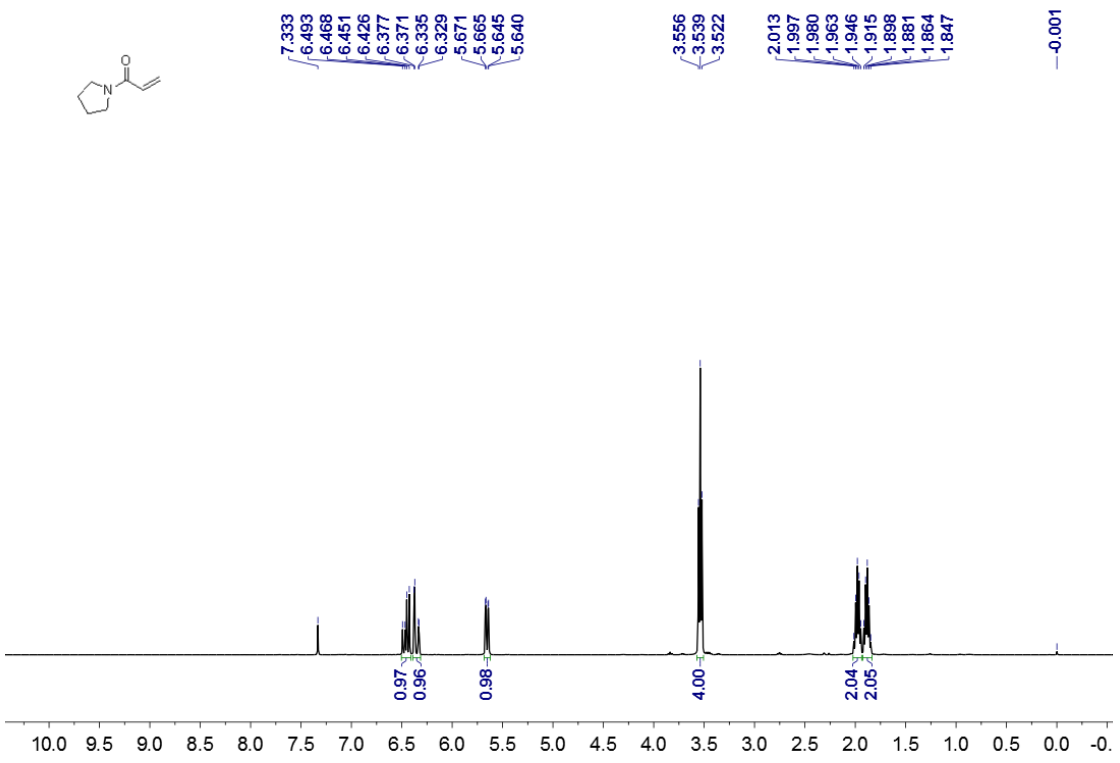

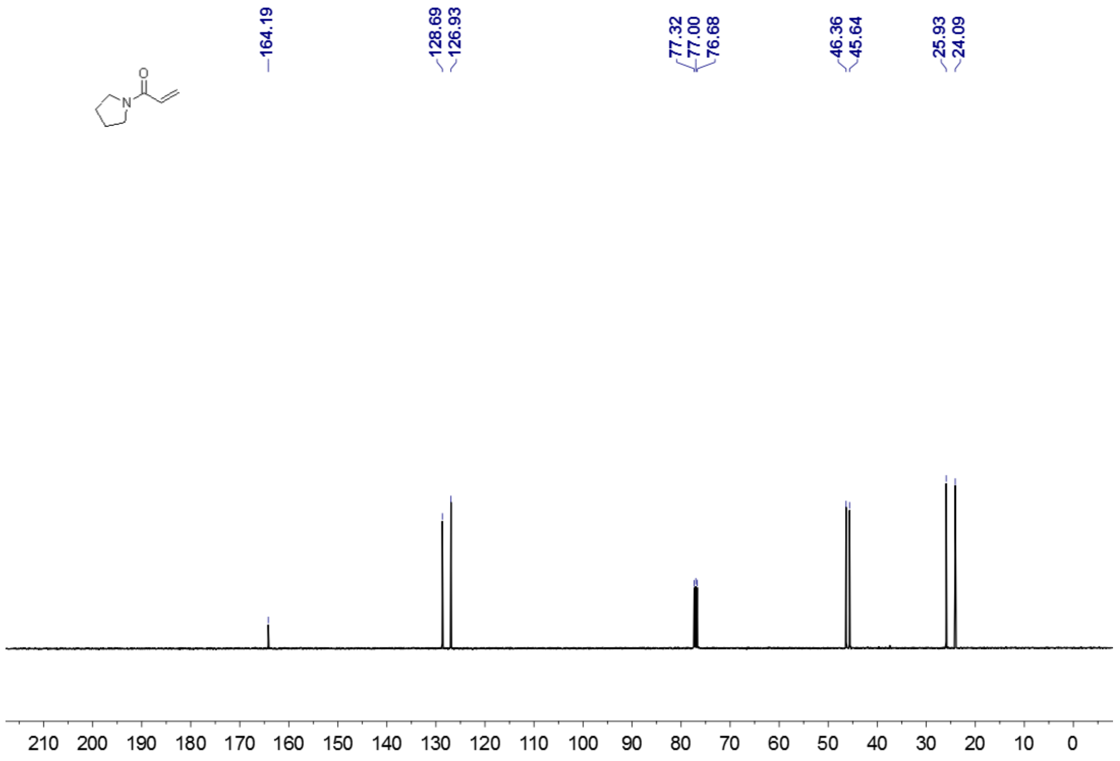

N,N-Dibenzylacrylamide (1e) in $CDCl_3$

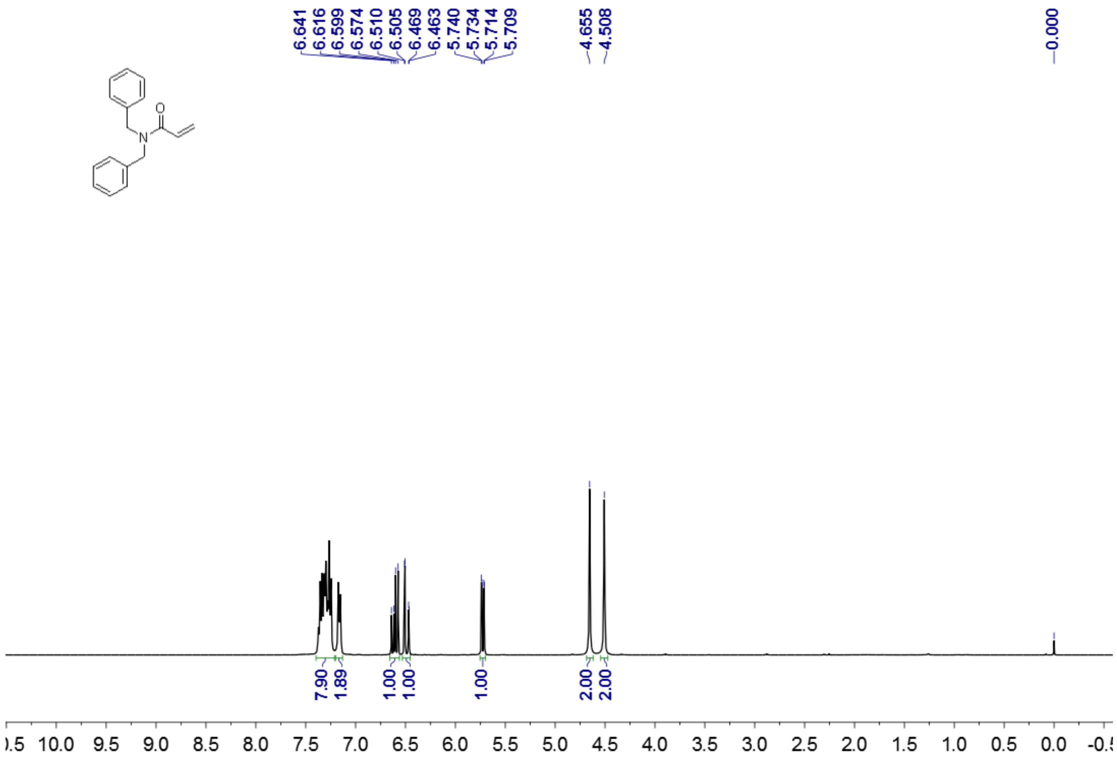

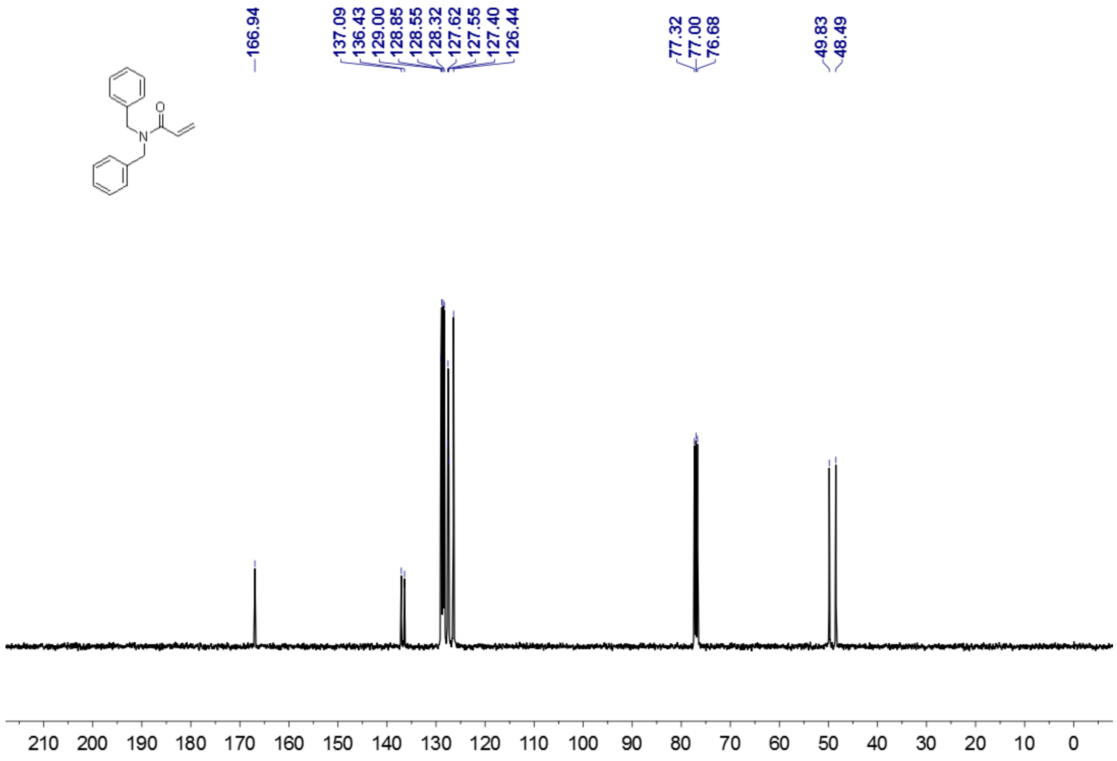

N-Methyl-*N*-phenylacrylamide (2f) in $CDCl_3$

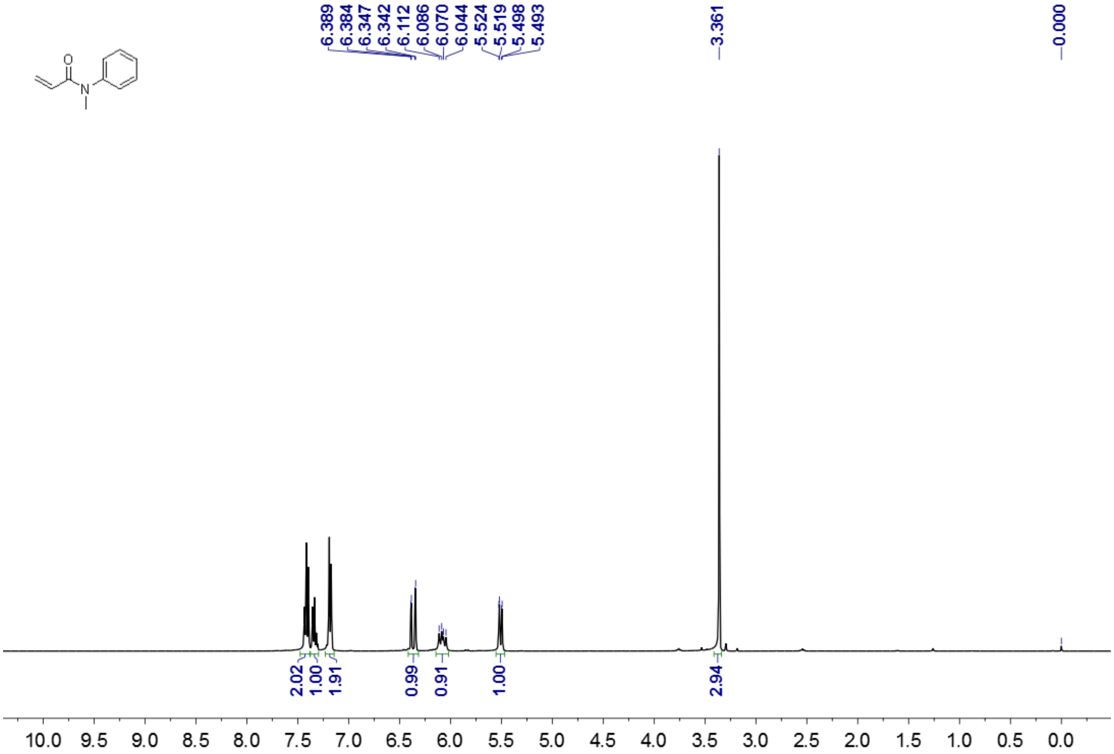

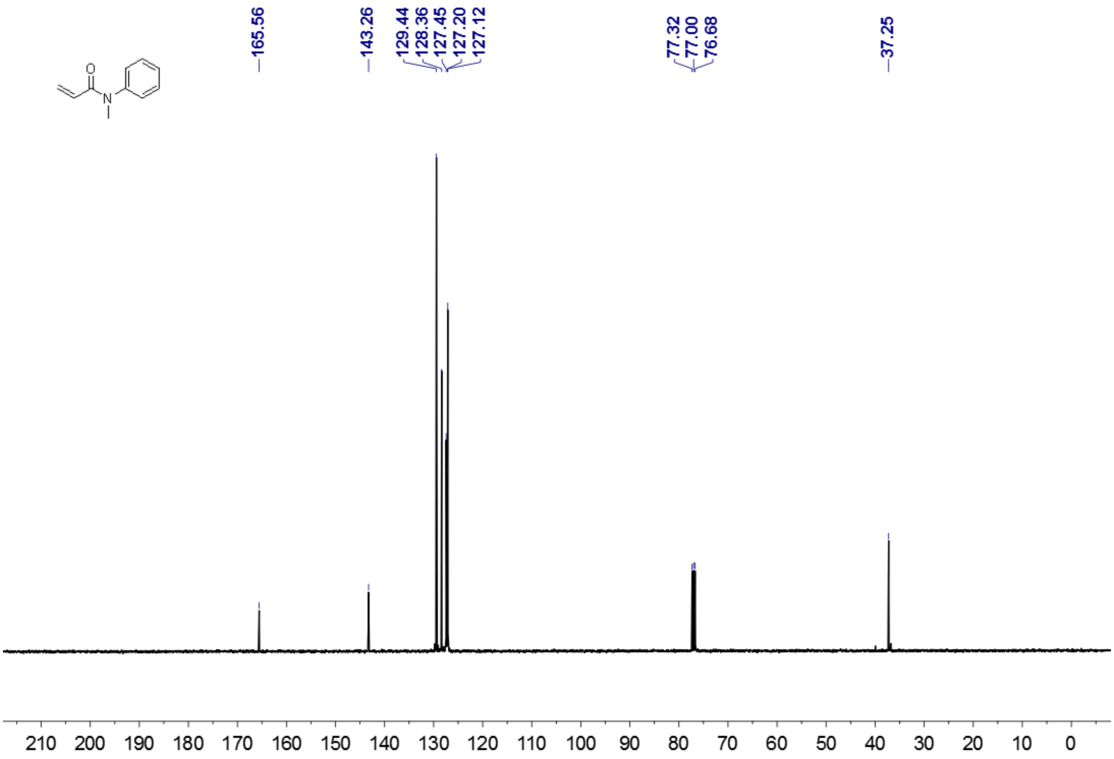

N,N-Diphenylacrylamide (1g) in $CDCl_3$

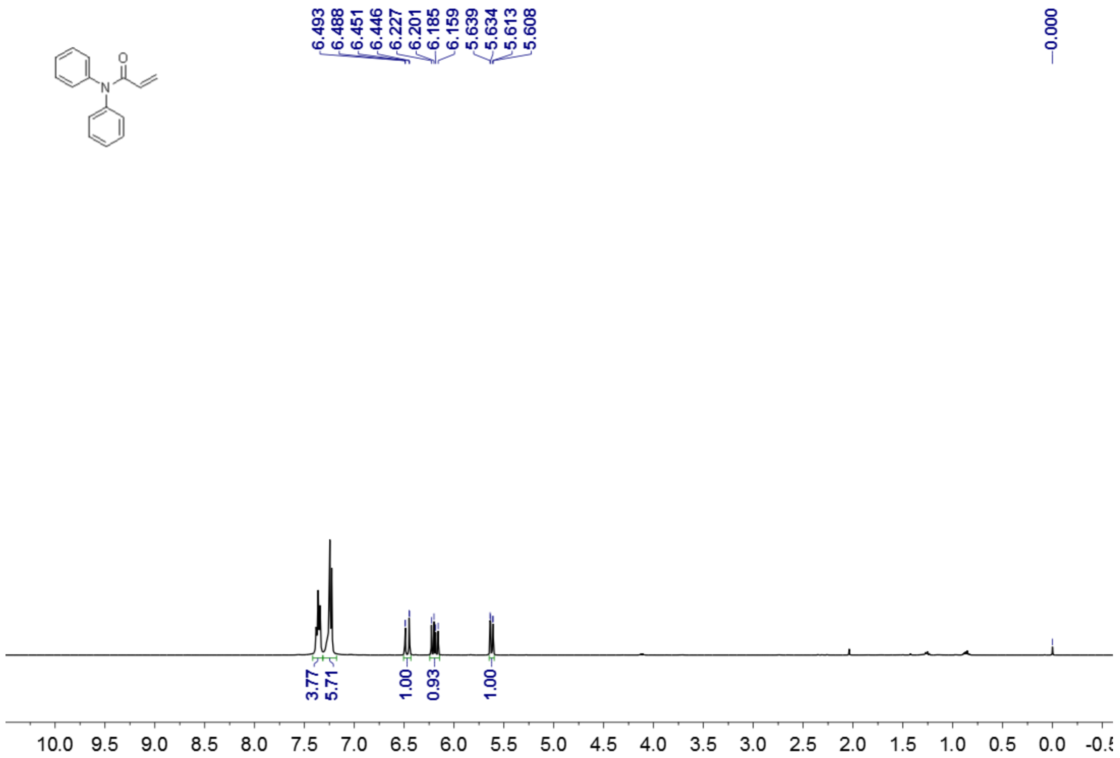

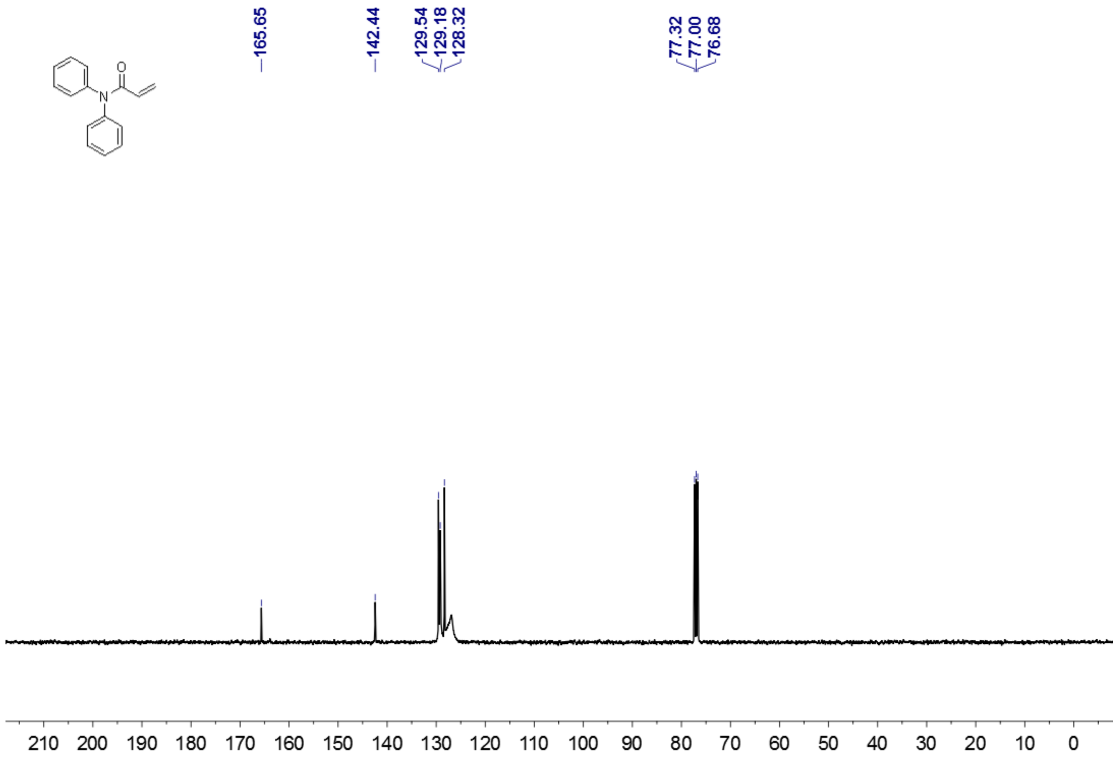

(Vinylsulfonyl)benzene (1q) in $CDCl_3$

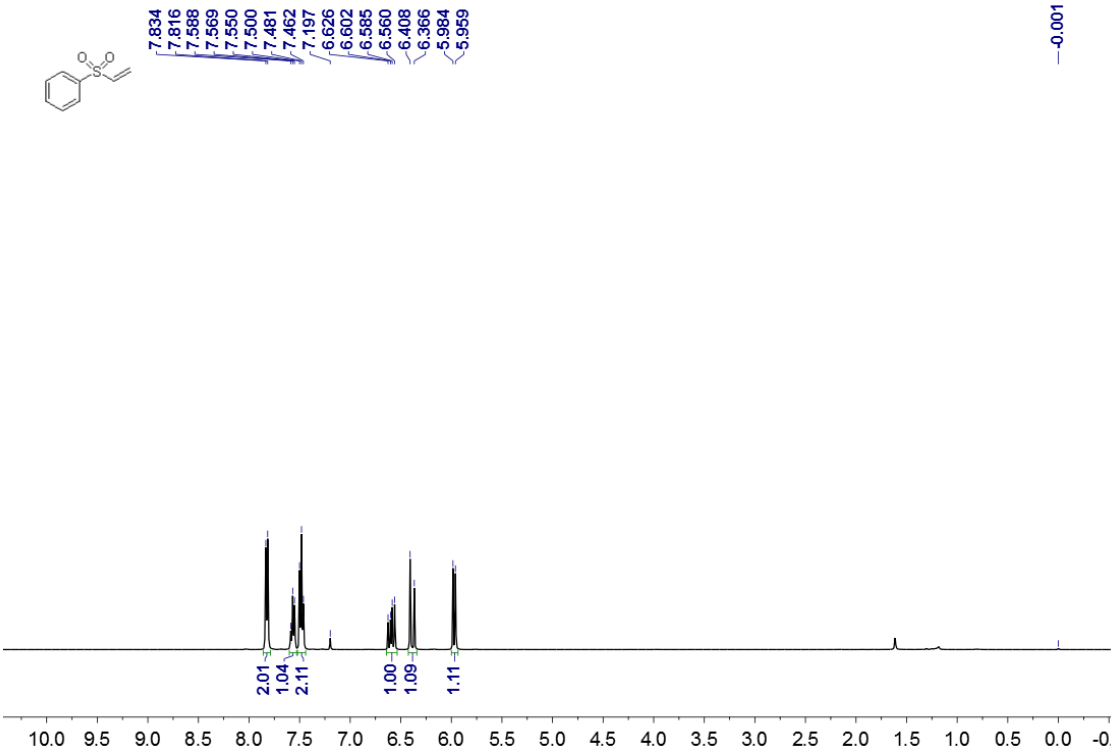

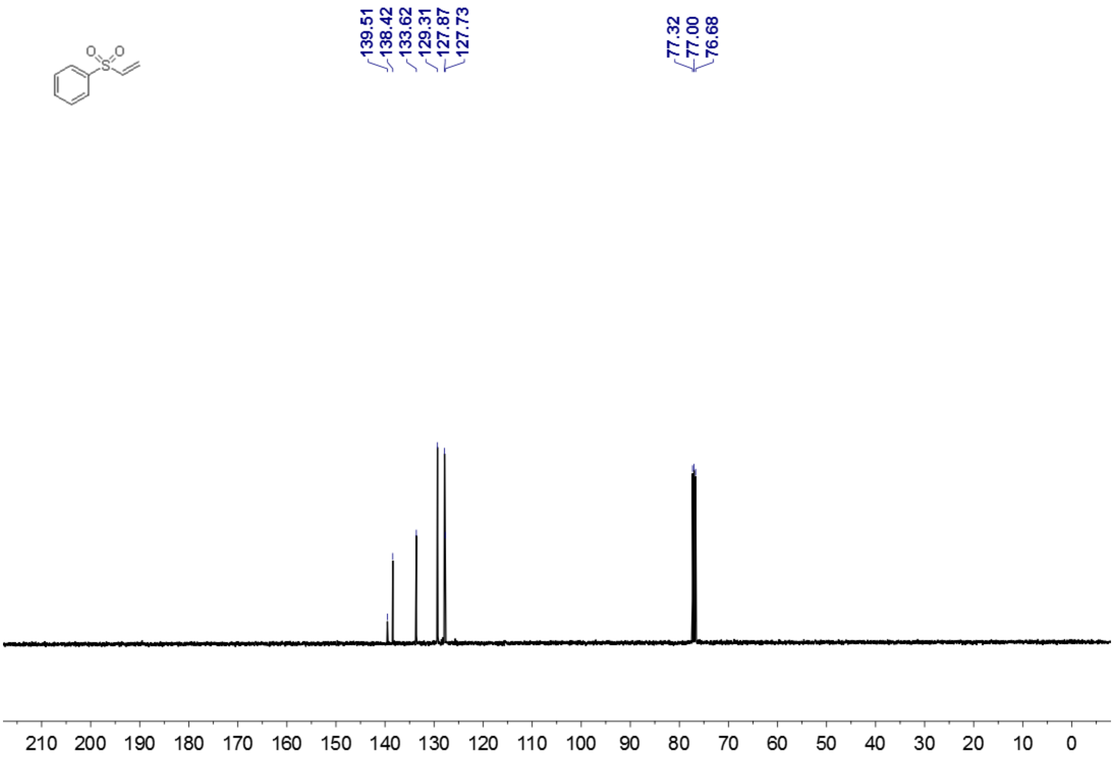

5. Copies of ^{1}H and ^{13}C NMR spectra of catalyst C1 and isolated products

Catalyst C1 in D_2O

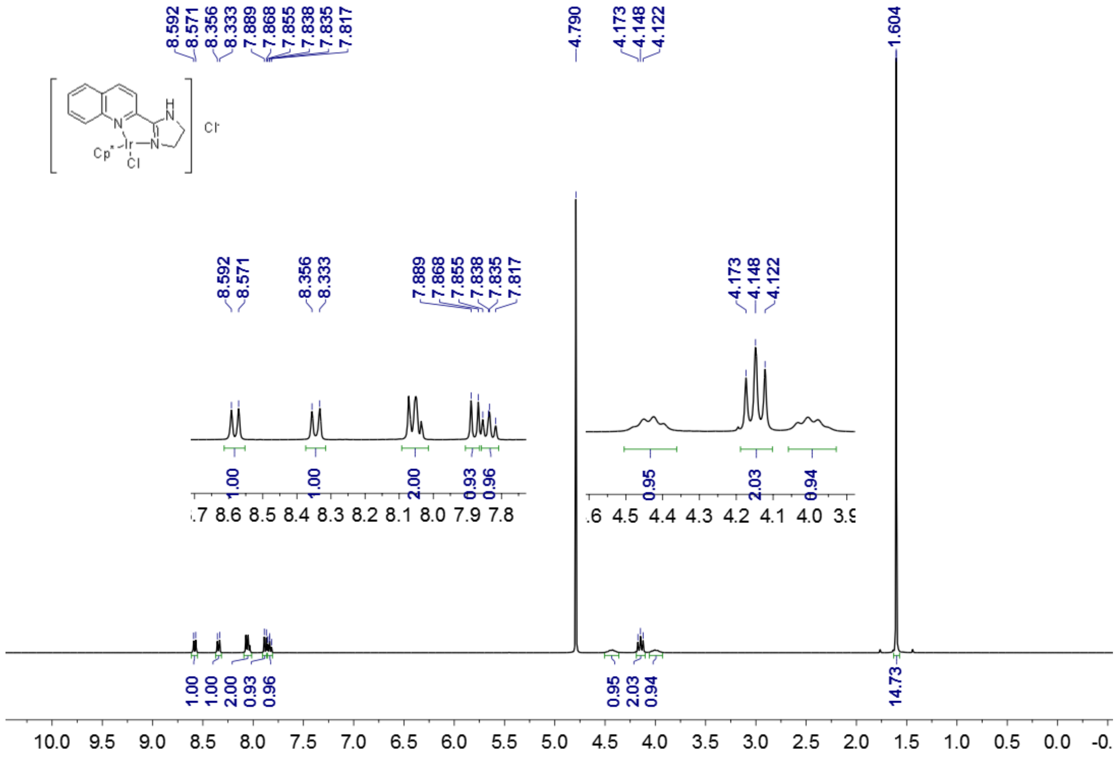

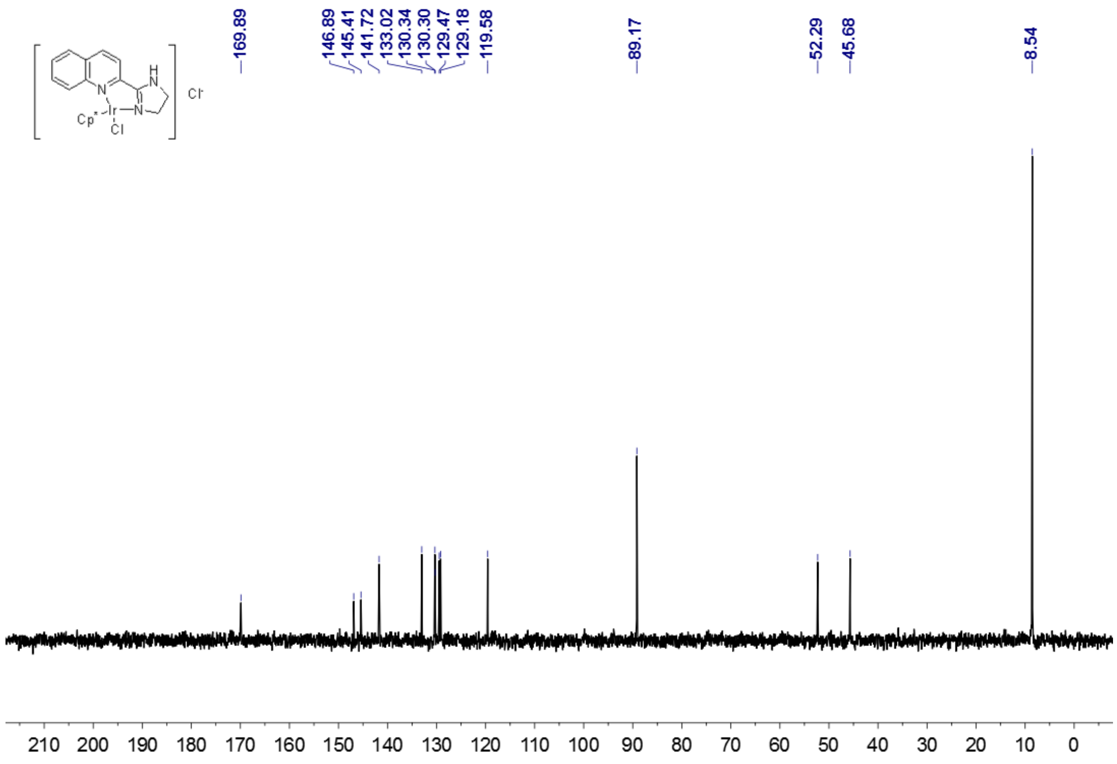

Cl-
Cp*
Ir
Cl
169.89
146.89
145.41
141.72
133.02
130.34
130.30
129.47
129.18
119.58
89.17
52.29
45.68
8.54
210 200 190 180 170 160 150 140 130 120 110 100 90 80 70 60 50 40 30 20 10 0

N-propanoyl-2-oxazolidinone (2a) in $CDCl_3$

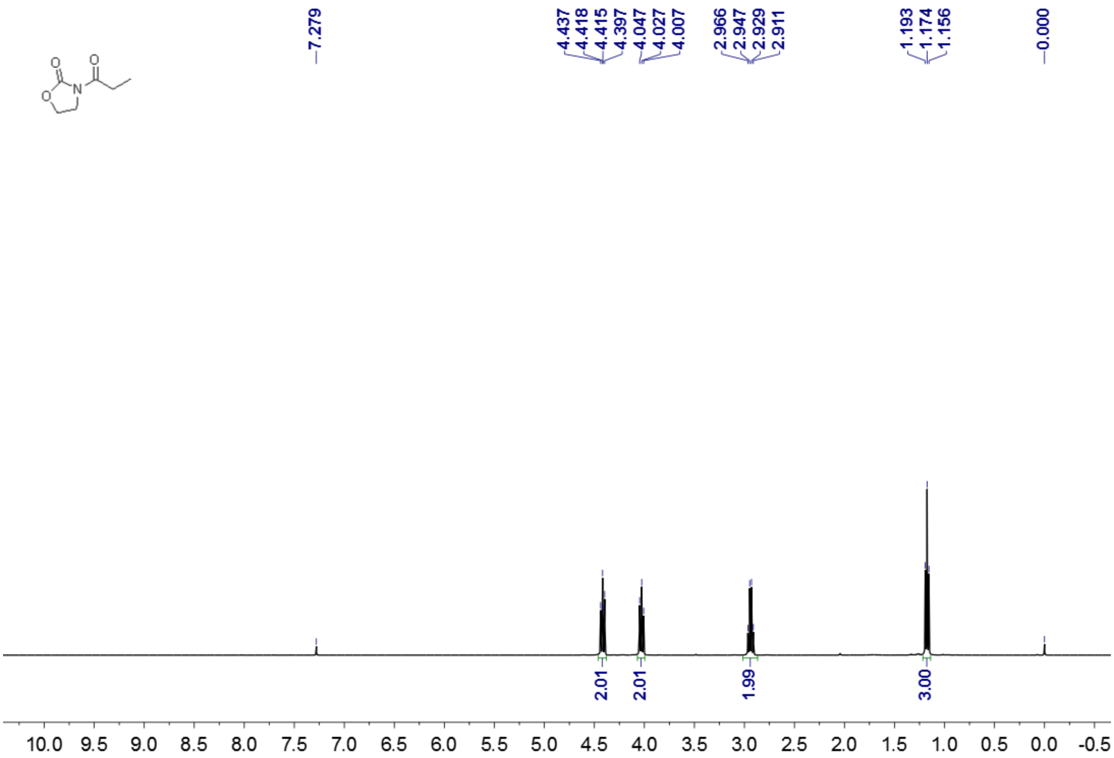

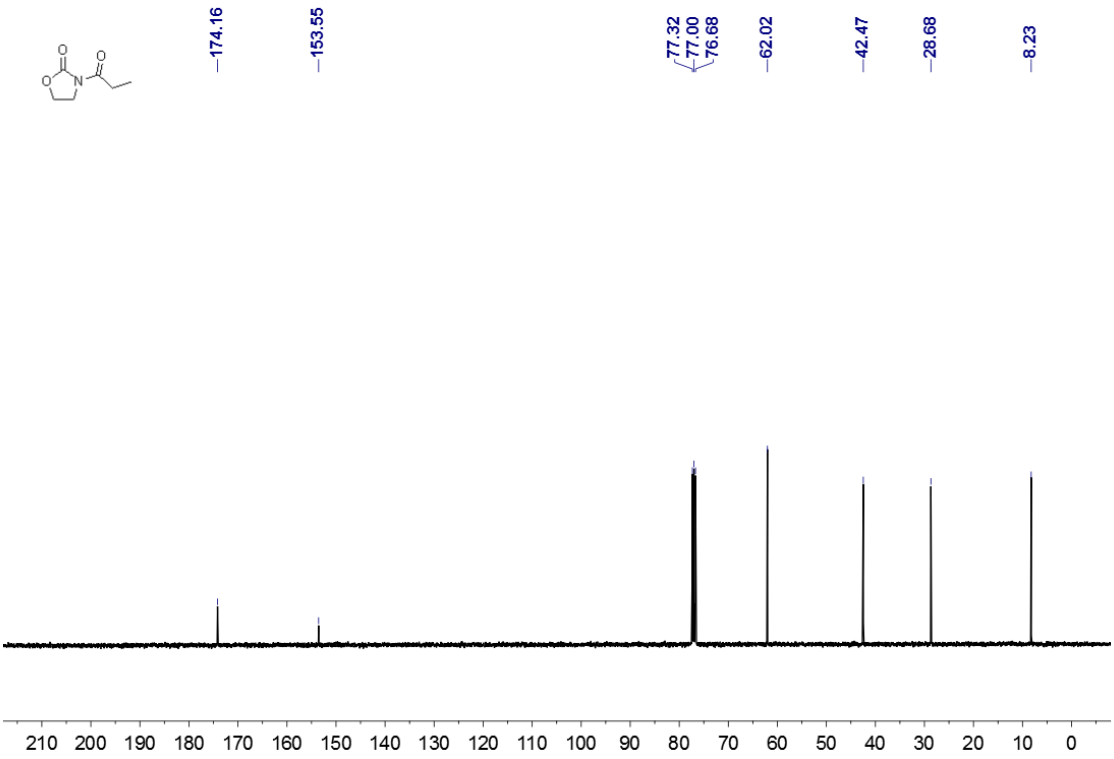

Propionamide (2b) in $CDCl_3$

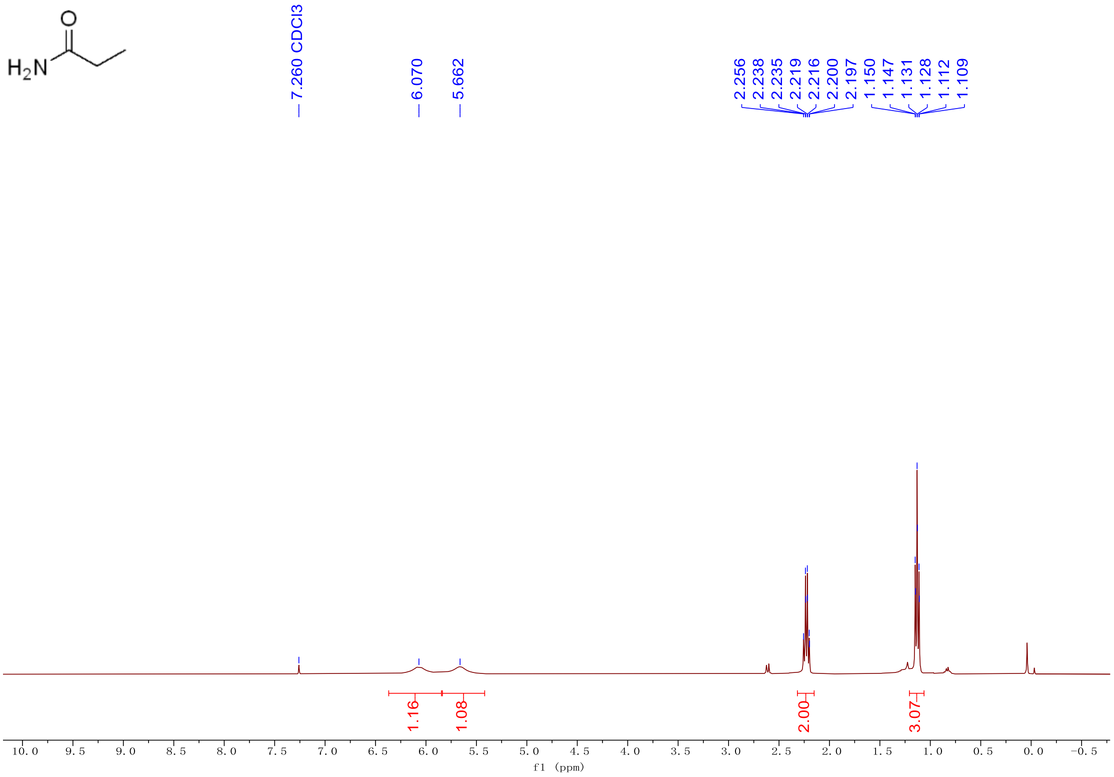

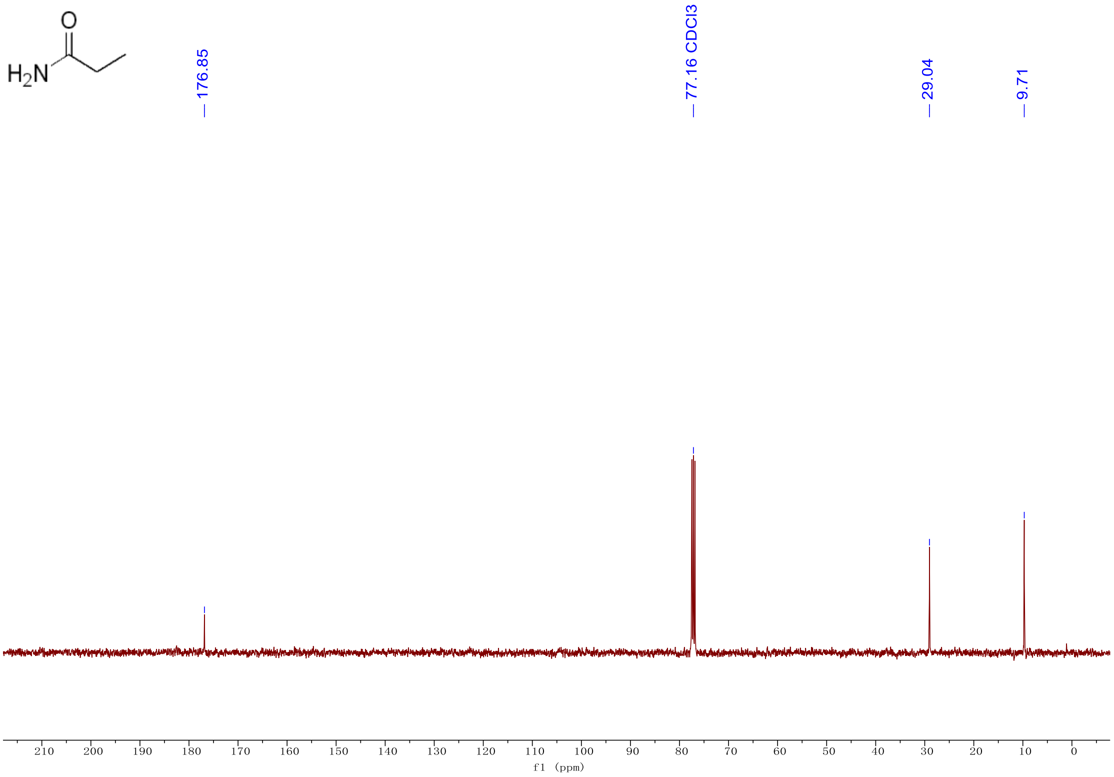

N,N-Diethylpropionamide (2c) in $CDCl_3$

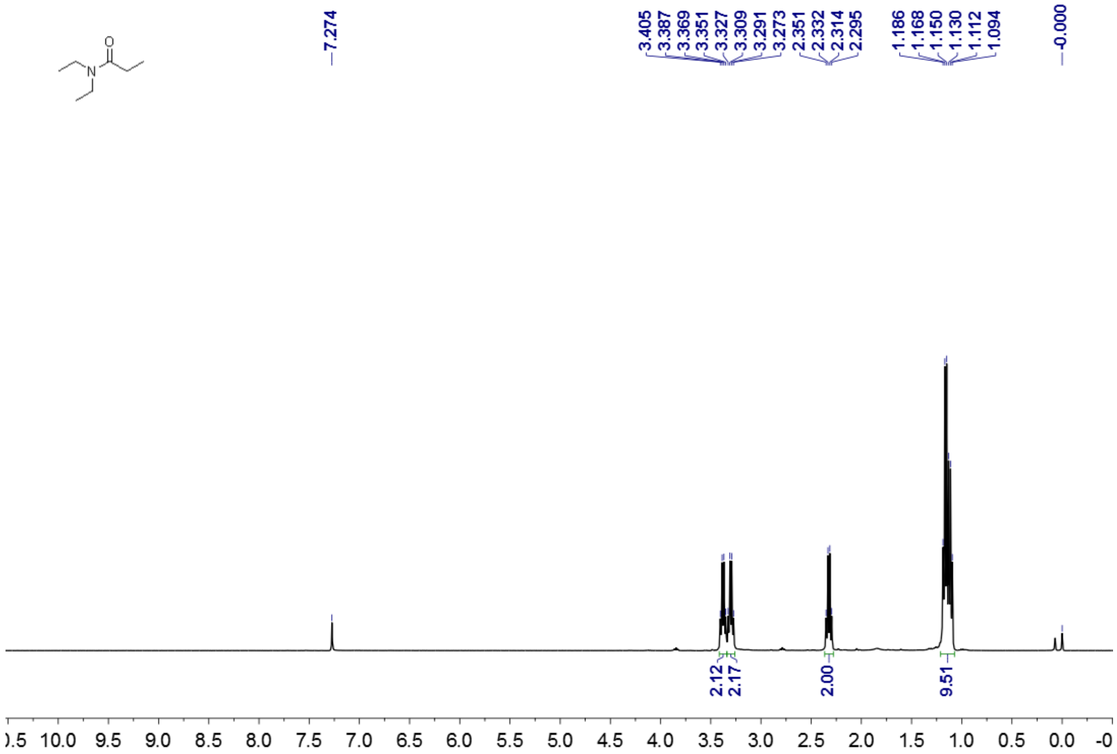

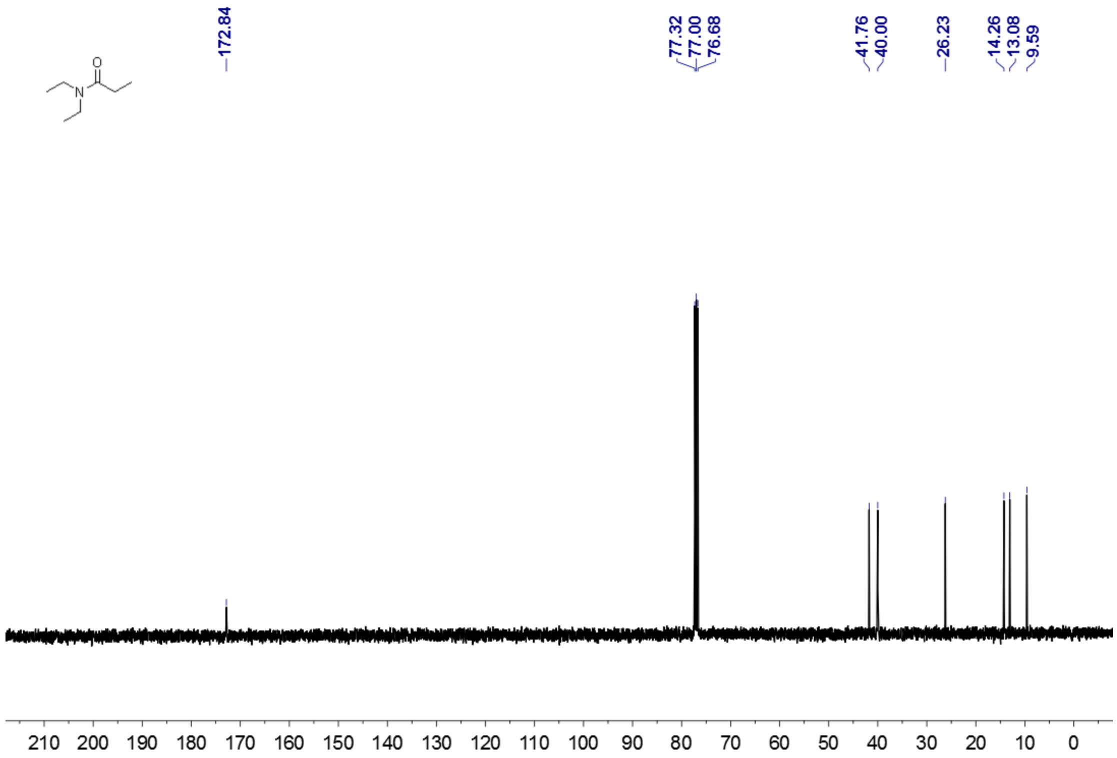

N-Propionylpyrrolidine (2d) in $CDCl_3$

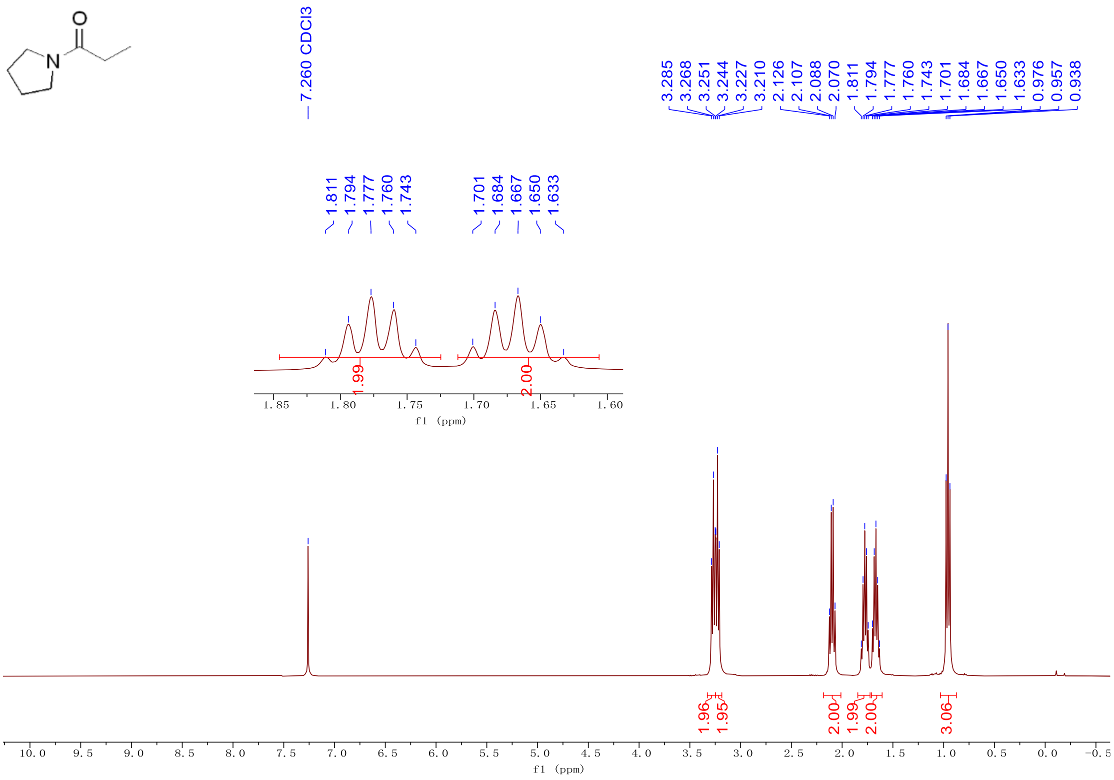

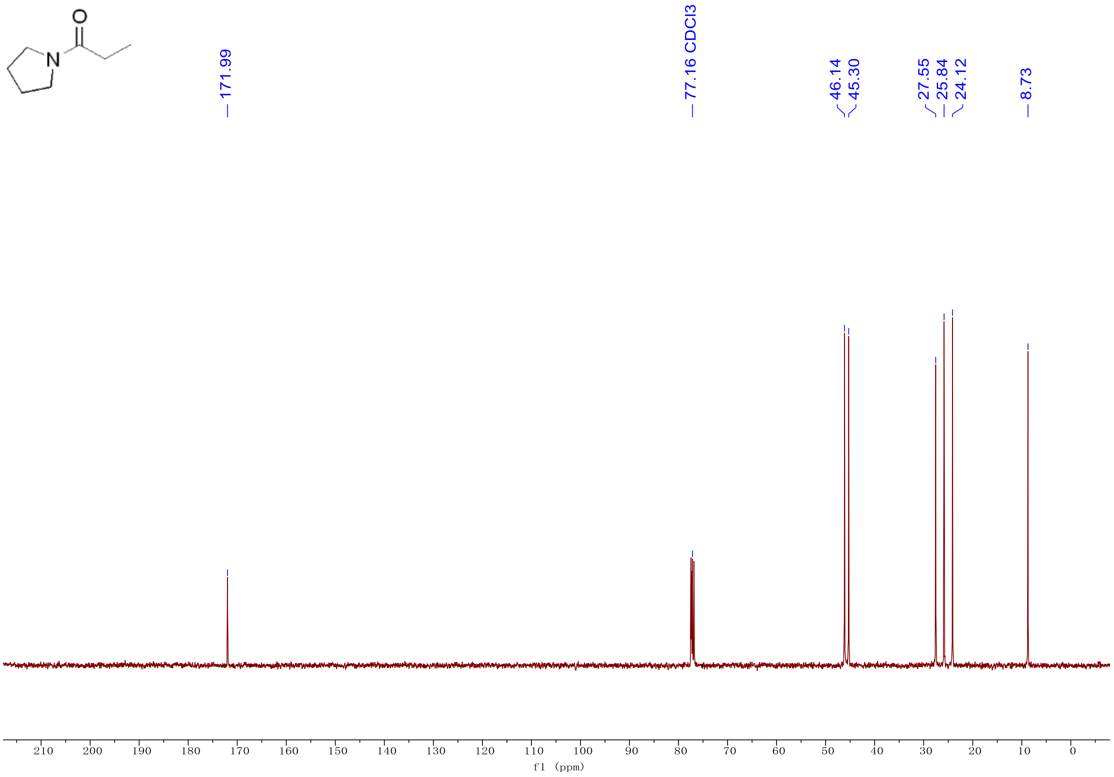

N,N-Diphenylpropionamide (2g) in $CDCl_3$

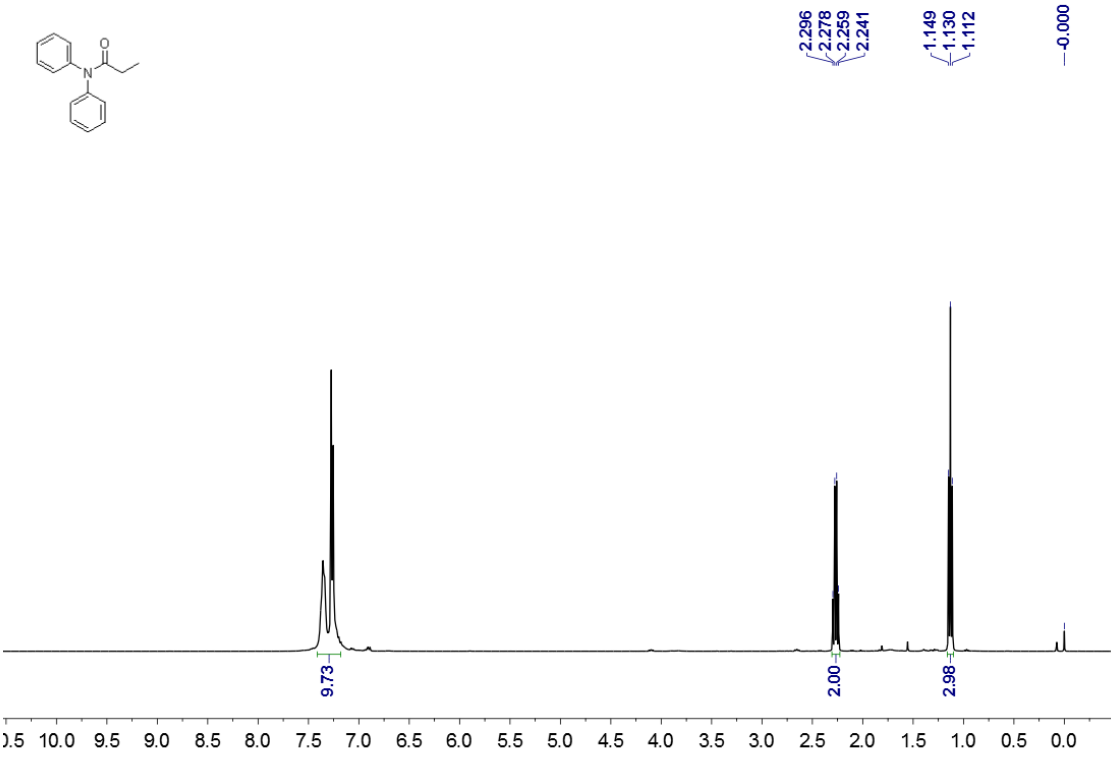

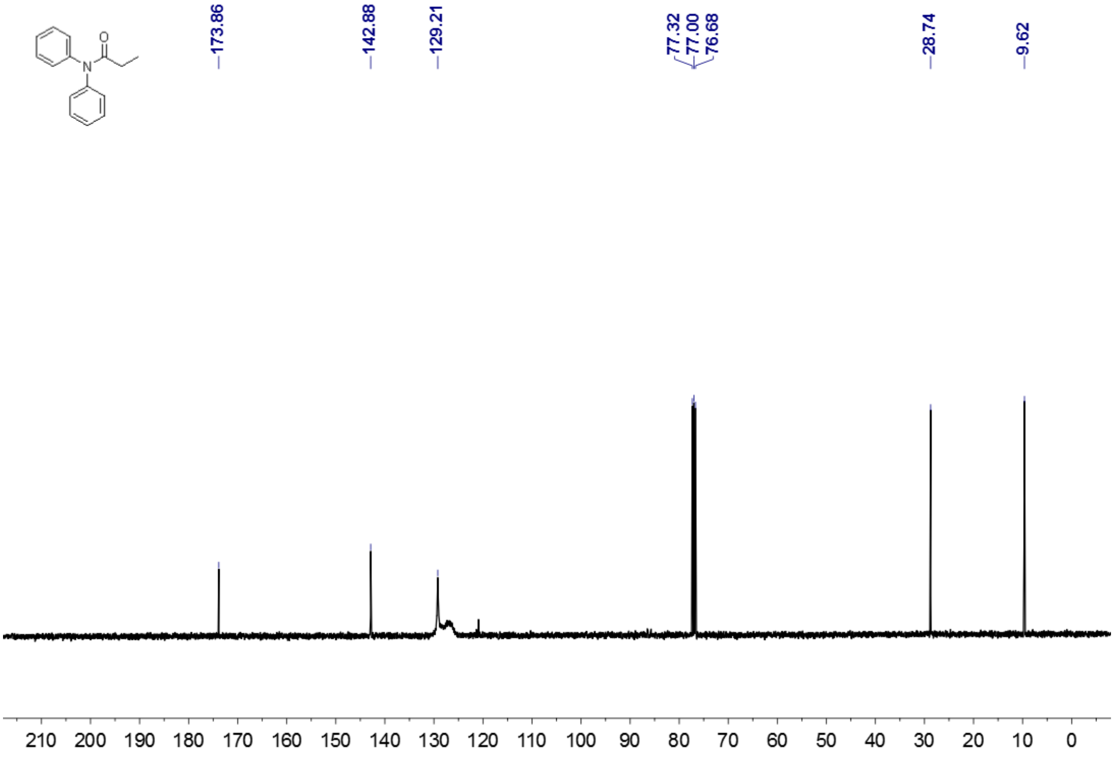

Isobutyramide (2h) in $CDCl_3$

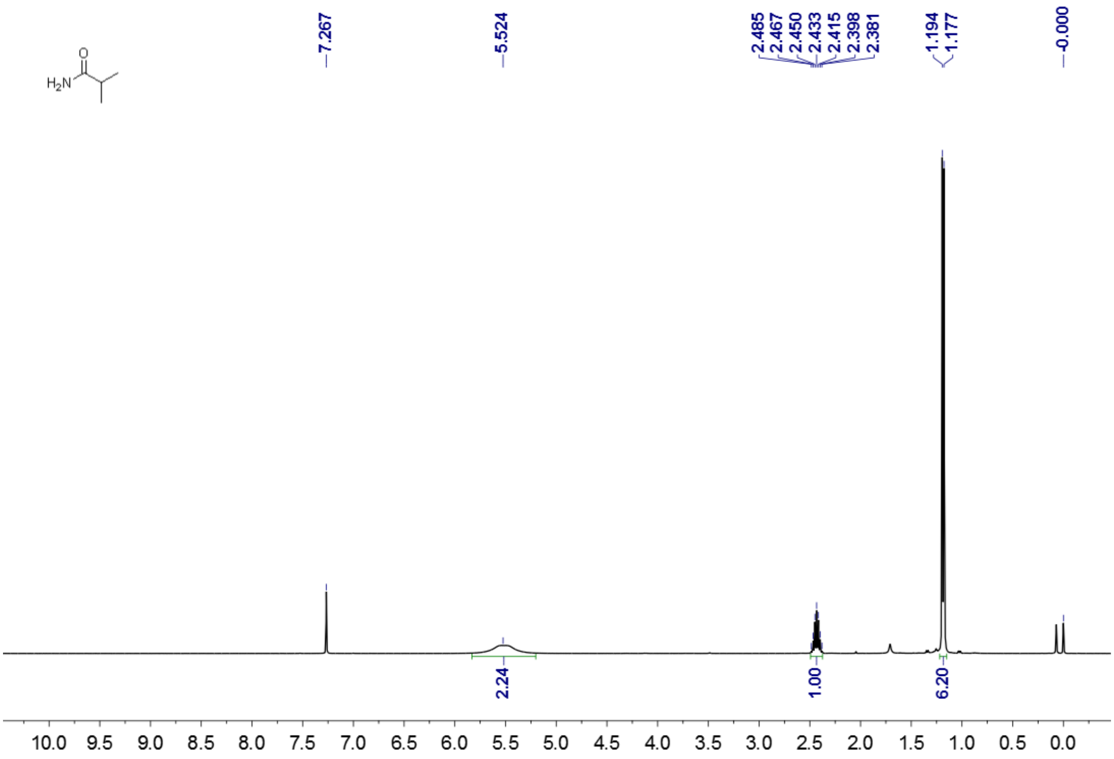

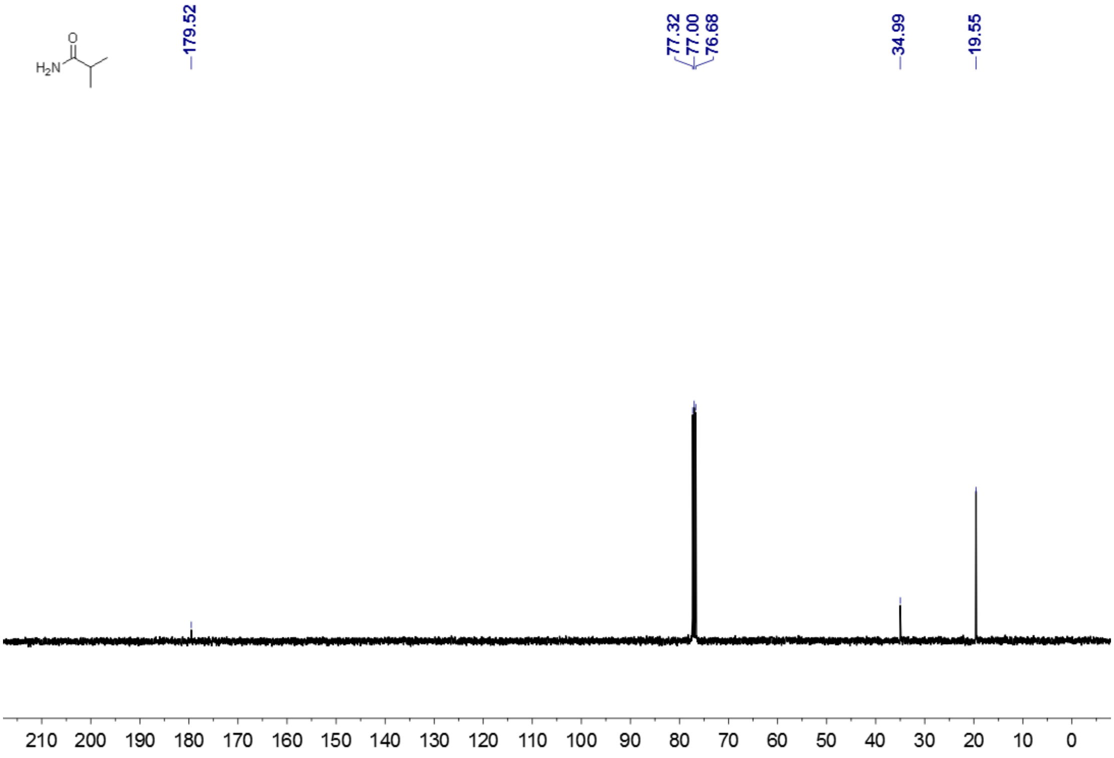

Pyrrolidine-2,5-dione (2j) in $CDCl_3$

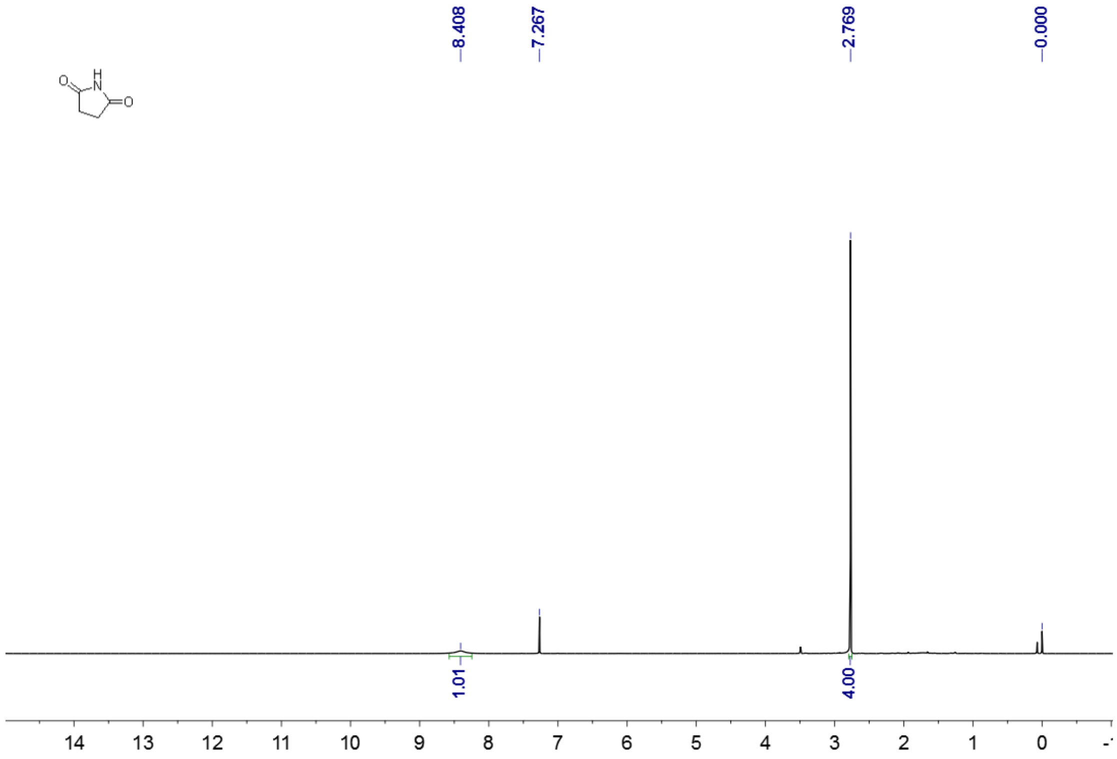

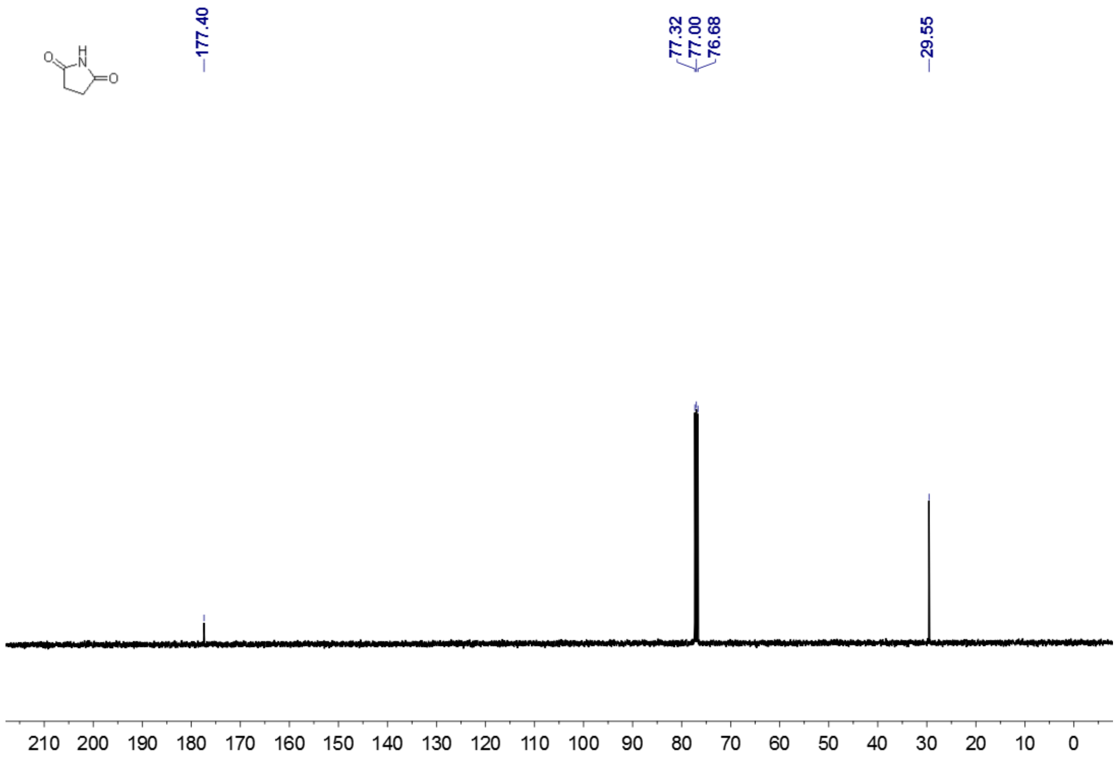

2-Phenylpropionic acid (2l) in $CDCl_3$

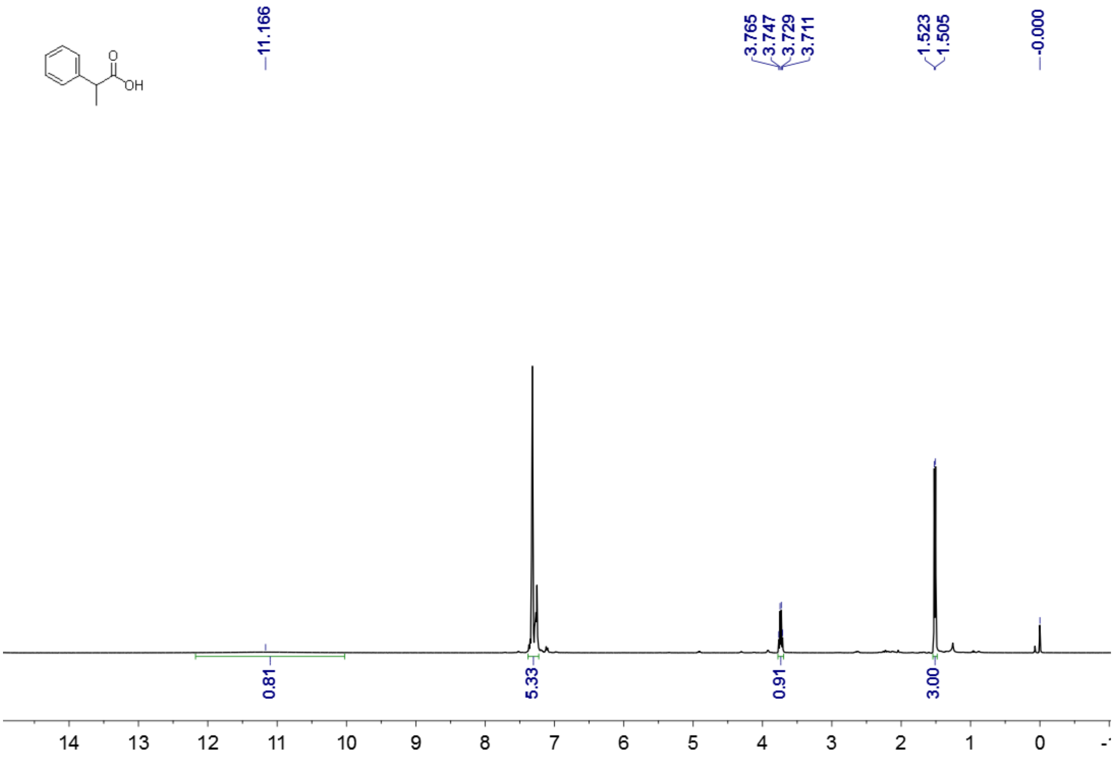

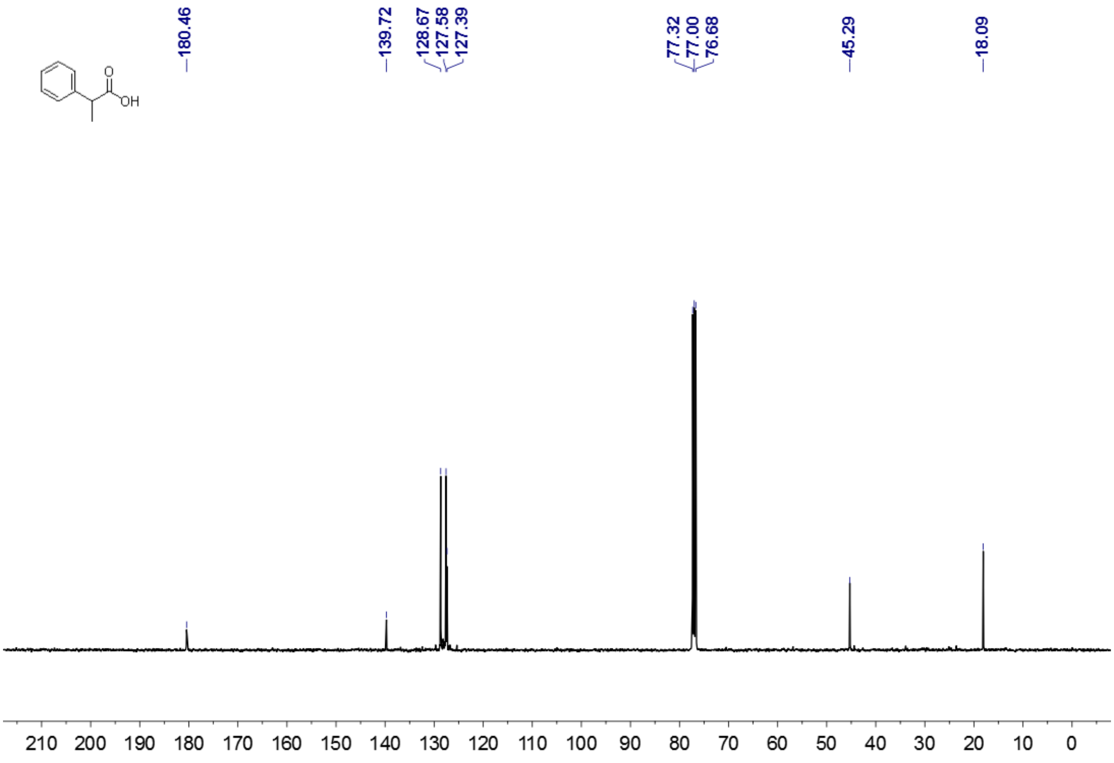

1,2-Dihydrosantonin (4a) in $CDCl_3$

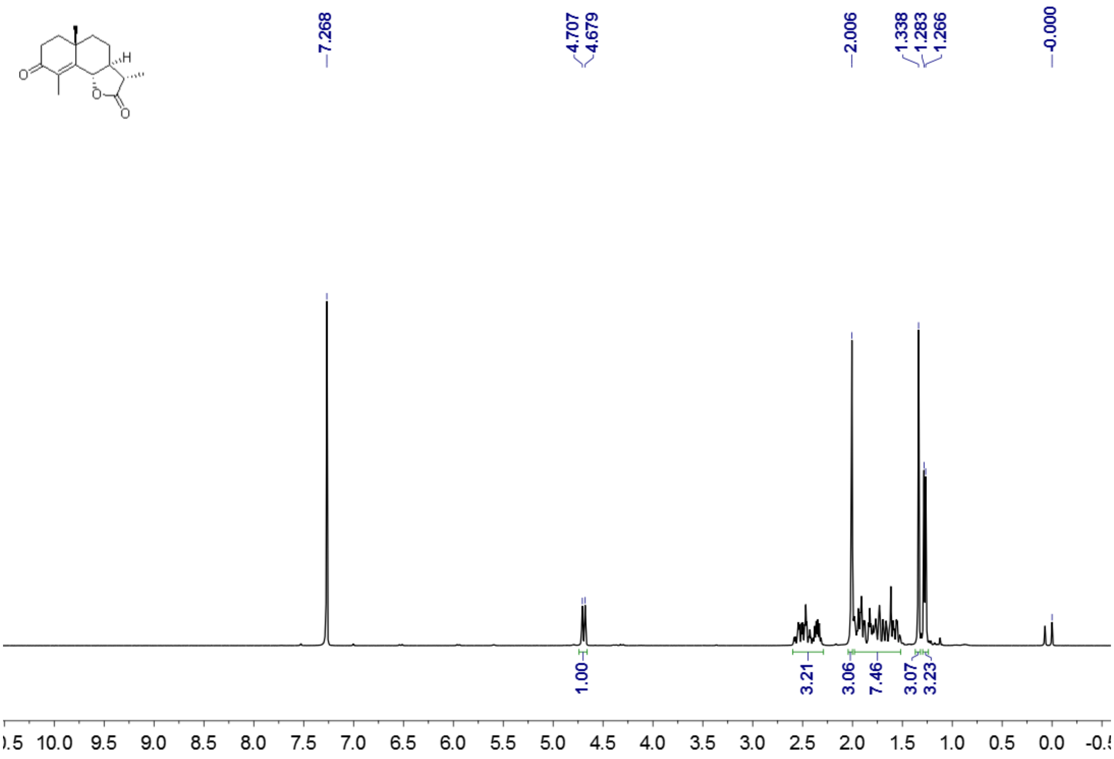

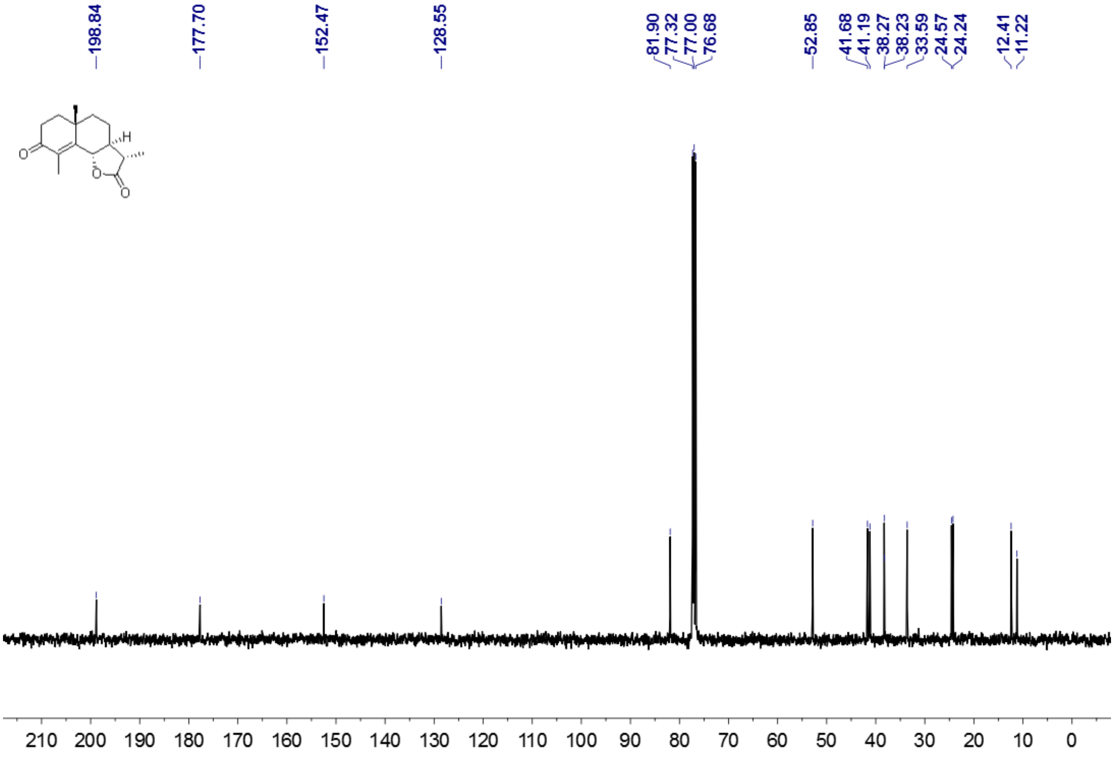

6. Copies of ^{1}H NMR spectra of crude reaction mixtures in Table 1 (optimization of reaction conditions) and Table 2 (substrate scope extension)

6.1 Copies of ^{1}H NMR spectra of crude reaction mixtures in Table 1 (optimization of reaction conditions)

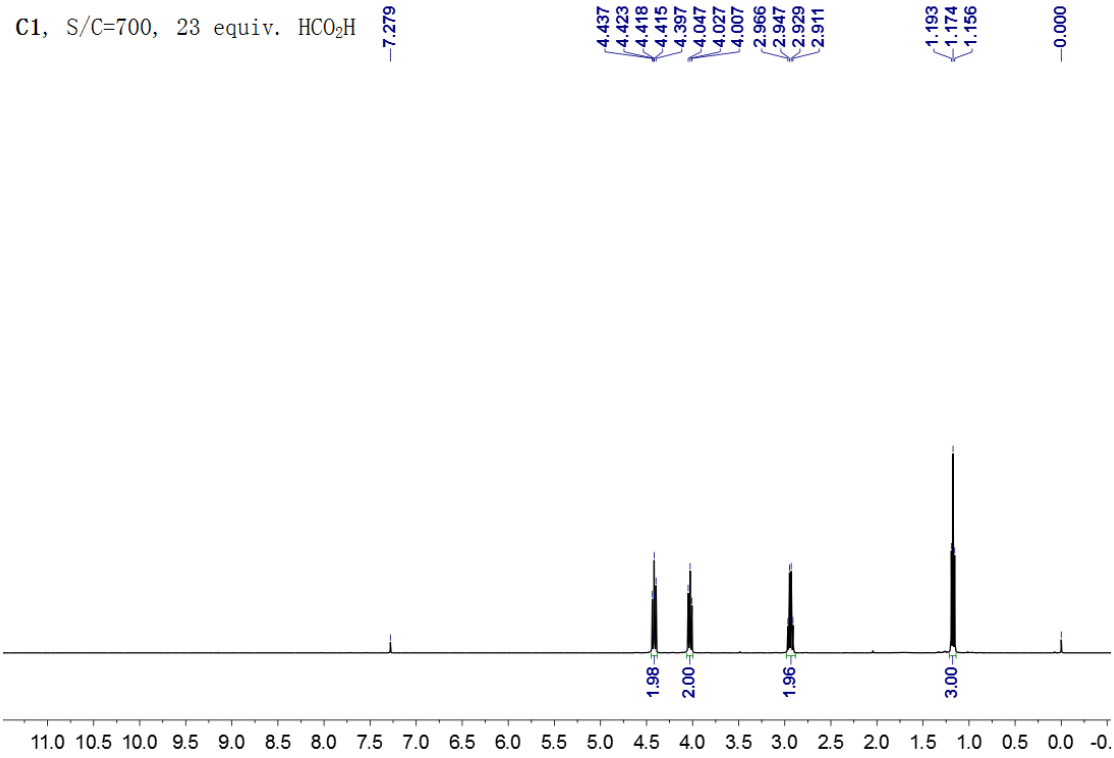

C2, S/C=700, 23 equiv. HCO_2H

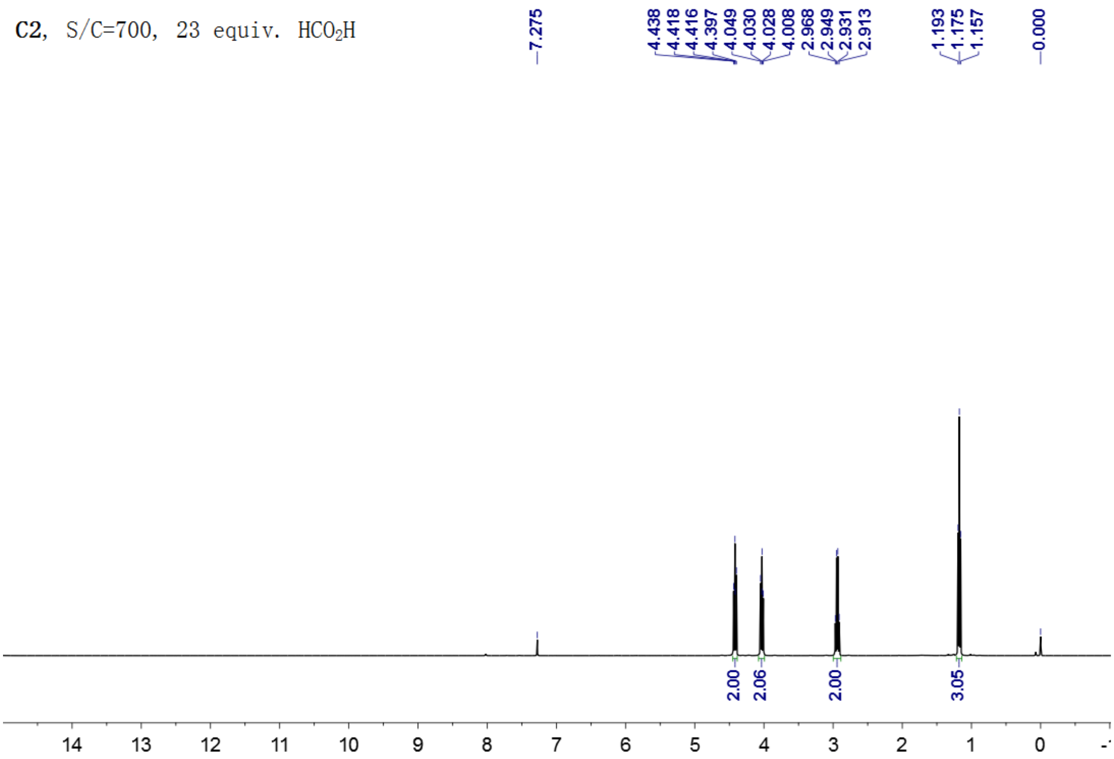

C3, S/C=700, 23 equiv. HCO_2H

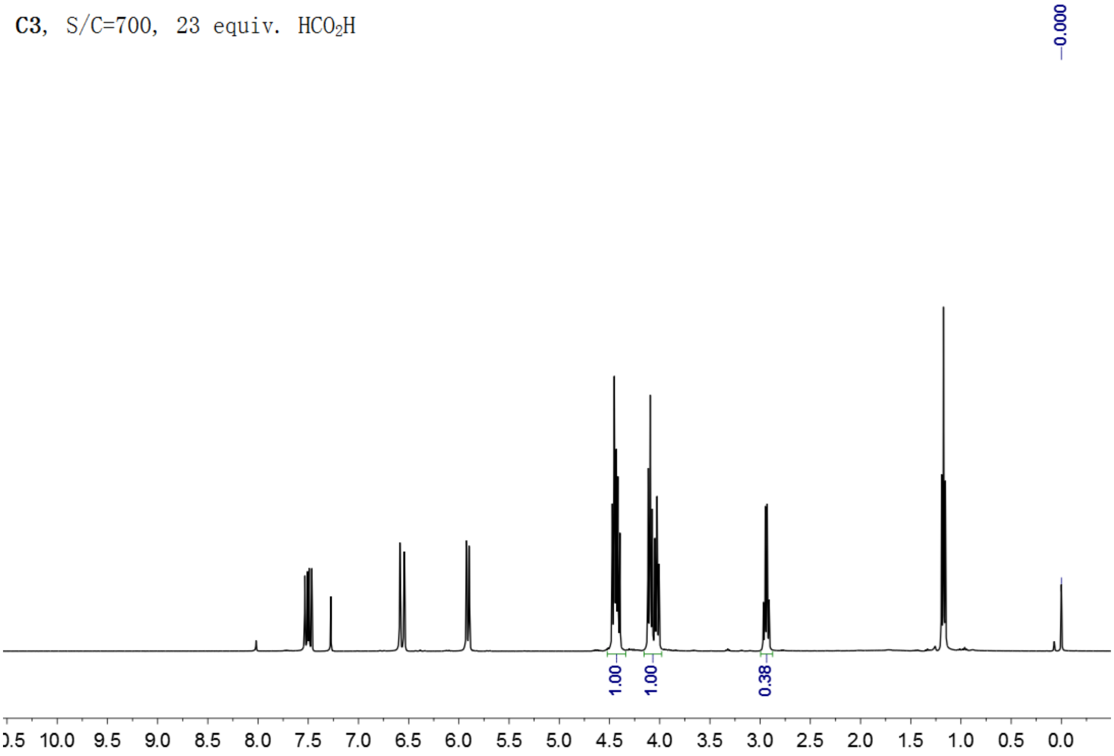

C4, S/C=700, 23 equiv. HCO_2H

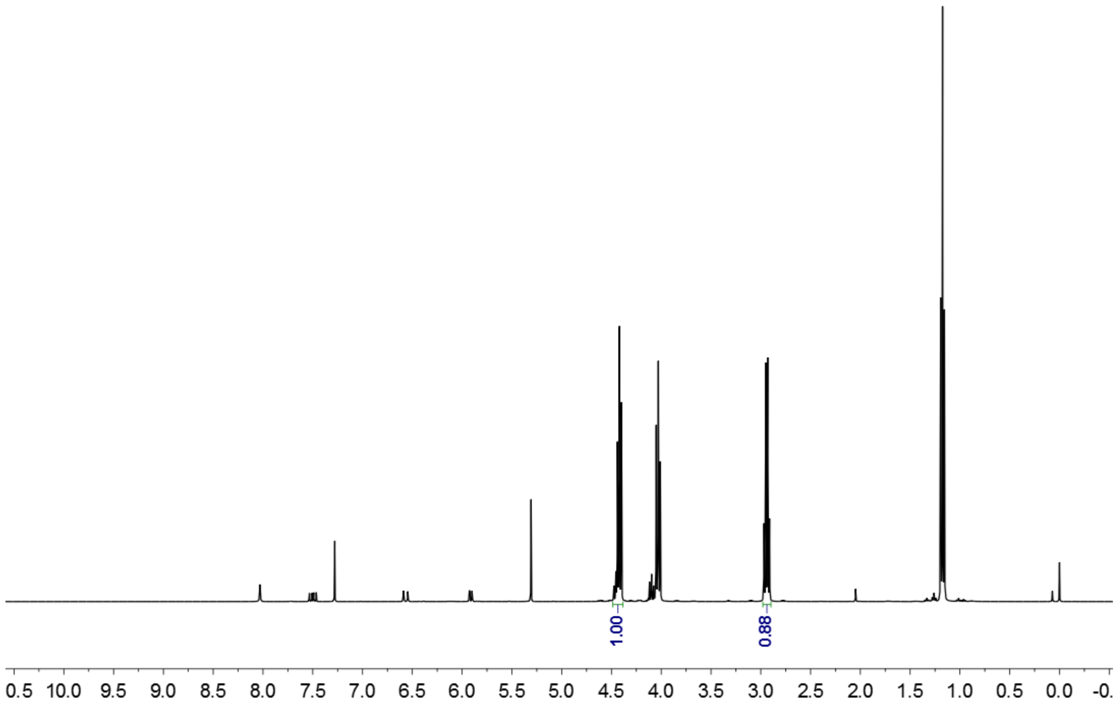

C5, S/C=700, 23 equiv. HCO_2H

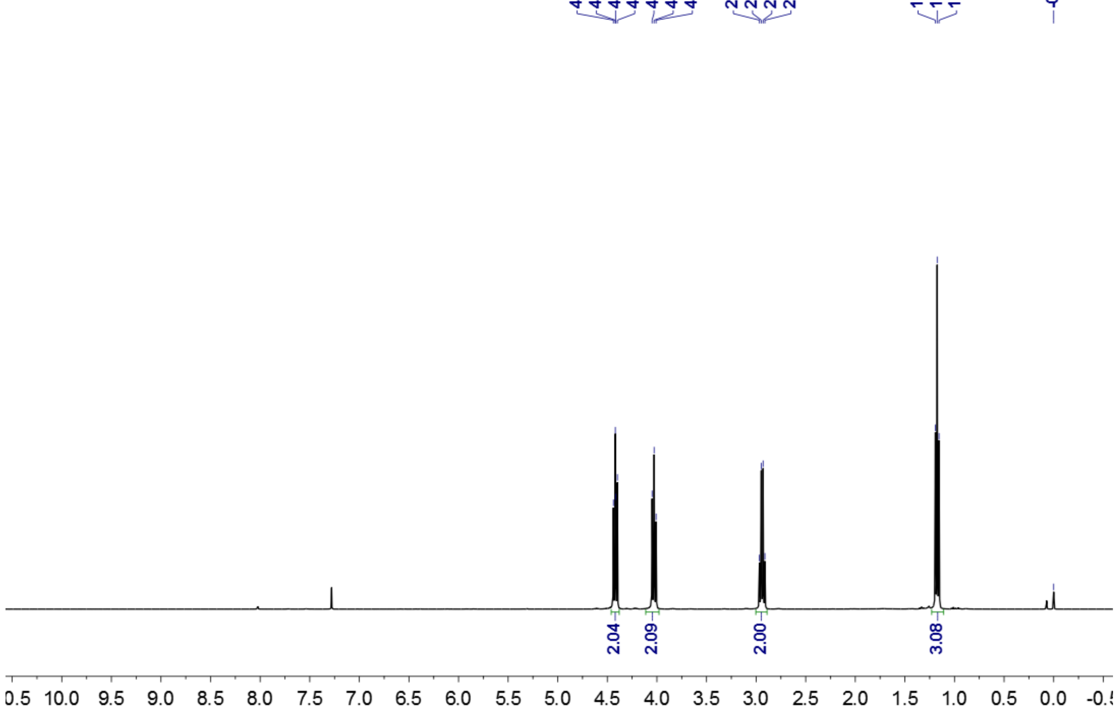

C6, S/C=700, 23 equiv. HCO_2H

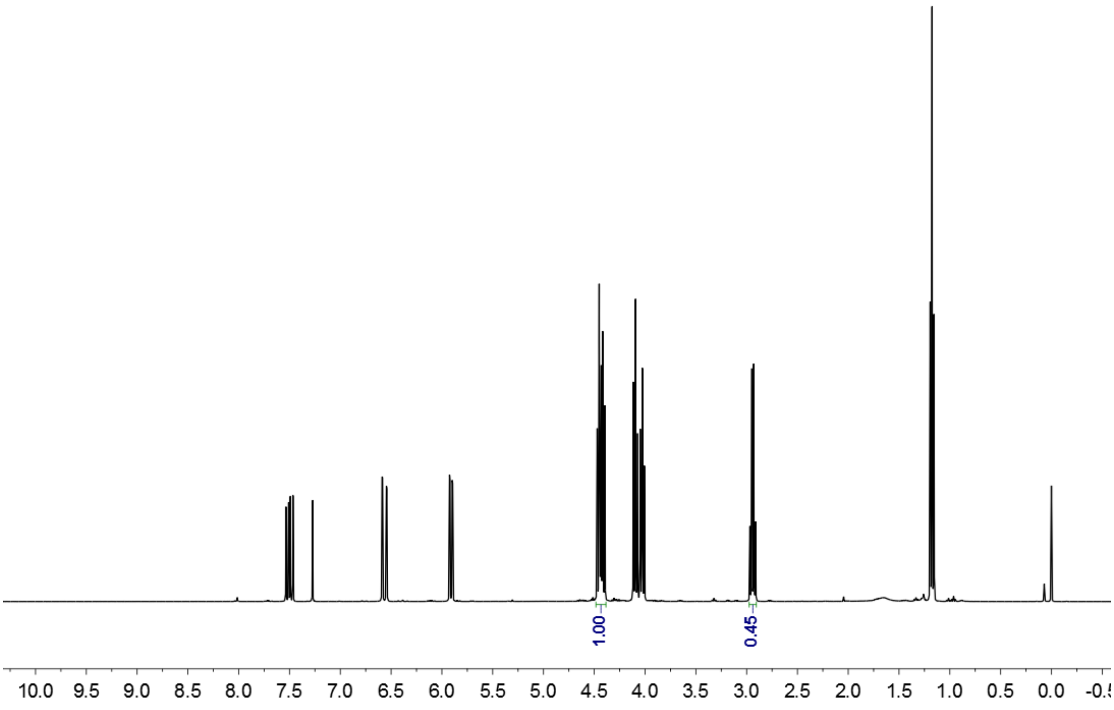

C7, S/C=700, 23 equiv. HCO_2H

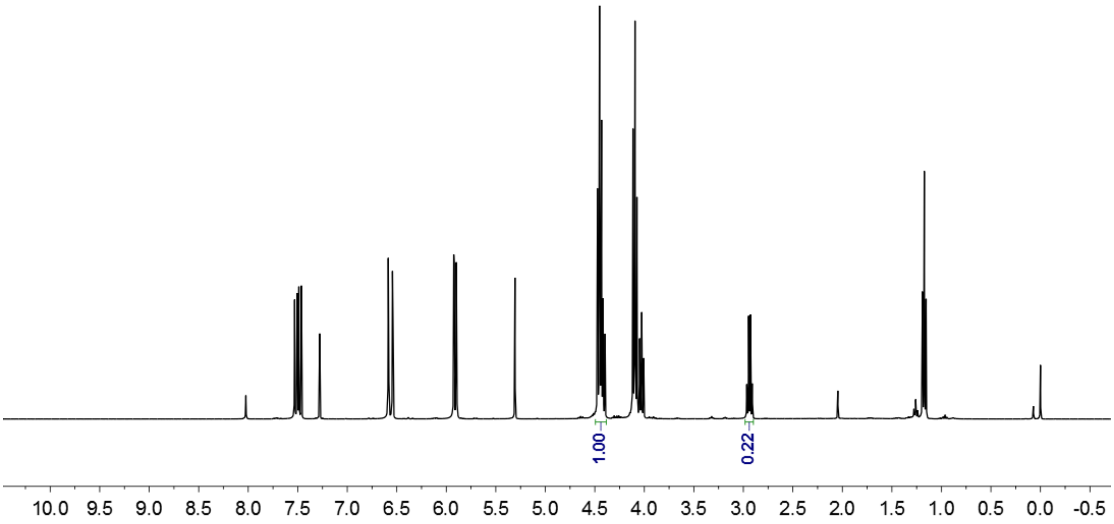

C8, S/C=700, 23 equiv. HCO_2H

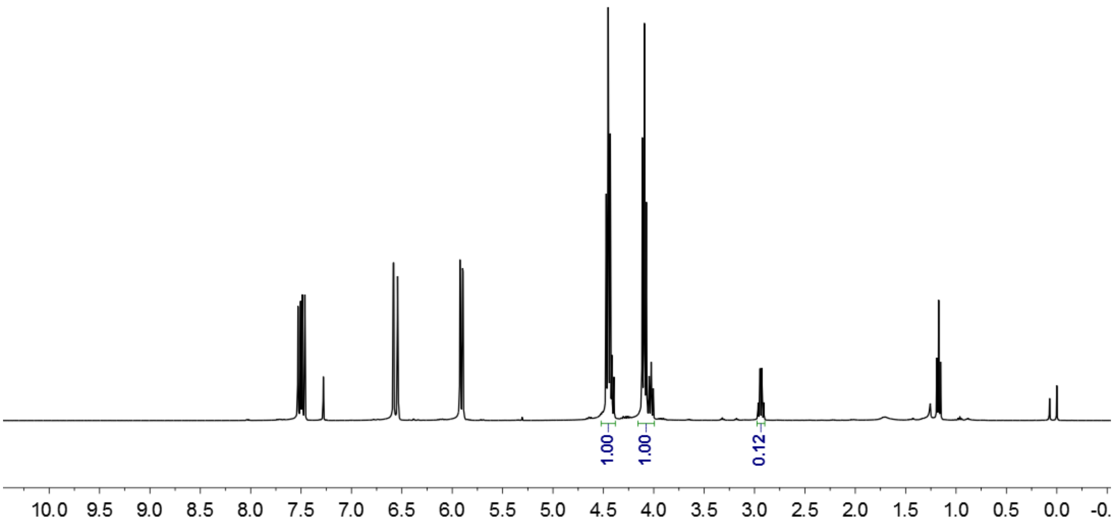

C9, S/C=700, 23 equiv. HCO_2H

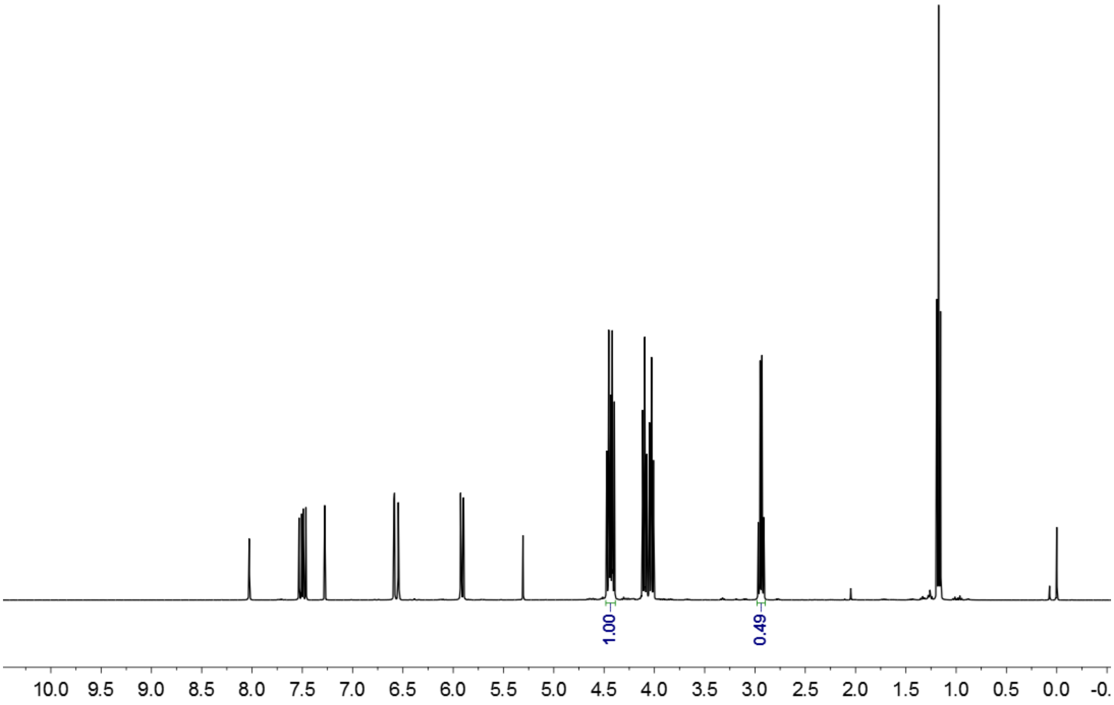

C10, S/C=700, 23 equiv. HCO_2H

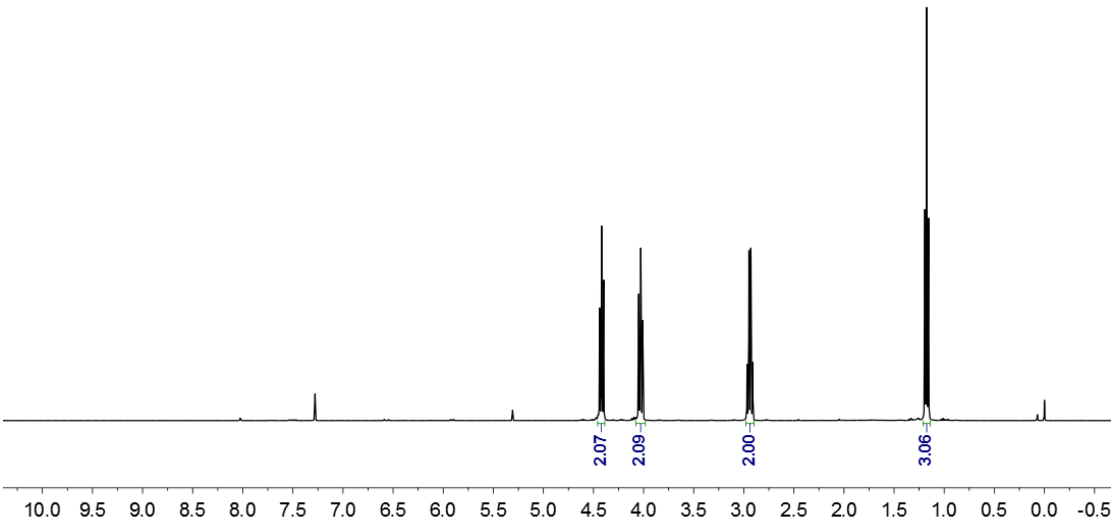

C11, S/C=700, 23 equiv. HCO_2H

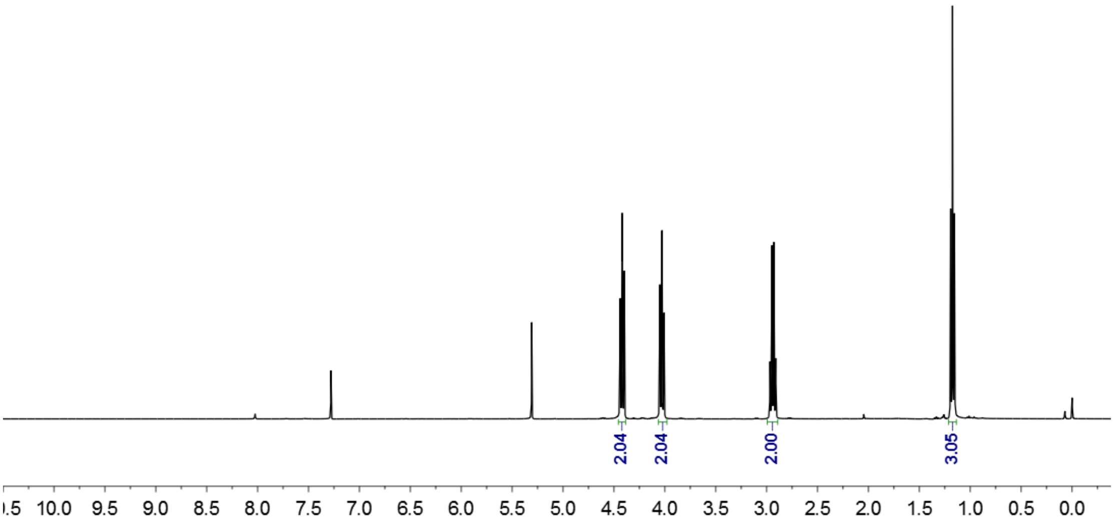

C1, S/C=1400, 23 equiv. HCO_2H

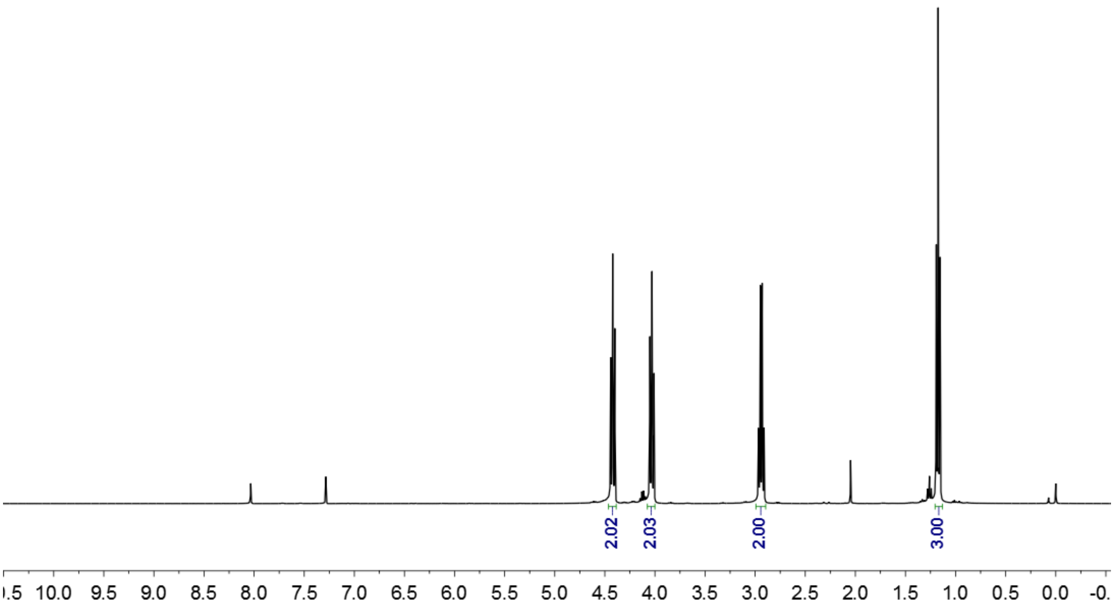

C2, S/C=1400, 23 equiv. HCO_2H

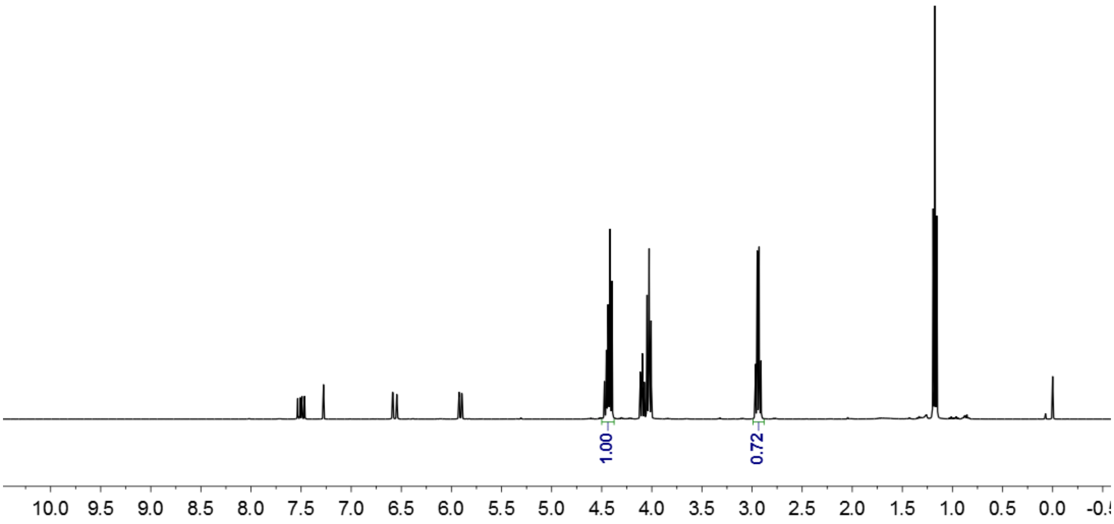

C5, S/C=1400, 23 equiv. HCO_2H

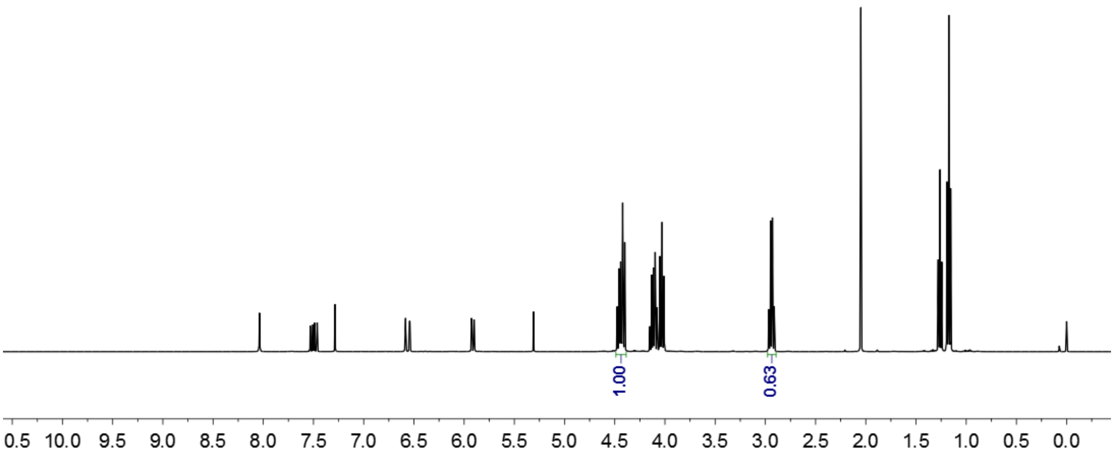

C10, S/C=1400, 23 equiv. HCO_2H

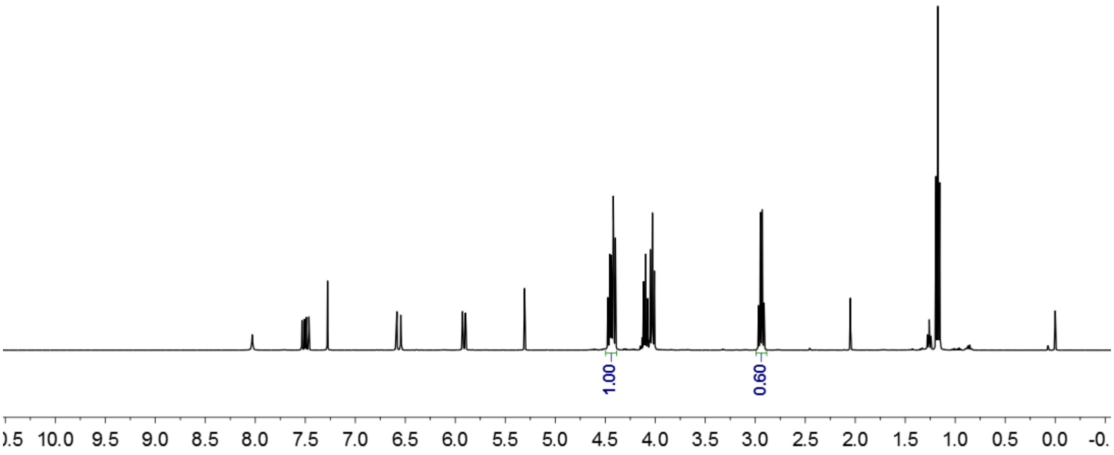

C11, S/C=1400, 23 equiv. HCO_2H

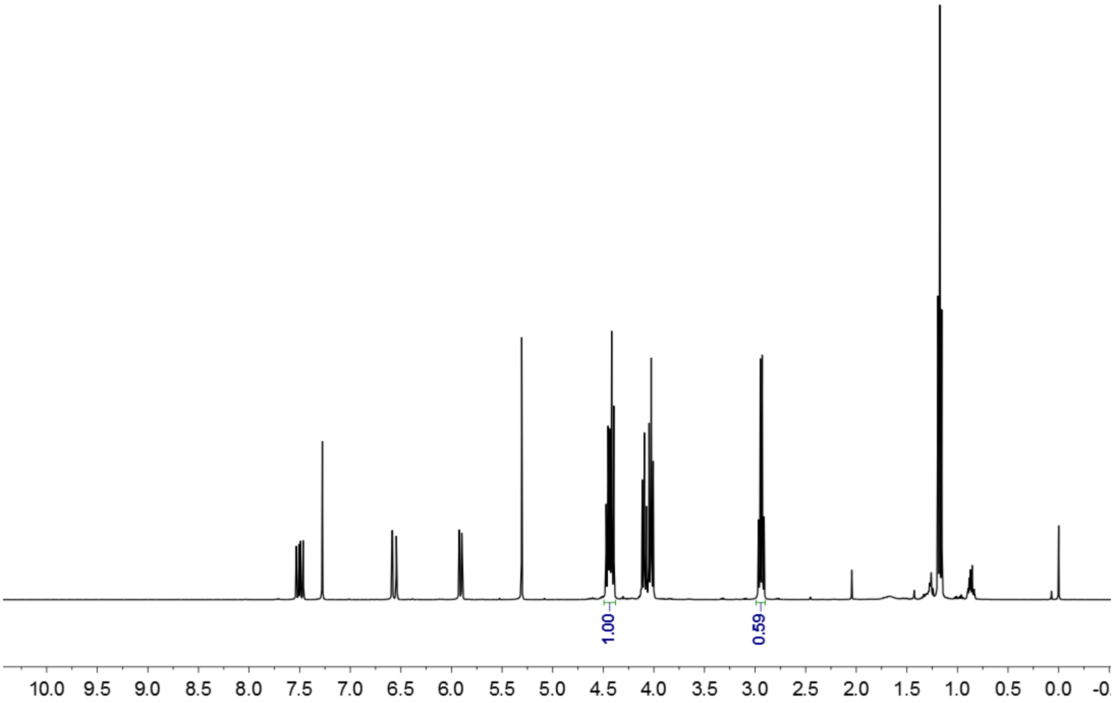

C1, S/C=3500, 23 equiv. HCO_2H

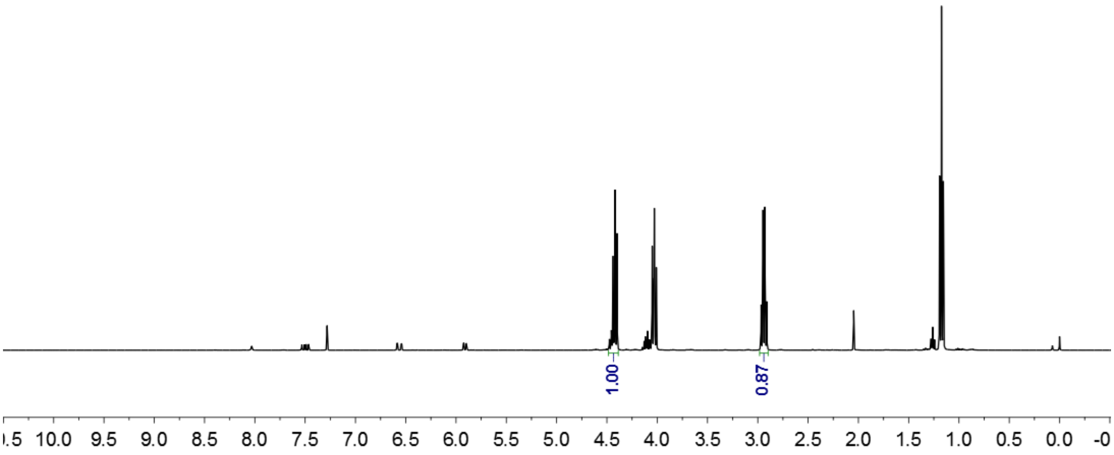

C1, S/C=7000, 23 equiv. HCO_2H

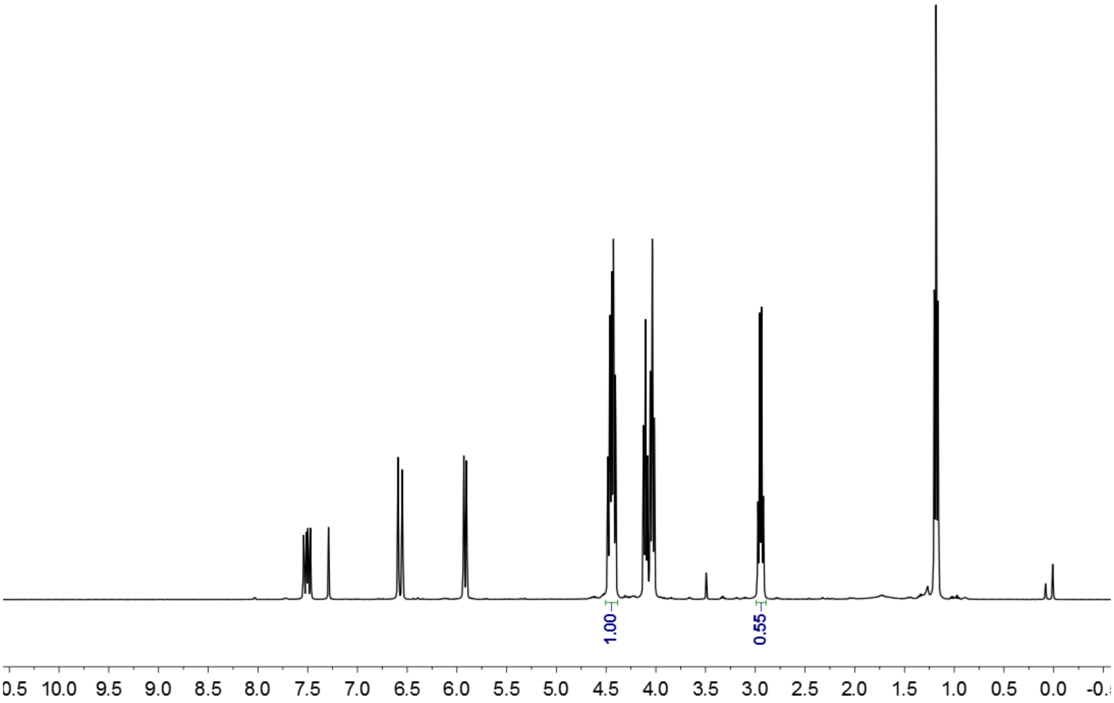

C1, S/C=1400, 3 equiv. HCO_2H

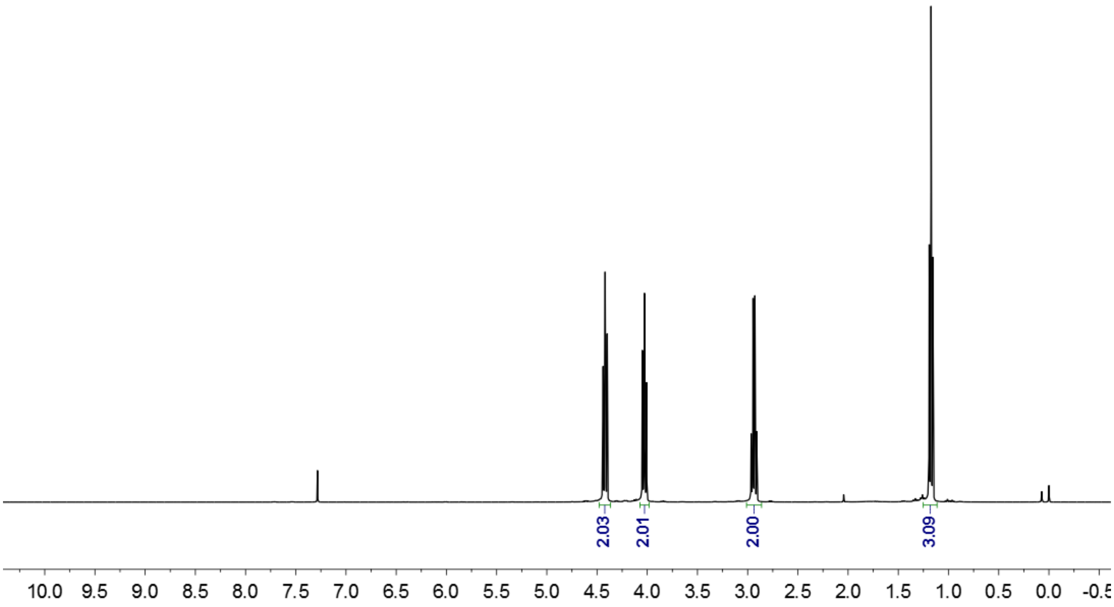

C1, S/C=1400, 6 equiv. HCO_2H

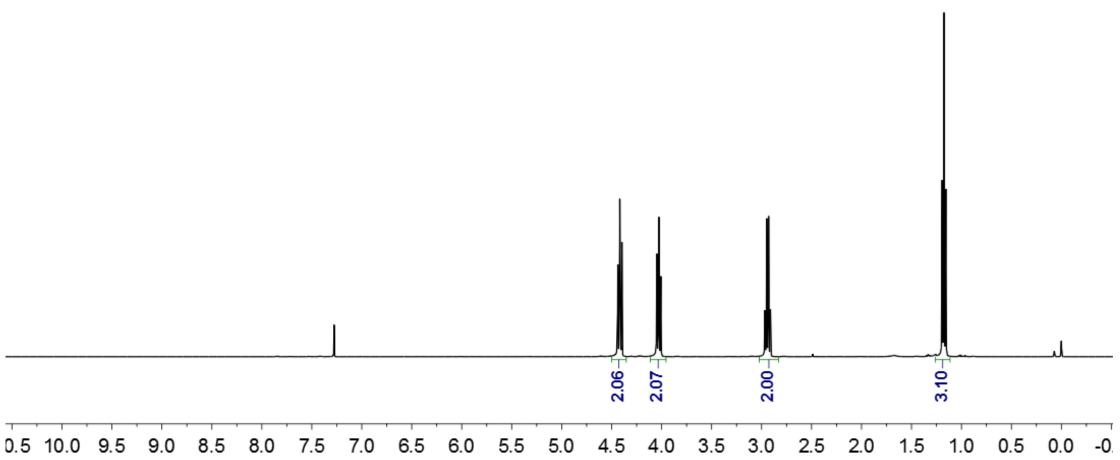

C1, S/C=1400, 11 equiv. HCO_2H

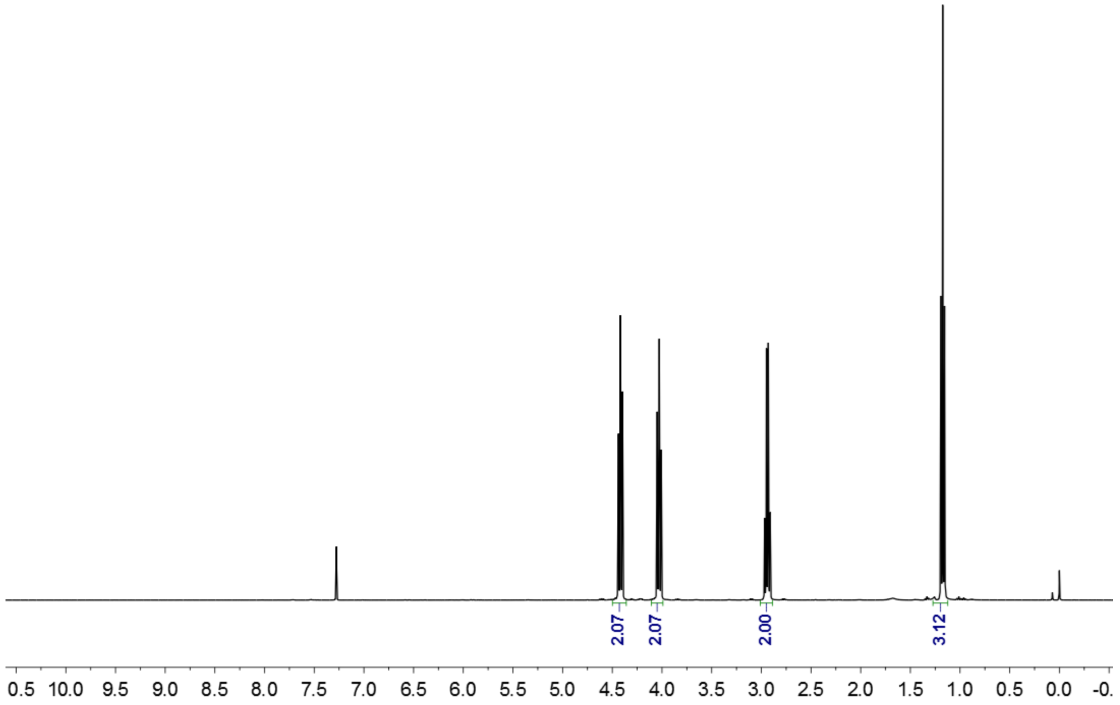

C1, S/C=1400, 17 equiv. HCO_2H

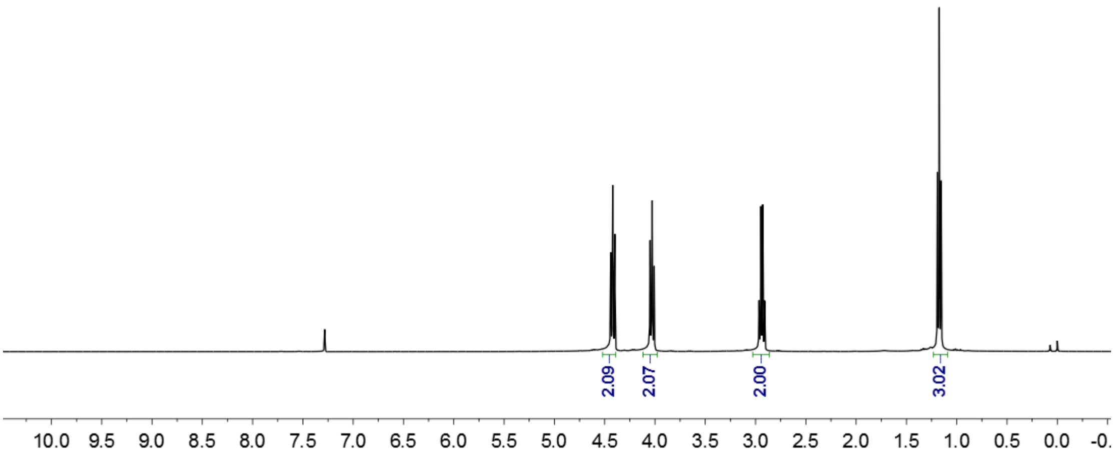

C1, S/C=7000, 23 equiv. HCO_2H, 5 min

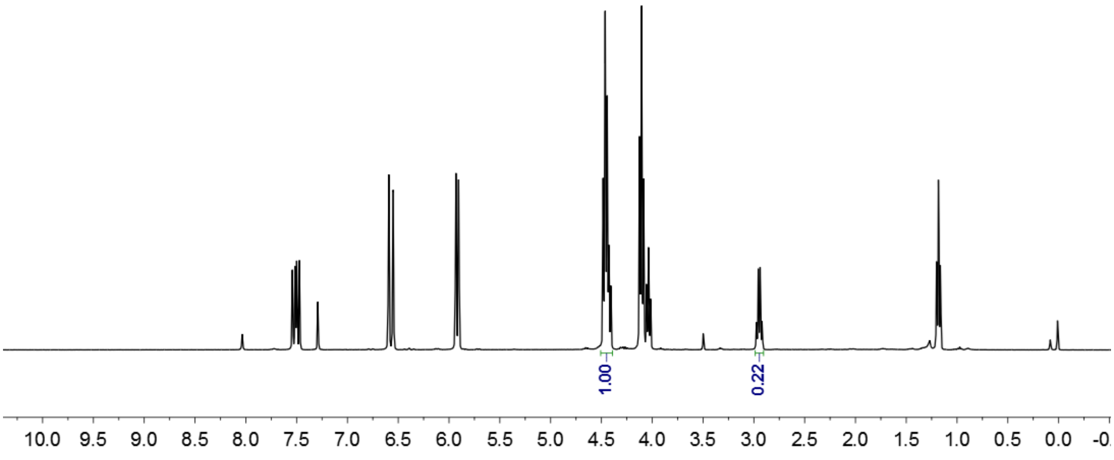

C1, S/C=7000, 23 equiv. HCO_2H, 10 min

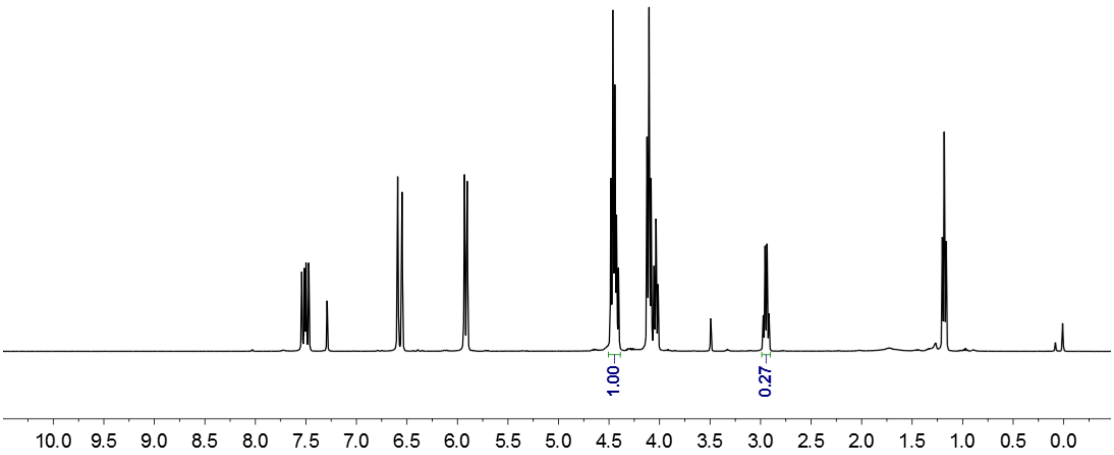

C1, S/C=7000, 23 equiv. HCO_2H, 30 min

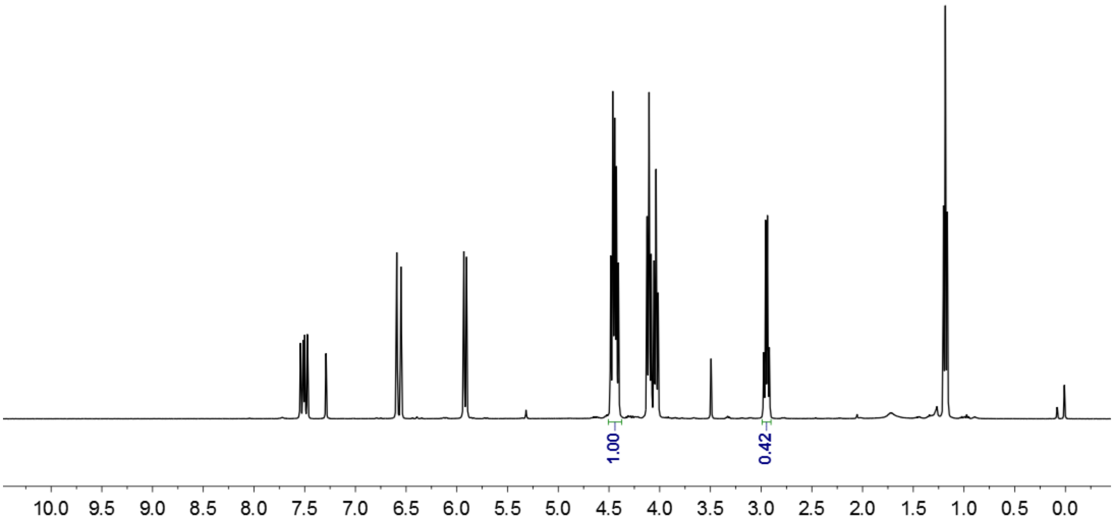

C1, S/C=7000, 23 equiv. HCO_2H, 120 min

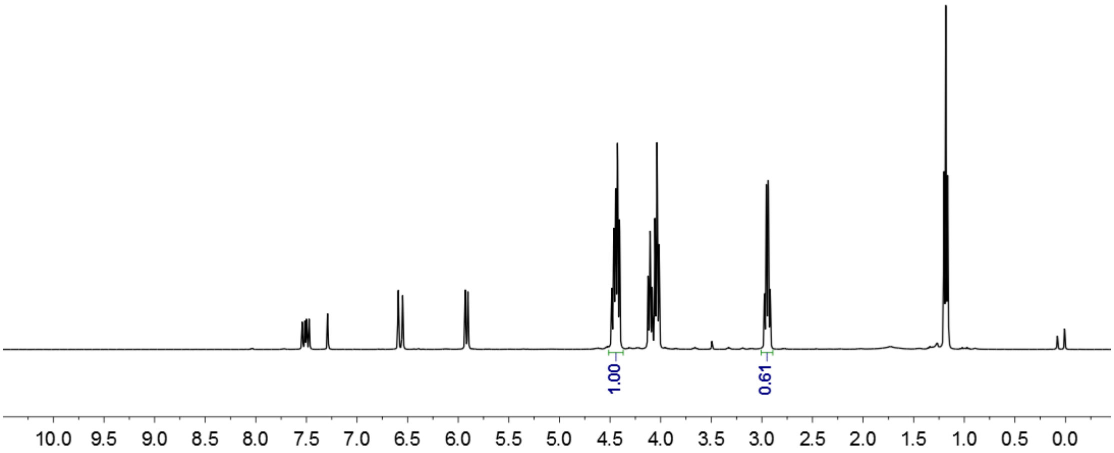

6.2 Copies of ¹H NMR spectra of crude reaction mixtures in Table 2 (substrate scope extension)

Propionamide (2b) (in *Reaction Conditions B*)

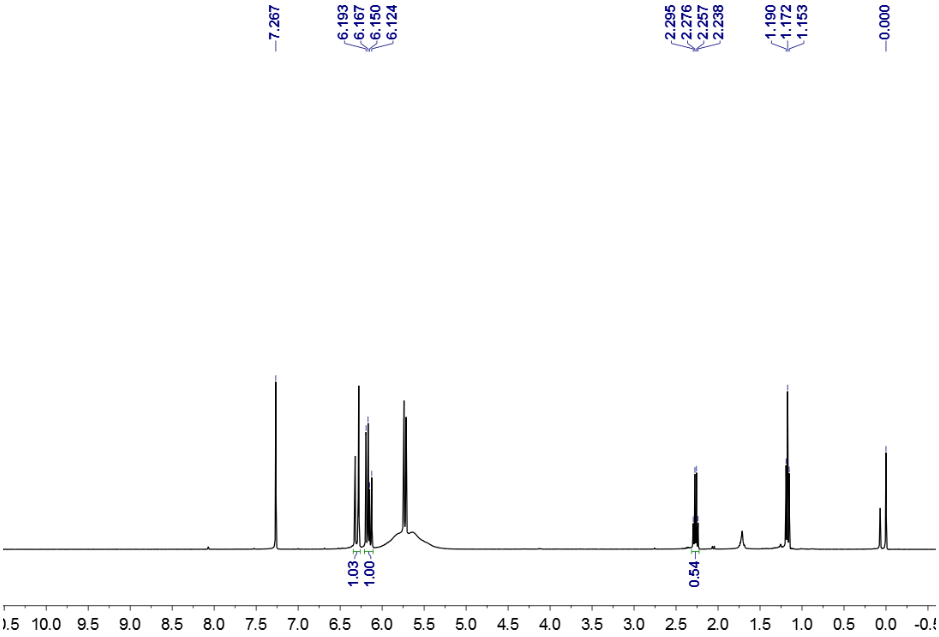

Propionamide (2b) (in *Reaction Conditions D*)

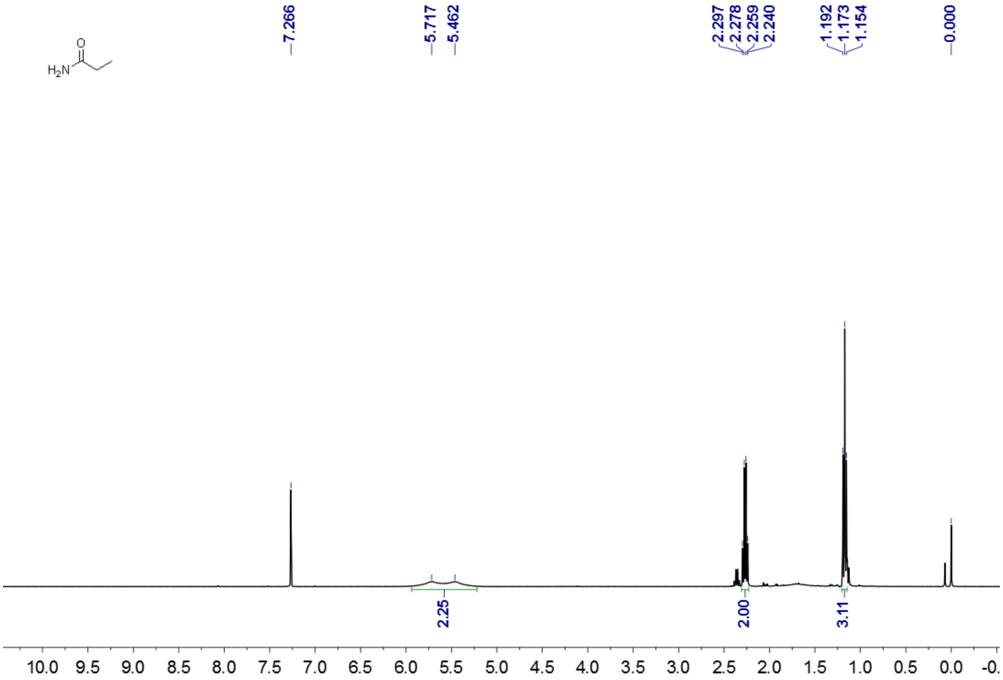

***N,N*-Diethylpropionamide (2c)** (in *Reaction Conditions C*)

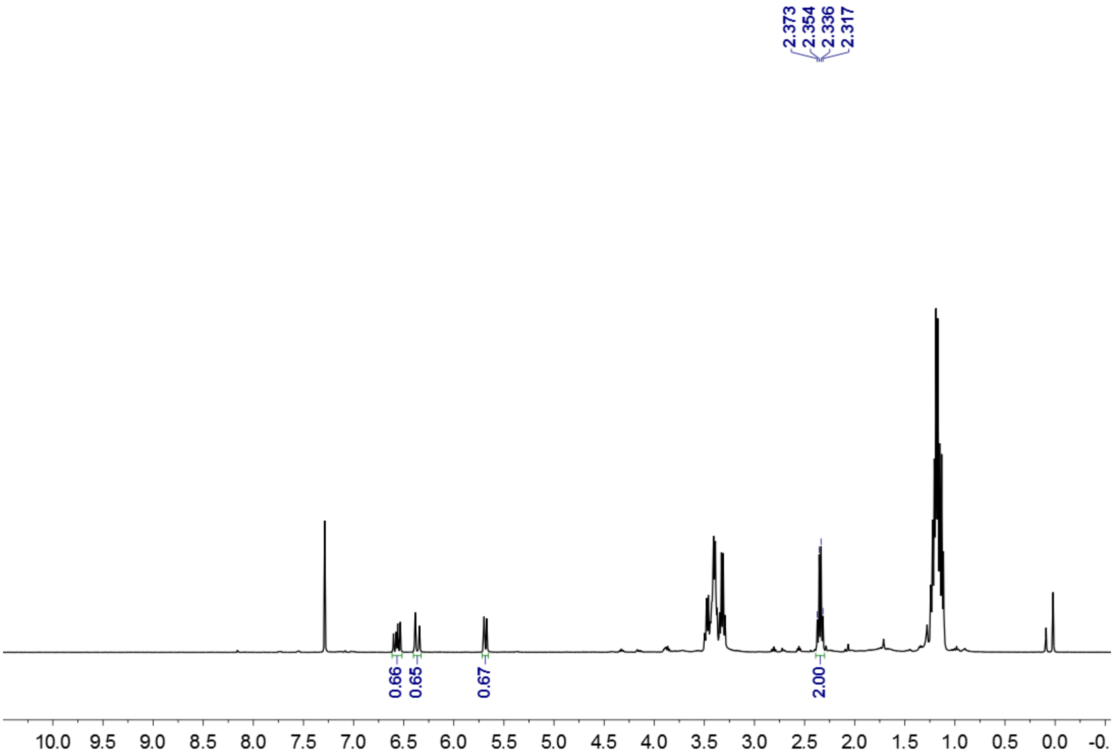

***N,N*-Diethylpropionamide (2c)** (in *Reaction Conditions D*)

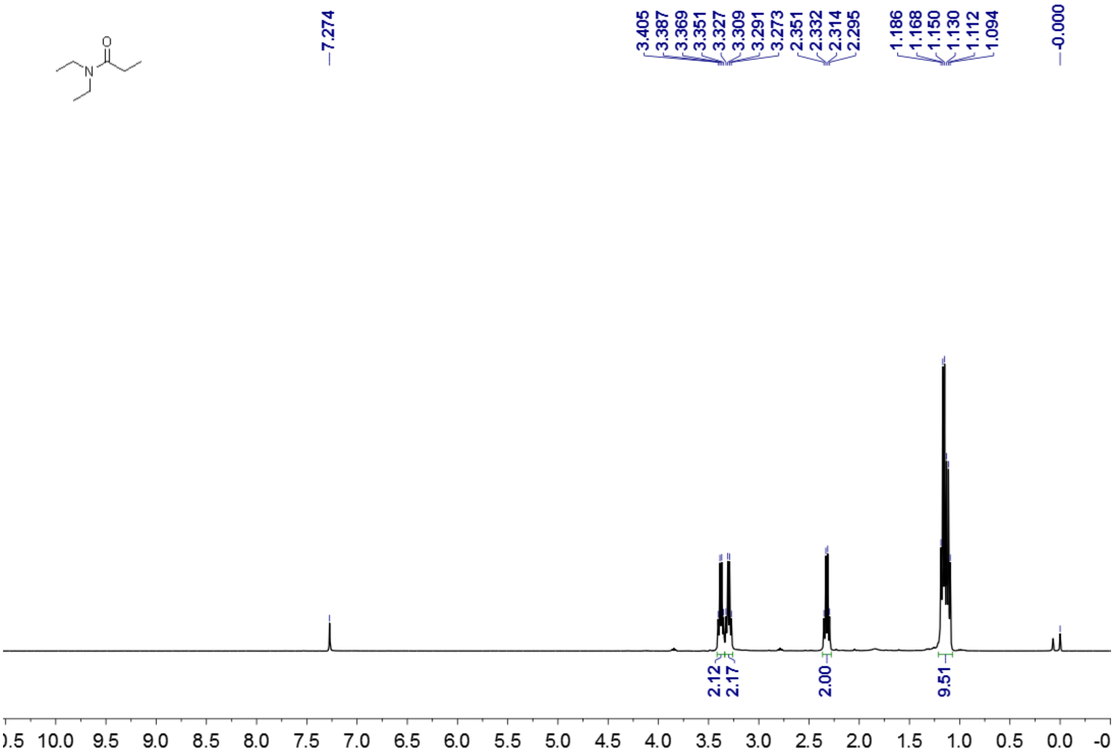

***N*-Propionylpyrrolidine (2d)** (in *Reaction Conditions C*)

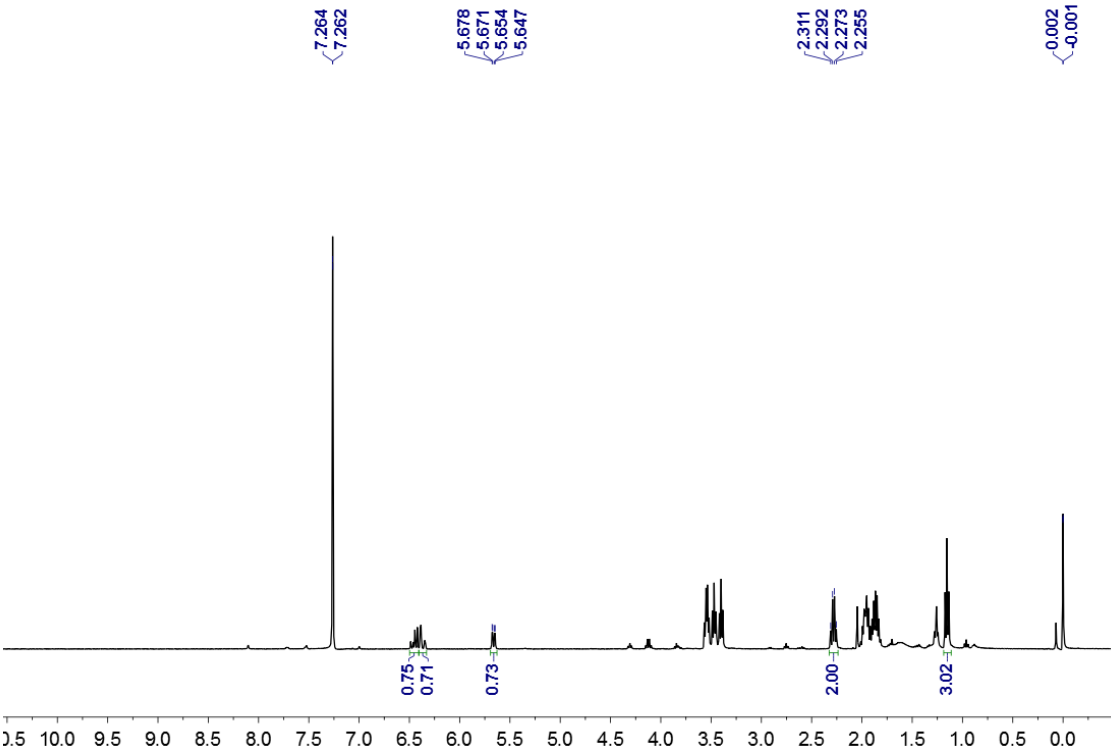

***N*-Propionylpyrrolidine (2d)** (in *Reaction Conditions D*)

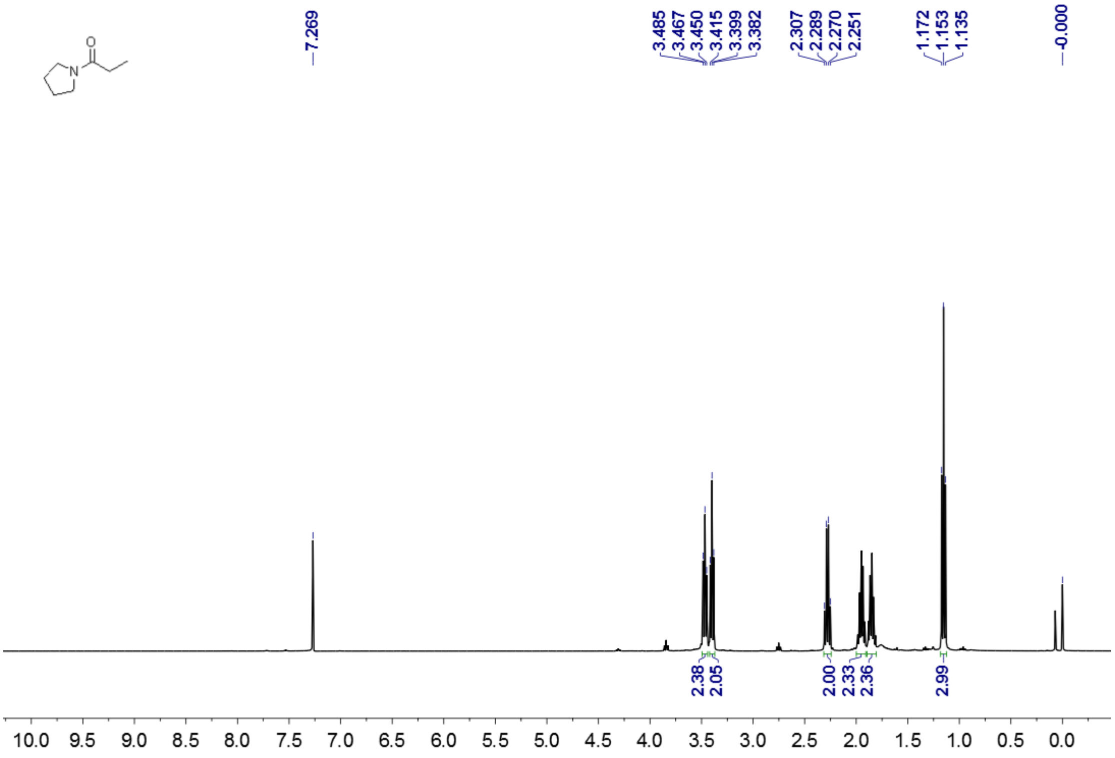

***N,N*-Dibenzylpropionamide (2e)** (in *Reaction Conditions C*)

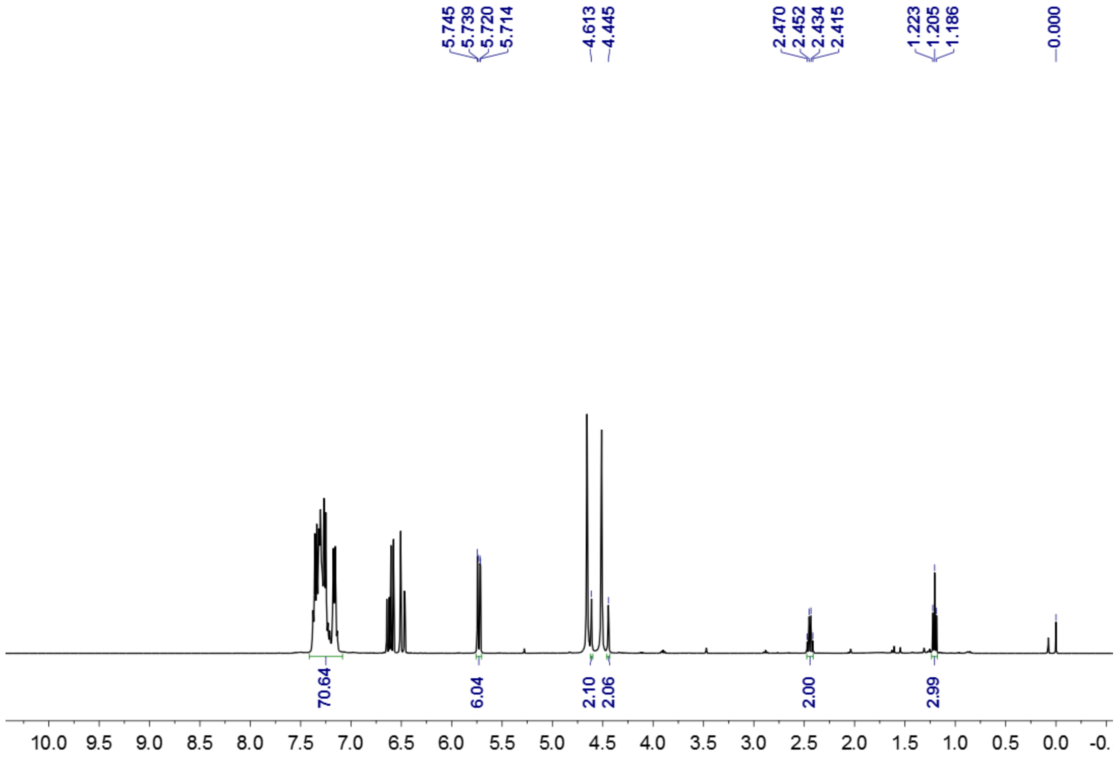

***N,N*-Dibenzylpropionamide (2e)** (in *Reaction Conditions D*)

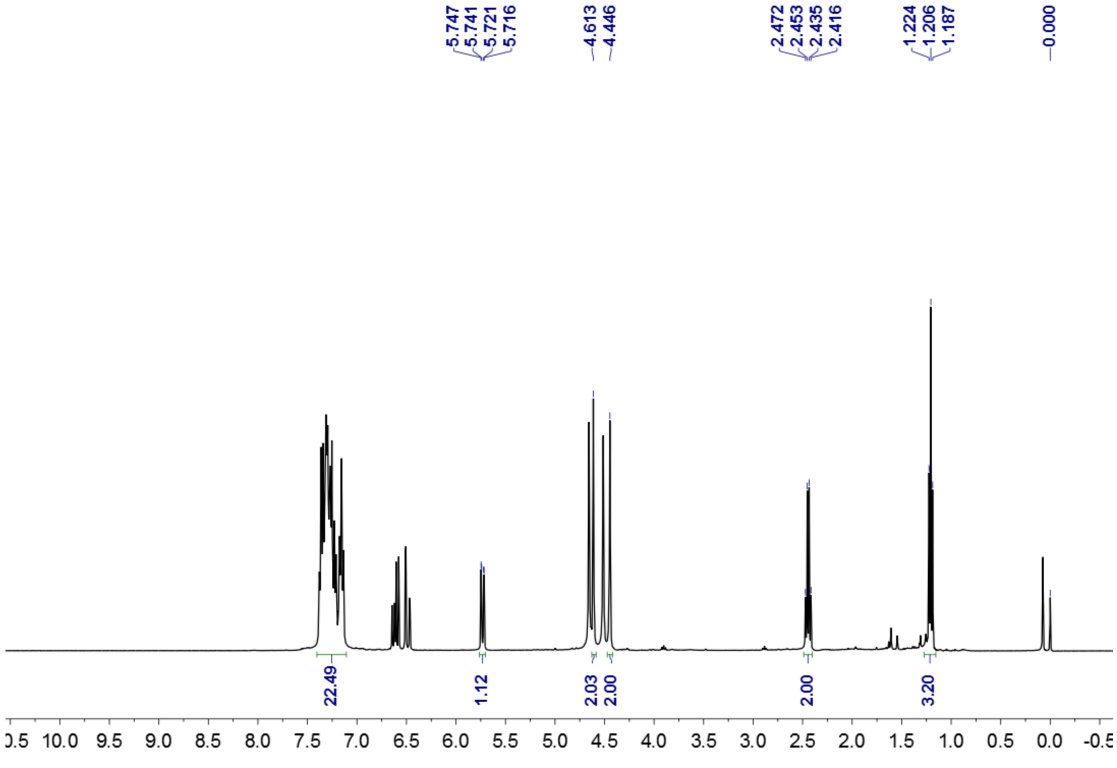

***N*-Methylpropionanilide (2f)** (in *Reaction Conditions C*)

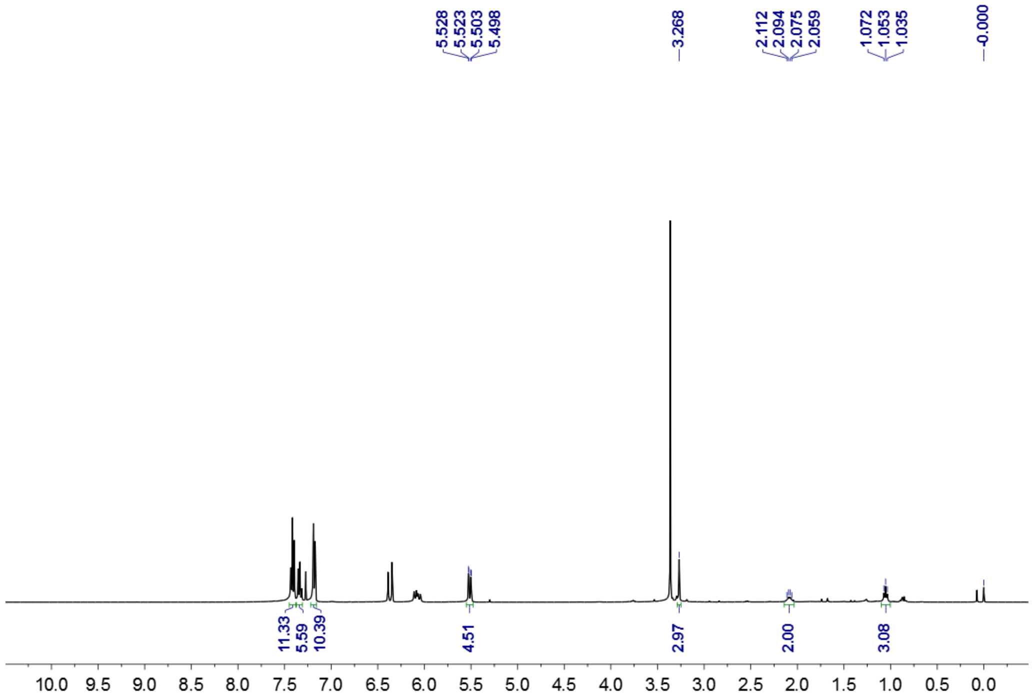

***N*-Methylpropionanilide (2f)** (in *Reaction Conditions D*)

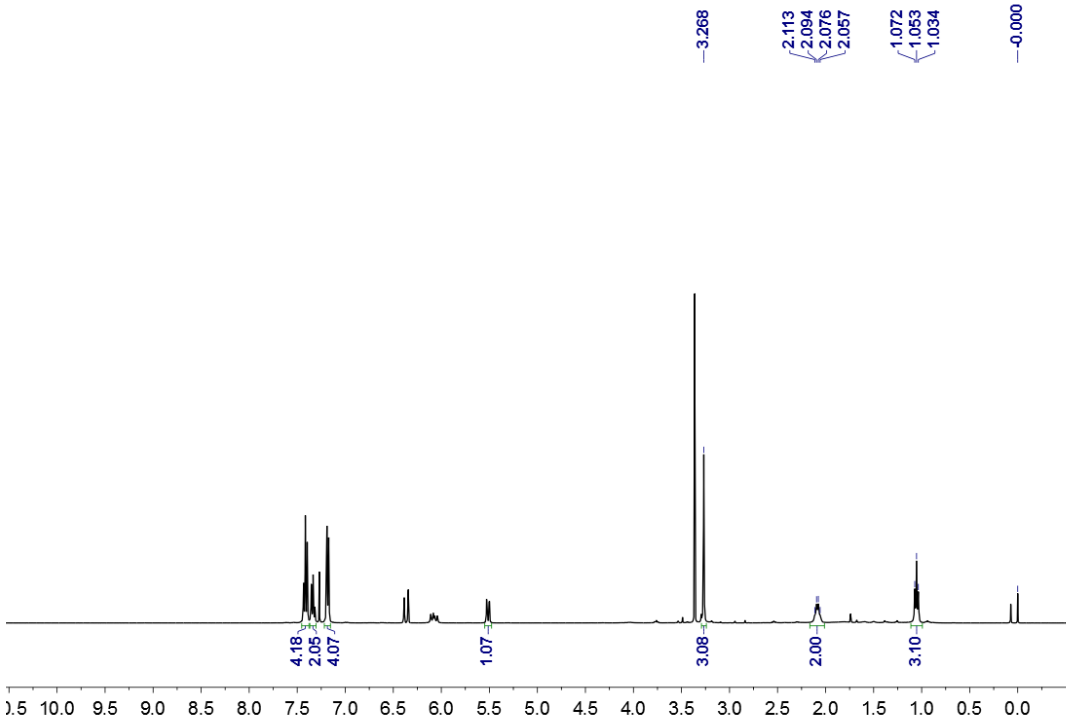

***N,N*-Diphenylpropionamide (2g)** (in *Reaction Conditions C*)

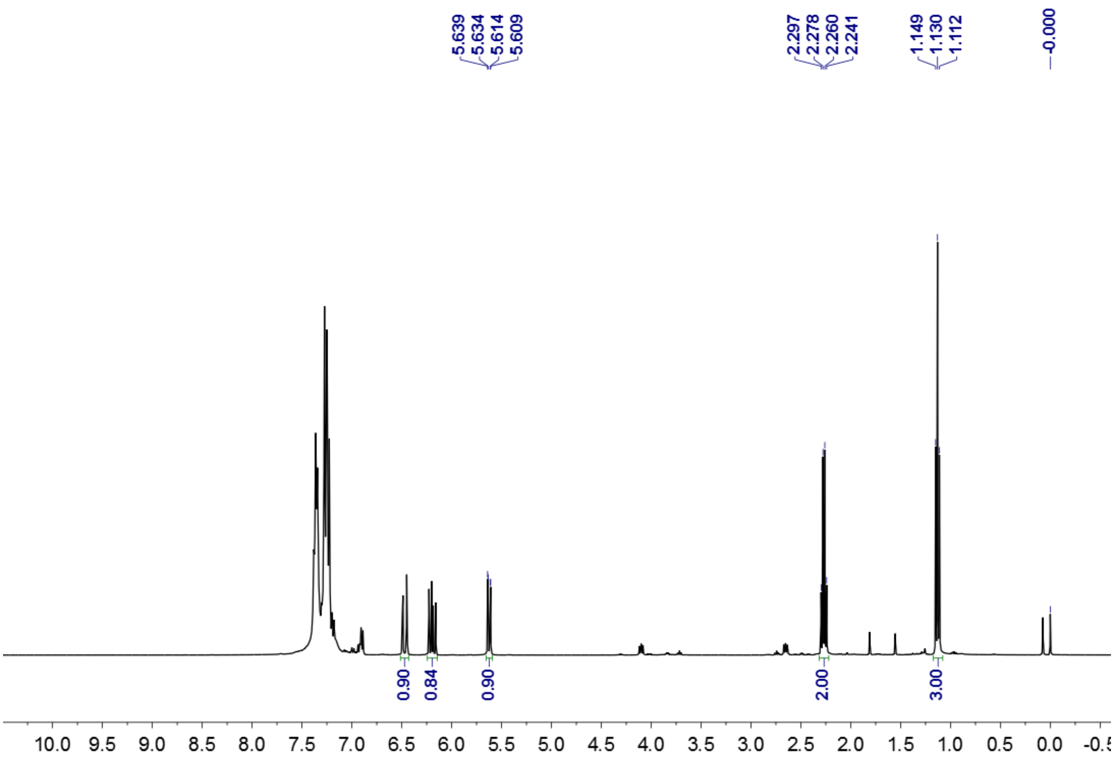

***N,N*-Diphenylpropionamide (2g)** (in *Reaction Conditions D*)

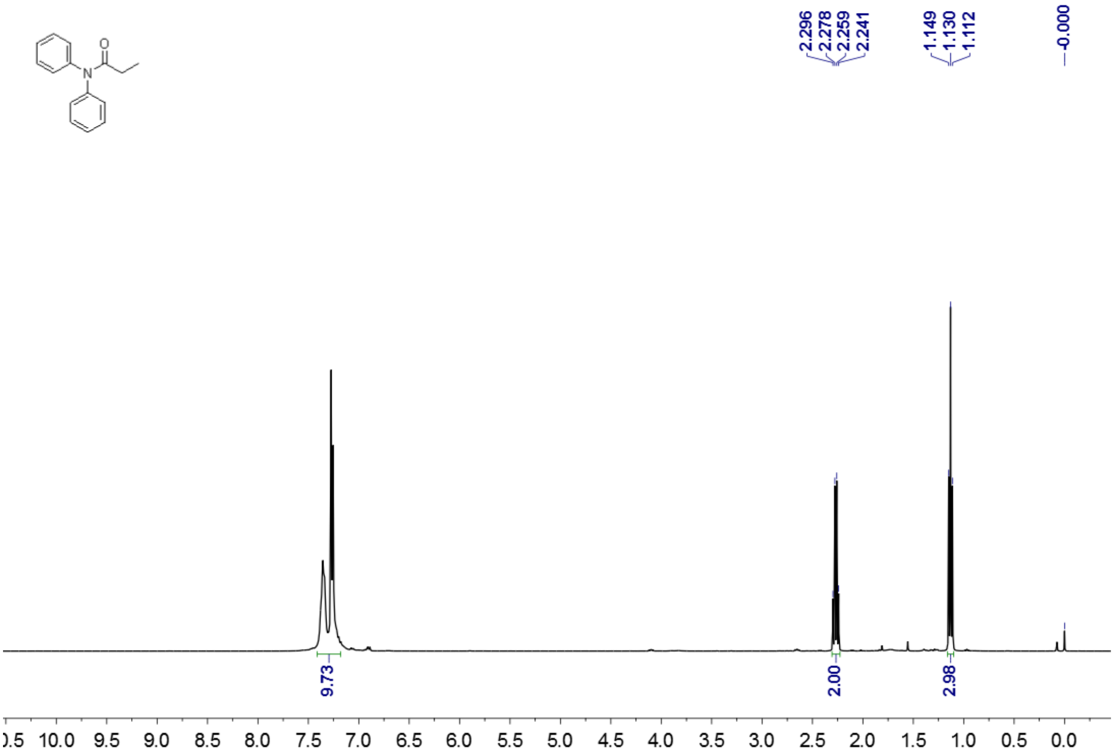

Isobutyramide (2h) (in *Reaction Conditions A*)

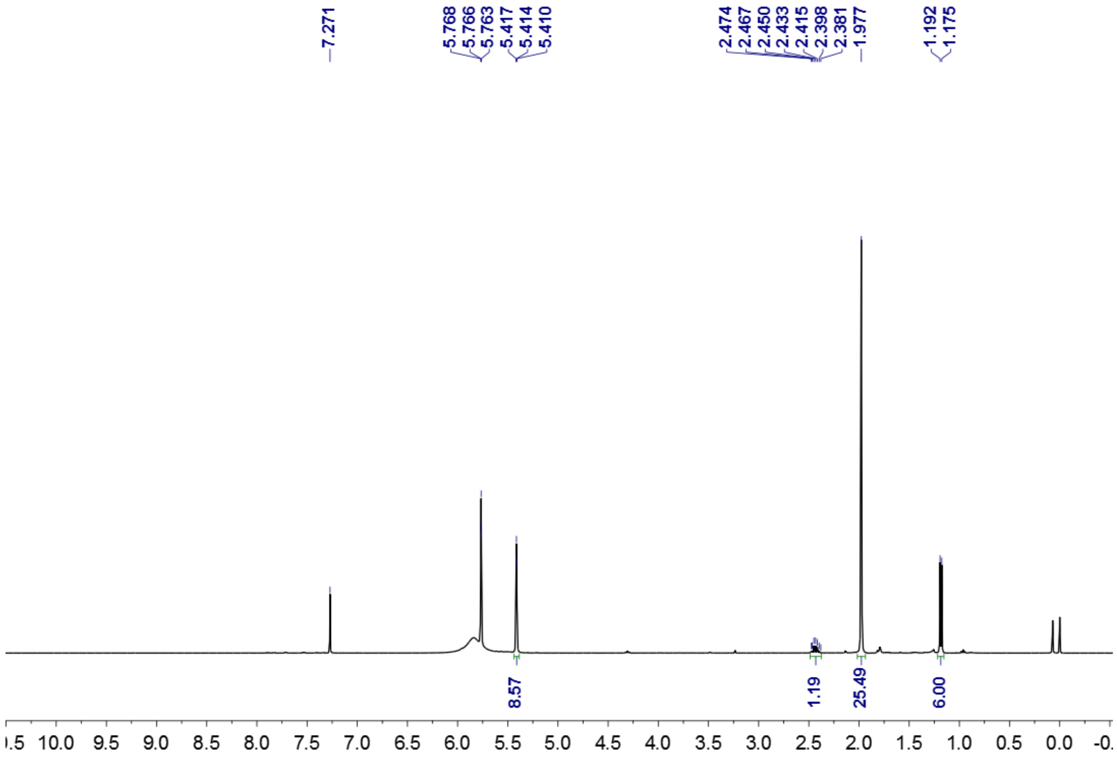

Isobutyramide (2h) (in *Reaction Conditions D*)

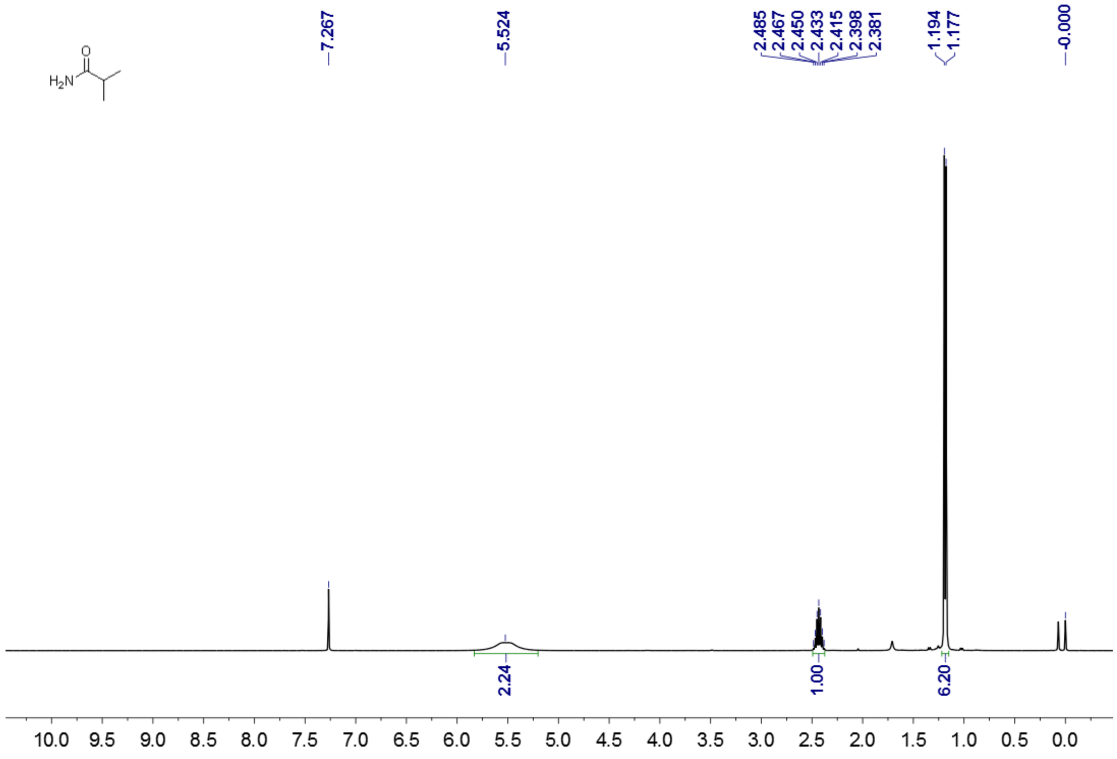

H_2N
O
7.267
5.524
2.485
2.467
2.450
2.433
2.415
2.398
2.381
1.194
1.177
-0.000
2.24
1.00
6.20
10.0 9.5 9.0 8.5 8.0 7.5 7.0 6.5 6.0 5.5 5.0 4.5 4.0 3.5 3.0 2.5 2.0 1.5 1.0 0.5 0.0

3-phenylpropanamide (2i) (in *Reaction Conditions A*)

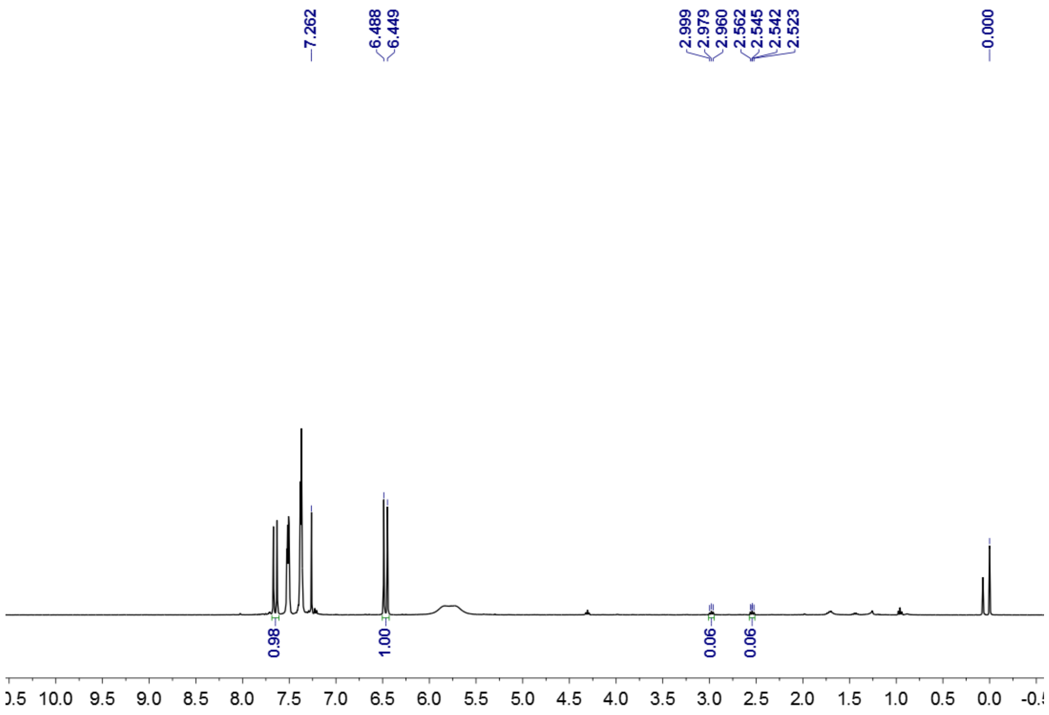

3-phenylpropanamide (2i) (in *Reaction Conditions D*)

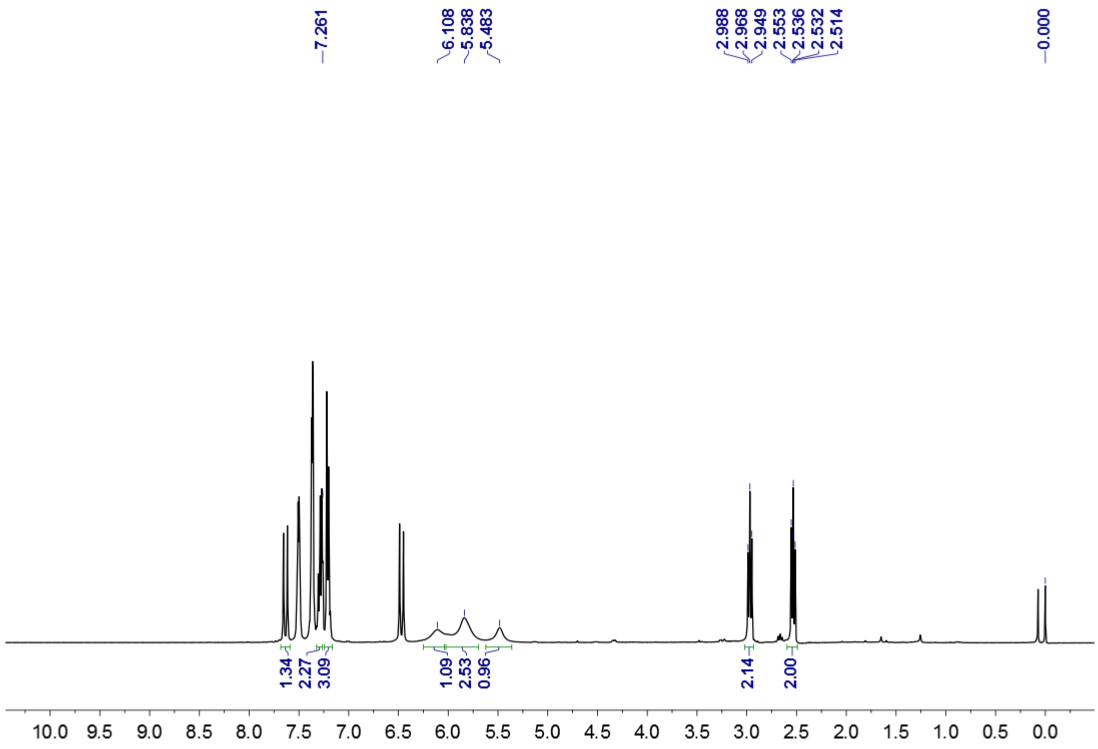

Pyrrolidine-2,5-dione (2j) (in *Reaction Conditions B*)

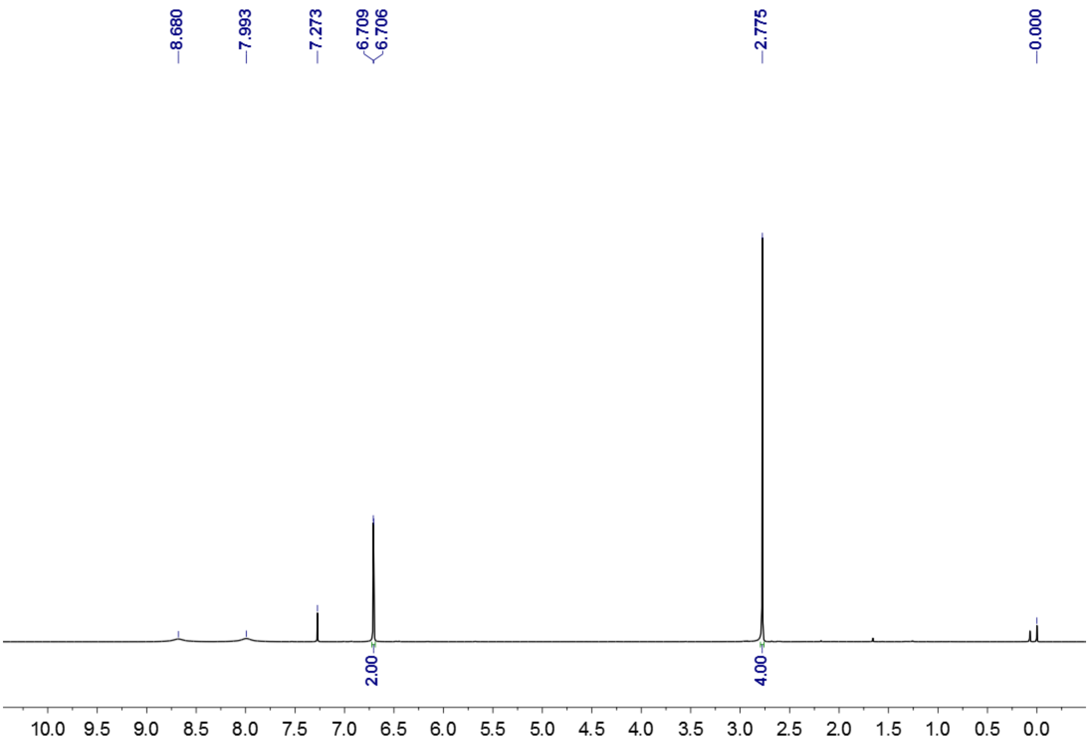

Pyrrolidine-2,5-dione (2j) (in *Reaction Conditions D*)

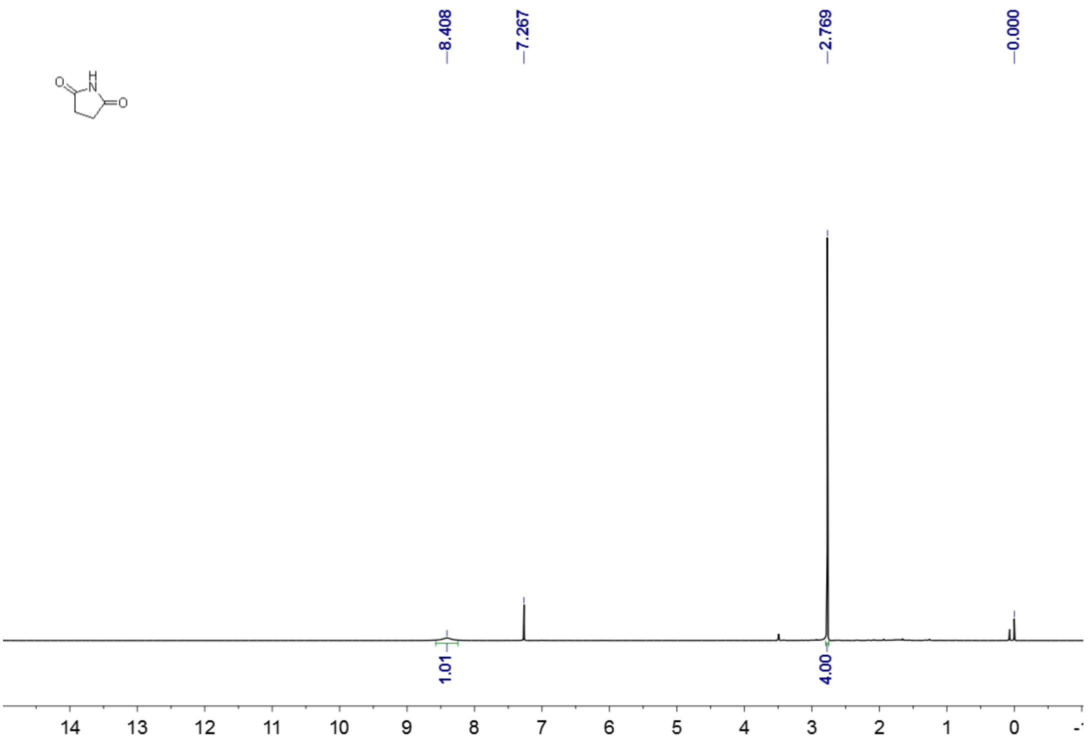

3-phenylpropanoic acid (2k) (in *Reaction Conditions C*)

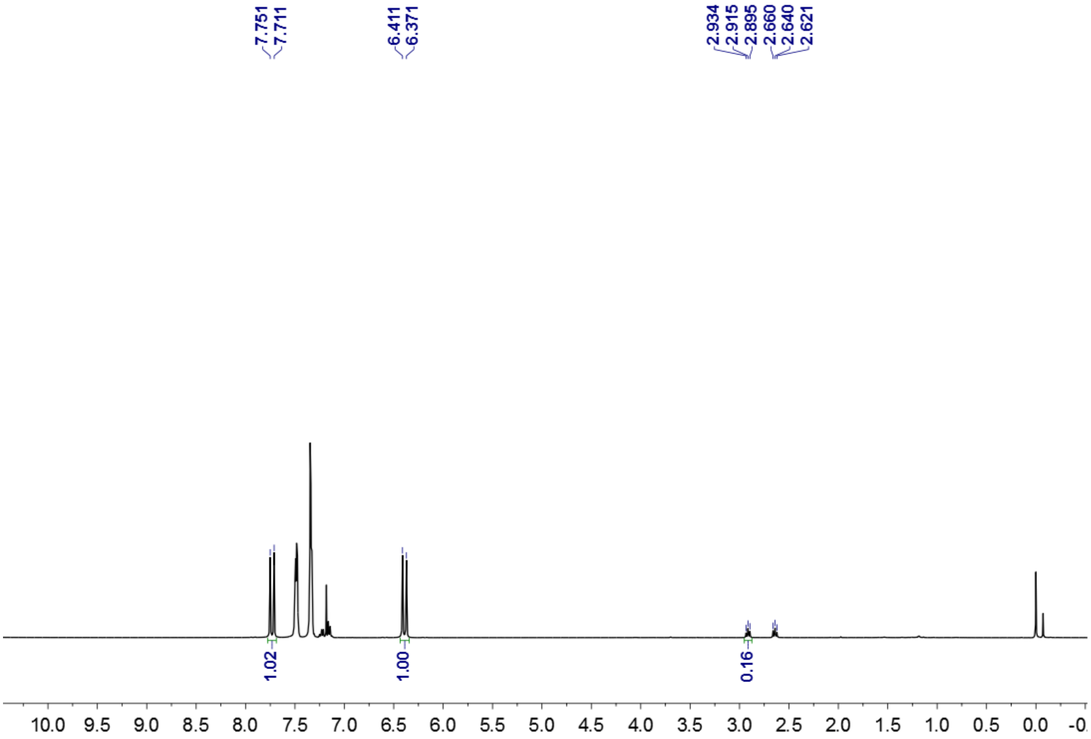

3-phenylpropanoic acid (2k) (in *Reaction Conditions E*)

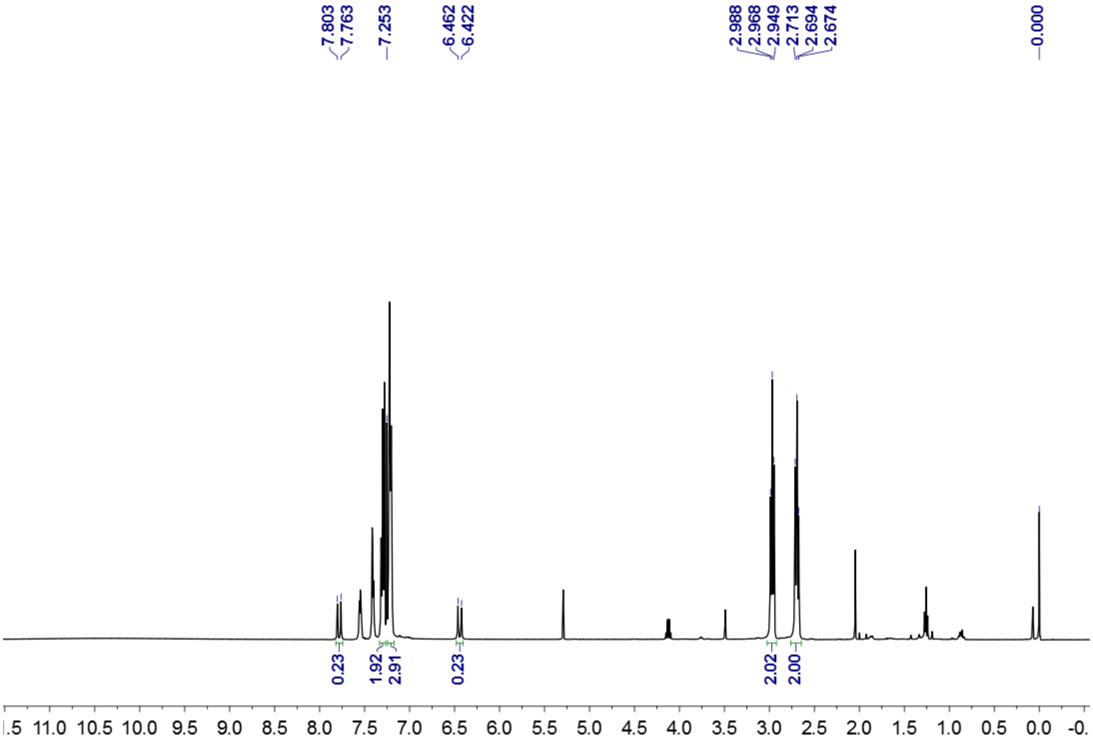

2-Phenylpropionic acid (2l) (in *Reaction Conditions D*)

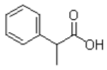

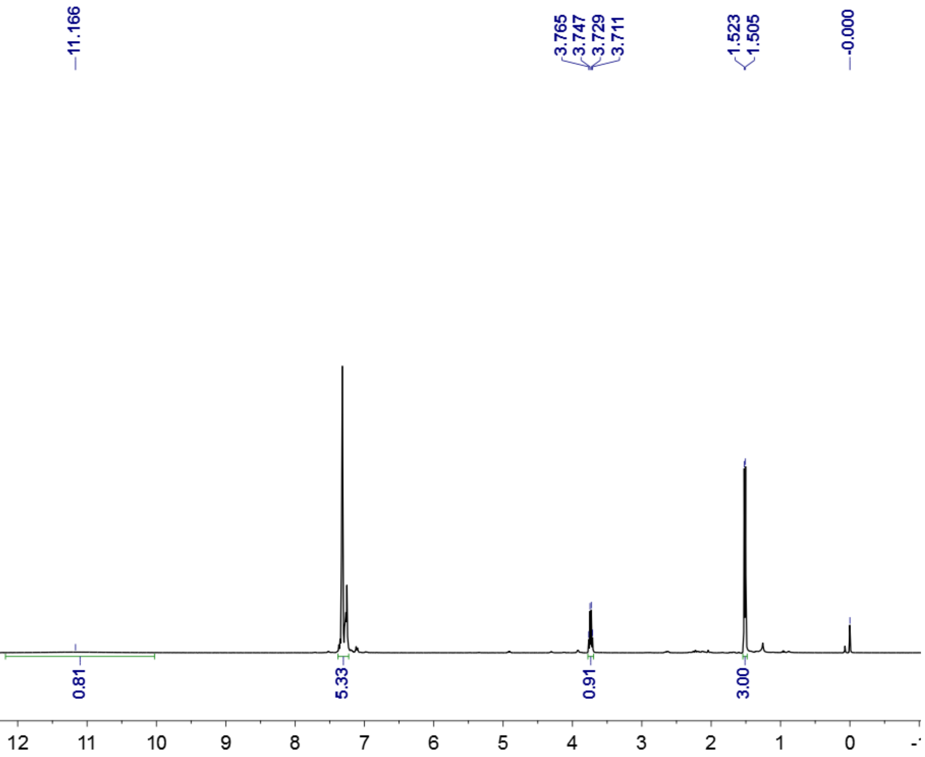

Ethyl 3-phenylpropanoate (2m) (in *Reaction Conditions C*)

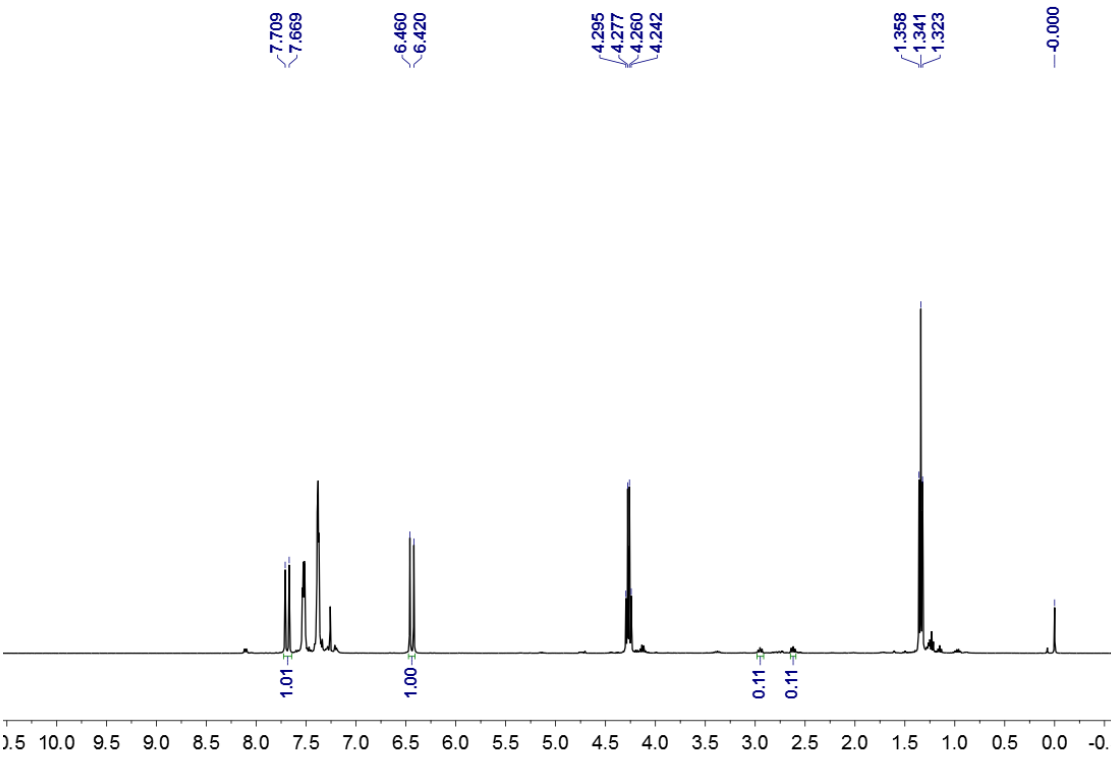

Ethyl 3-phenylpropanoate (2m) (in *Reaction Conditions E*)

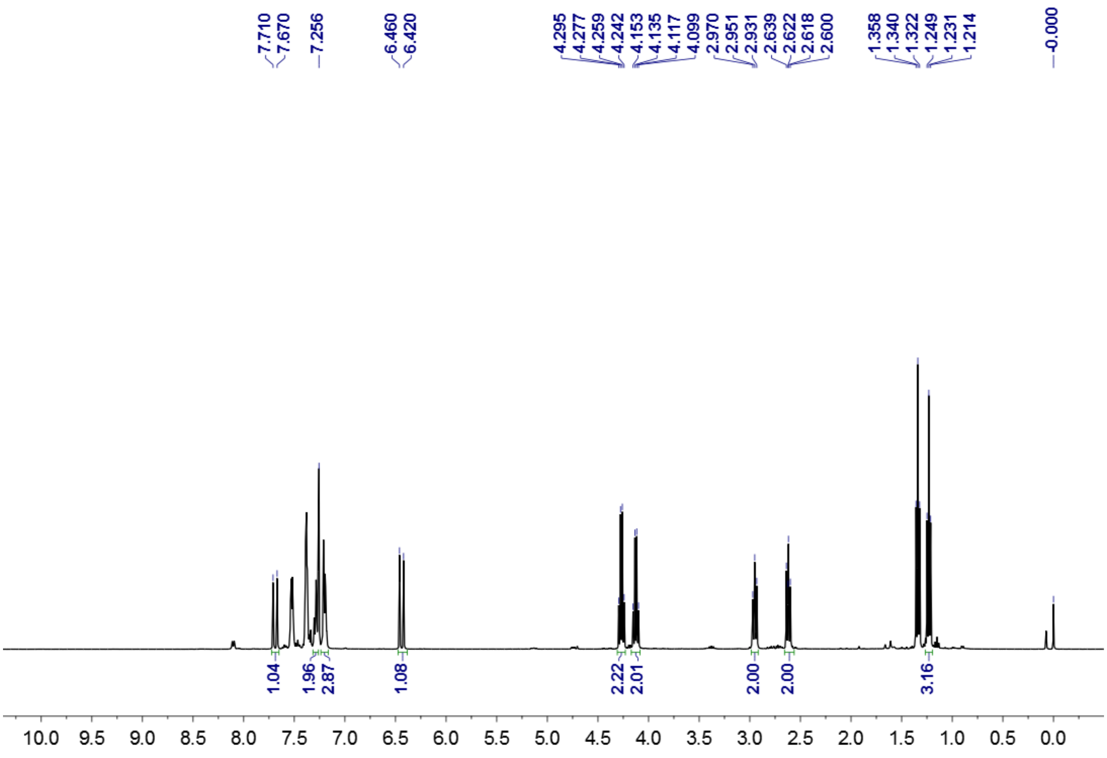

Diethyl 2-(2,4-dimethoxybenzyl)malonate (2n) (in *Reaction Conditions D*)

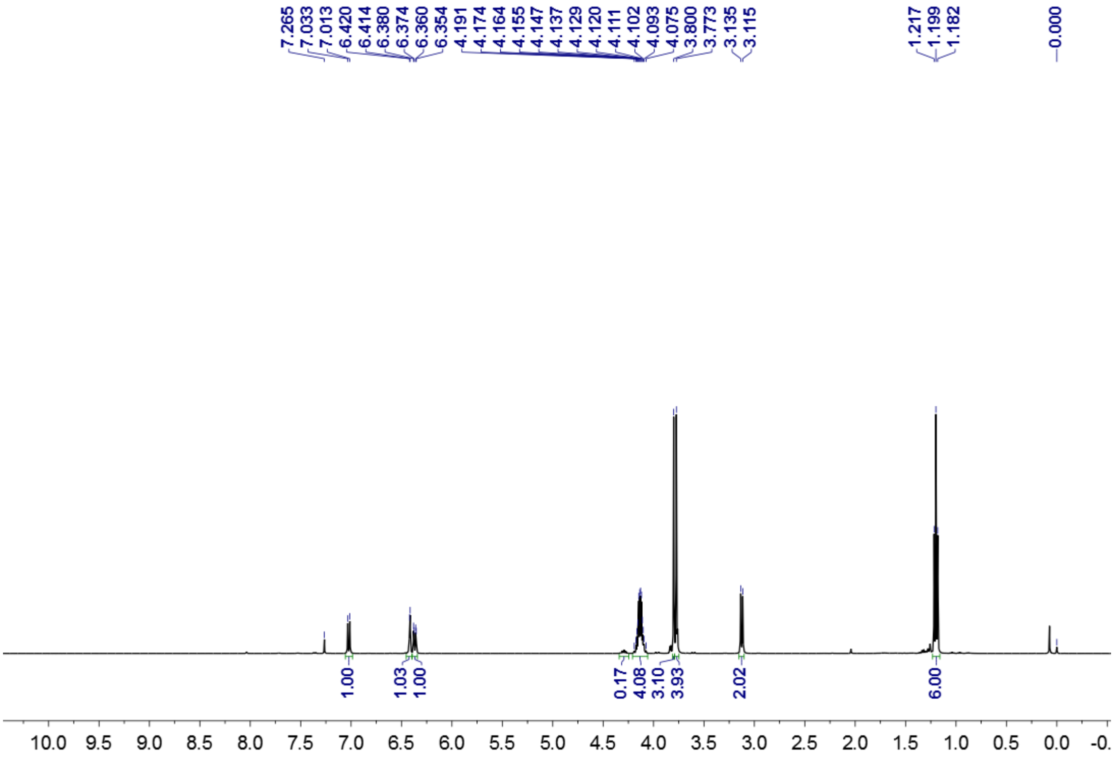

Dimethyl succinate (2o) (in *Reaction Conditions C*)

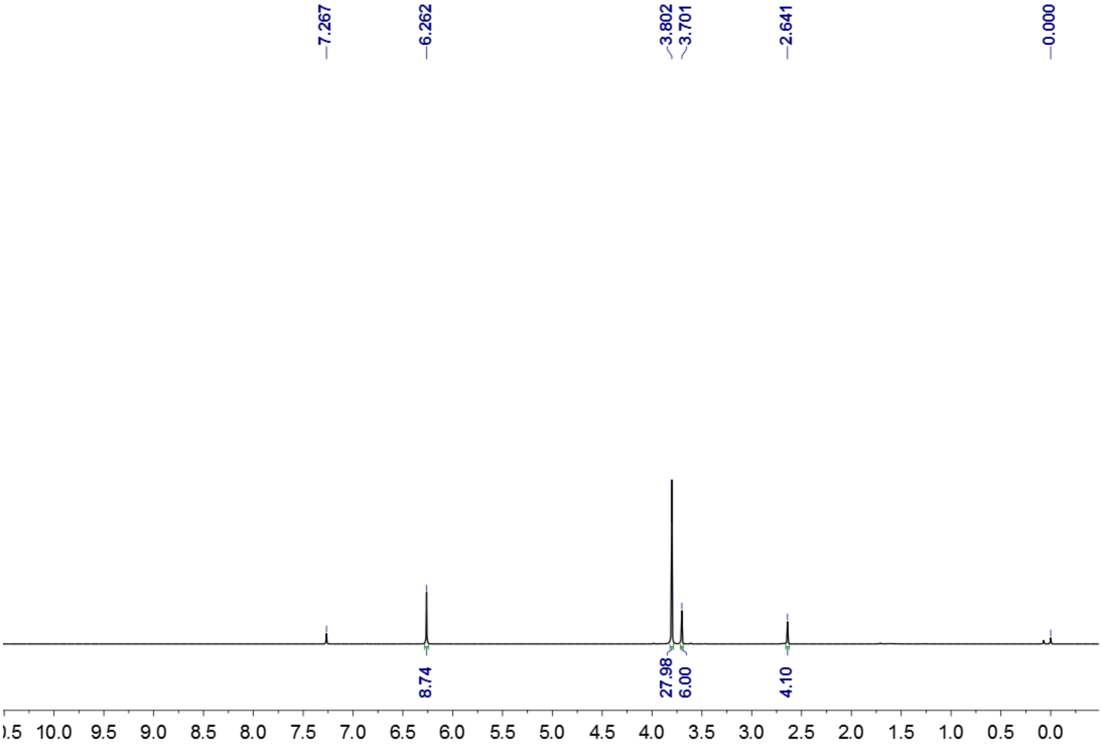

Dimethyl succinate (2o) (in *Reaction Conditions E*)

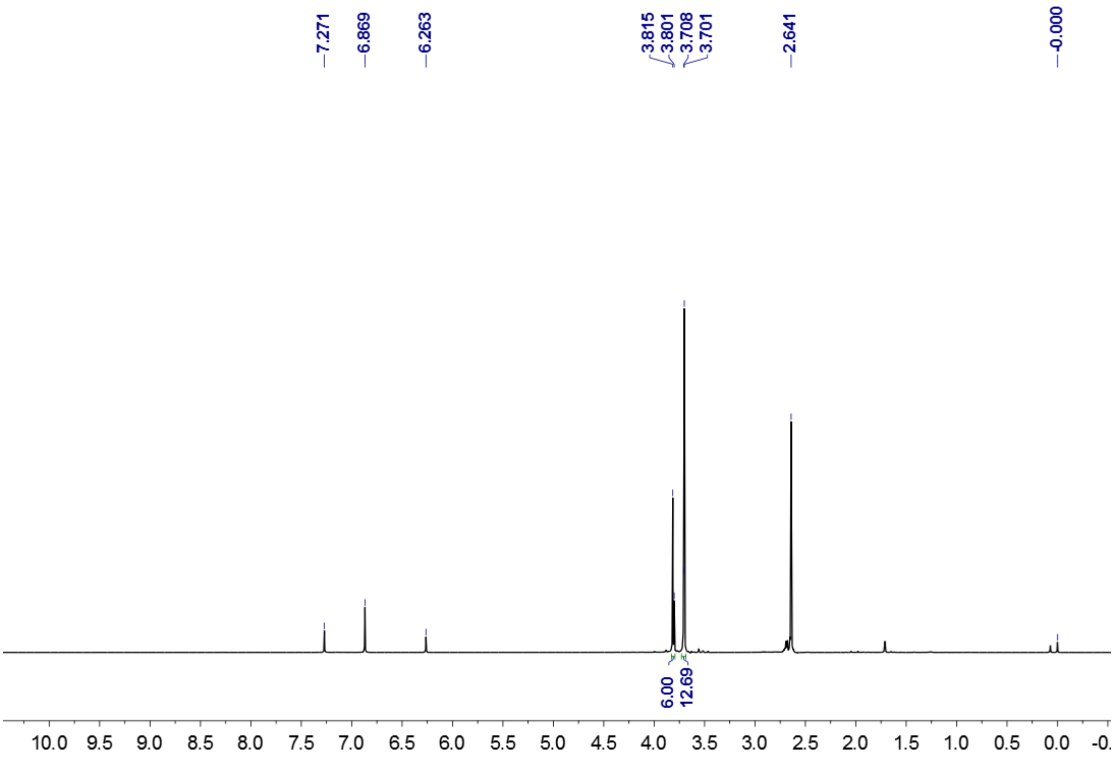

3-phenylpropanenitrile (2p) (in *Reaction Conditions D*)

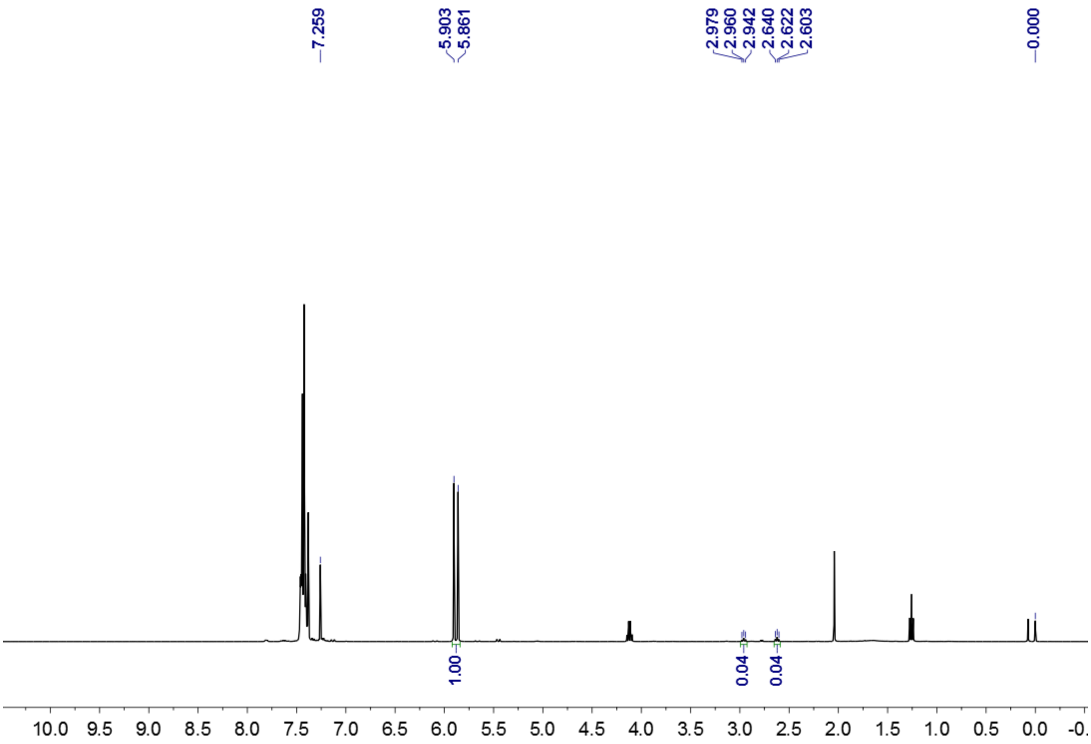

3-Phenylpropanenitrile (2p) (in *Reaction Conditions E*)

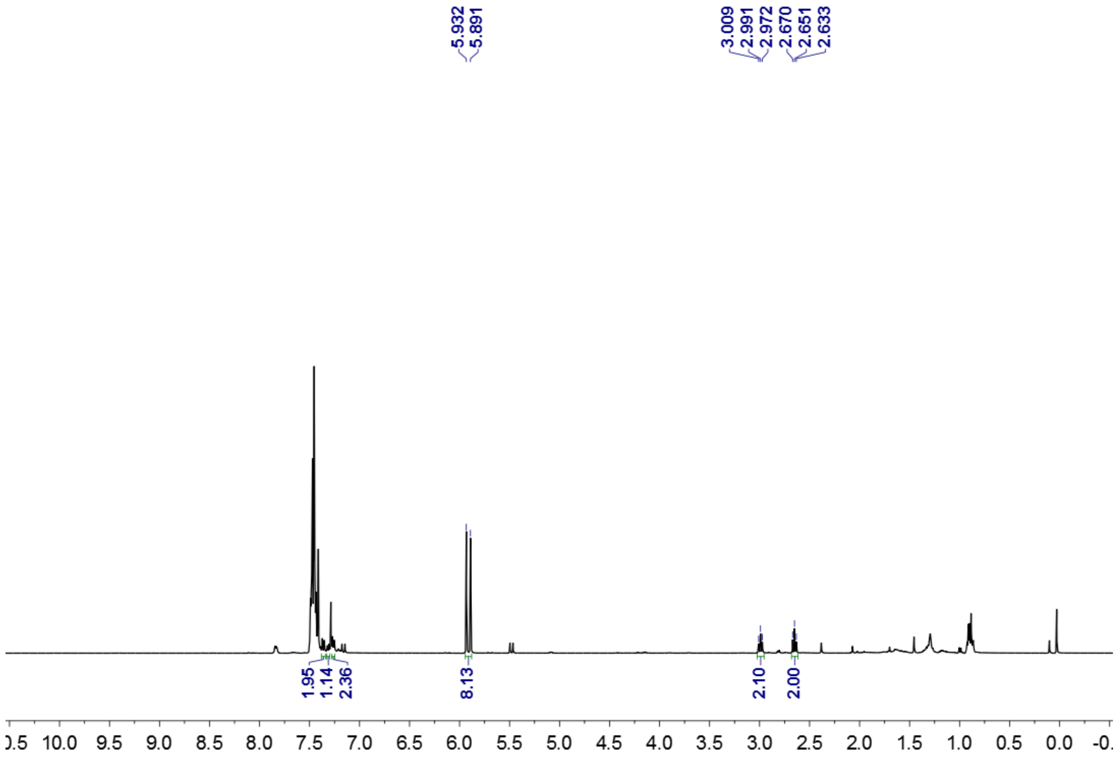

Supplement: Supplementary file 1 [file molecules-29-05912-s001.zip › molecules-3299485-supplementary.pdf]
